# Factoring and correlation in sleep, fatigue and mental workload of clinical first-line nurses in the post-pandemic era of COVID-19: A multi-center cross-sectional study

**DOI:** 10.3389/fpsyt.2022.963419

**Published:** 2022-08-25

**Authors:** Yan Liu, Ji Shu Xian, Rui Wang, Kang Ma, Fei Li, Fei Long Wang, Xue Yang, Ning Mu, Kai Xu, Yu Lian Quan, Shi Wang, Ying Lai, Chuan Yan Yang, Teng Li, Yanchun Zhang, Binbin Tan, Hua Feng, Tu Nan Chen, Li Hua Wang

**Affiliations:** ^1^Southwest Hospital, Administrative Office, Army Medical University, Chongqing, China; ^2^Department of Neurosurgery, Southwest Hospital, Army Medical University, Chongqing, China; ^3^Southwest Hospital, Army Aviation Medicine Teaching and Research Office, Army Medical University, Chongqing, China

**Keywords:** sleep quality, fatigue, mental workload, COVID-19, post-pandemic era, mental health, cross-sectional study

## Abstract

**Background:**

A better understanding of the factors and their correlation with clinical first-line nurses’ sleep, fatigue and mental workload is of great significance to personnel scheduling strategies and rapid responses to anti-pandemic tasks in the post-COVID-19 pandemic era.

**Objective:**

This multicenter and cross-sectional study aimed to investigate the nurses’ sleep, fatigue and mental workload and contributing factors to each, and to determine the correlation among them.

**Methods:**

A total of 1,004 eligible nurses (46 males, 958 females) from three tertiary hospitals participated in this cluster sampling survey. The Questionnaire Star online tool was used to collect the sociodemographic and study target data: Sleep quality, fatigue, and mental workload. Multi-statistical methods were used for data analysis using SPSS 25.0 and Amos 21.0.

**Results:**

The average sleep quality score was 10.545 ± 3.399 (insomnia prevalence: 80.2%); the average fatigue score was 55.81 ± 10.405 (fatigue prevalence: 100%); and the weighted mental workload score was 56.772 ± 17.26. Poor sleep was associated with mental workload (*r* = 0.303, *P* < 0.05) and fatigue (*r* = 0.727, *P* < 0.01). Fatigue was associated with mental workload (*r* = 0.321, *P* < 0.05). COVID-19 has caused both fatigue and mental workload. As 49% of nurses claimed their mental workload has been severely affected by COVID-19, while it has done slight harm to 68.9% of nurses’ sleep quality.

**Conclusion:**

In the post-COVID-19 pandemic era, the high prevalence of sleep disorders and fatigue emphasizes the importance of paying enough attention to the mental health of nurses in first-class tertiary hospitals. Efficient nursing strategies should focus on the interaction of sleep, fatigue and mental workload in clinical nurses. In that case, further research on solutions to the phenomenon stated above proves to be of great significance and necessity.

**Clinical trial registration:**

[https://clinicaltrials.gov/], identifier [ChiCTR2100053133].

## Introduction

During the COVID-19 pandemic, nurses often spend the greatest amount of time and have the most frequent direct contact with patients, who may have a fever and/or an infectious disease, and often shoulder almost the entire burden of nucleic acid testing (NAT) and sampling. In the case of long COVID-19 or post-sequelae infection and repeated outbreaks, which place a demand on the breadth of healthcare systems (1), nurses must manage and control the continuous development of the pandemic while caring for non-COVID-19 patients and protecting themselves from infection.

Based on a hospital’s ability to provide medical care, medical education and conduct health research, hospitals in China are designated as primary, secondary or tertiary institutions (2). Furthermore, based on the level of service provision, size, medical technology, medical equipment, and management and medical quality, these three grades are further subdivided into three subsidiary levels, resulting in a total of nine levels. A first-class tertiary hospital is more specialized than any other level (3). In particular, the clinical first-line nurses of first-class tertiary hospitals in China need to be ready to support health institutions in areas where outbreaks occur. This is bound to pose new challenges for nurses who are already in a high-risk and high-burden position for prevention and control during the normalization period of the pandemic.

China is among the first to enter the post pandemic ear of anti-COVID-19 (4), while the sleep quality, fatigue and mental workload of clinical nurses keep unknown. In the wake of the COVID-19 outbreak, the occupational pressure on medical staff has soared, and their sleep quality, fatigue status and mental health have also received extensive attention. A range of evidence has reported poor sleep quality and fatigue among medical workers during the COVID-19 pandemic. The incidence of insomnia ranges from 34.0 to 36.1% (5). The prevalence of fatigue among nurses was 35.06% (6), and the average mental workload score of nurses was 65.9 ± 12.71 (7). To provide new suggestions to managers to build more reasonable strategies to fight post COVID-19 stressors among healthcare professionals, the data on nurses’ sleep, fatigue and mental workload status and contributing factors need to be updated. Therefore, we took these three stressors as the main outcomes and tried to describe the current situation of nurses and determine the factors contributing to these stressors.

Previous research has demonstrated a correlation between sleep and fatigue, or sleep and mental workload, or fatigue and mental workload. Many evidence-based studies have shown that fatigue or insufficient sleep causes exhaustion, decreases concentration, and increases mortality (8). For nurses, disturbed sleep and fatigue lead to a higher risk of nursing errors (9). Similarly, a high mental workload has a negative impact on a person’s health and work safety, and causes issues such as mental health problems, mental distress and low job satisfaction (10).

However, little is known about the quantitative relationship among sleep, fatigue and mental workload. And there is a lack of studies evaluating all three simultaneously in clinical nurses. We believe that quantifying the inter-relationship among them may help optimize the health maintenance strategies of nursing staff and efficiently coordinate anti-epidemic human resources.

Therefore, in this study, we regard sleep quality, fatigue and mental workload as the main outcomes and explore their relationships. The main aims of this survey were as follows: (1) to describe the sleep quality, fatigue, and mental workload and the associated factors; (2) to assume there will be interaction among them; and (3) to clarify the impact of COVID-19 on nursing staff in first-class tertiary hospitals.

## Materials and methods

### Study design

A cluster sampling, multi-center, cross-sectional observational study was carried out from November 20th, 2021 to November 27th, 2021 in 3 first-class tertiary hospitals affiliated with the Army Military Medical University in Chongqing, China. The recommended minimum sample size was 315, based on the formula:


(1) $$\text{N}\;\text{=}\;{\text{Z}}_{\text{α}}^{2}\times \text{P}(1-\text{P})÷{\text{δ}}^{2}$$

where Z_α_ = 1.96 at a 95% CI with a 5% margin of error. The proportion (P) of the sample with insomnia was 71.8% (11) and the δ value in this study was 0.05 (12). Considering a 20% invalid survey response rate, the expected sample was at least 378. Finally, 1005 participants responded to the survey, and 1,004 of the 1,005 provided valid responses (response rate 99.9%).

We completed our investigation through Internet. Chinese Questionnaire Star^1^ was chosen to be the platform to deliver our questionnaire, meanwhile necessary Informed Consent Form would also be demonstrated. After that, we will acquire a QR code without Additional expenditure. Nurses logged on to *Questionnaire Star* via scanning the QR code with a smartphone or computer. There, they were able to read the 2-page informed consent form. After reading the consent form, the nurses could decide whether to take part in this survey. The subjects who agreed to participate in the study were required to electronically sign the consent form and then complete the questionnaires; otherwise, the *Questionnaire Star* tool would end the interview directly. Each participant was only allowed to submit once in order to avoid double submission. Volunteers who help to fill out our survey mentioned above will get rewards from Questionnaire Star. The reward amount would range from $ 5 to $ 10 at random.

### Study population

A notice for volunteers through the Nursing Management Offices of the three hospitals. The notice indicated the purpose of the survey, the precautions that need to be cooperated, and the inclusion and exclusion criteria.

Inclusion criteria included the following: (1) registered nurses who had worked in their current hospitals since the outbreak of COVID-19 in China; (2) nurses with the ability to use basic information technology such as a computer and the WeChat App; (3) nurses who volunteered to participate and signed an informed consent form.

Exclusion criteria included the following: (1) nurses who had not worked fulltime for the past month for any reason and (2) nurses with physical or mental illnesses (e.g., depression or epilepsy).

### Collection of the demographic data

A total of 1,005 questionnaires, including 1 unqualified data, were collected (efficient rate of 99.9%). There were 29 sociodemographic variables evaluated in this study, the details of which are provided in Supplementary material.

### Measurement of sleep quality, fatigue, and mental workload

#### Sleep quality assessment

The Pittsburgh Sleep Quality Index (PSQI) was used to evaluate the sleep quality of the eligible nurses. The PSQI is a mature scale that is widely used to assess the sleep quality of people with no special restrictions. Its reliability and validity have been evaluated by many studies (13). The scale has 7 items: sleep latency, sleep duration, sleep quality, habitual efficiency, sleep disturbance, daytime dysfunction and the use of sleeping medications. Total PSQI scores can range from 0 to 21: 8 points or more indicate a sleep disorder, 8-10 points indicate a minor sleep disorder, 11-15 points indicate a serious sleep disorder, and 16 points or more indicate a severe sleep disorder (14).

#### Fatigue assessment

The Modified Fatigue Impact Scale (MFI-20) was used to assess the participants’ fatigue in the past 4 weeks. This 20-item scale can be categorized into 5 dimensions: Comprehensive fatigue, physical fatigue, activity reduction, physical decline and mental fatigue. Total fatigue scores can range from 0 to 100 and are interpreted as no fatigue (0-20), minor fatigue (21-39), serious fatigue (40-59), severe fatigue (60-79) and extremely severe fatigue (80-100) (15). This scale was used to measure fatigue in Chinese population with a good validity and reliability (16).

#### Mental workload assessment

The NASA task load index (NASA TLX) (17) is a tool for measuring and conducting a subjective mental workload (MWL) assessment. It rates performance across six dimensions to determine an overall workload rating. The six dimensions are as follows: mental demand, physical demand, temporal demand, frustration level and effort and performance. The total MWL score ranges from 0 to 100 points. The higher the score is, the higher the mental workload is. This scale has been *validated* by Chinese scholars and can be used to test the mental workload of Chinese nurses (18). In this study, we used the weighted workload score.

### Statistical analyses

First, all answer sheets were exported from *Questionnaire Star* to SPSS 25.0 and error-checked by the study team. Then, all data were divided into measurement data and classification data, and the QQ chart and Bonferroni test were used to check the normality and homogeneity of the variance.

Second, for the descriptive analysis of the participants’ sociodemographic data, the average standard deviation was evaluated for measurement data such as age, height, and weight, and the frequency was assessed for classification data such as sex, length of service, and education. Multi-correspondence analysis was conducted for one multichoice question, “Which time periods may be the most fatiguing when a nurse is on night shift duty?”

Third, for the distribution of the three scales (the MFI-20, PSQI, and NASA-TLX), the mean and standard deviation were calculated to determine whether the scores conformed to a normal distribution. However, the mean (max, min) was used to analyze the score distributions that did not conform to a normal distribution, such as the score for frustration level (a NASA-TLX component) and that of sleep duration and the use of sleeping medication (PSQI components). The frequency was used to analyze the severity of fatigue and insomnia.

Fourth, path analysis was conducted by IBM Amos 21.0 to check the correlations among sleep, fatigue and mental workload. A result was considered significant when two-tailed *P* values were < *0.05*. The standardized regression weights (β values) present the correlation. Factor weights between 0.5 and 0.95 indicate that the measured variable contributes well to the latent variable. According to Jackson’s recommendation (19) and Rappaport’s suggestion (20), seven model fit indices were reported as followed: Chi-square (X^2^), degrees of freedom (df), goodness-of-fit index (GFI), adjusted goodness-of-fit index (AGFI), Comparative fit index (CFI), Root-mean-square error of approximation (RMSEA) and Bayesian information criteria (BIC) values. When GFI, AGFI and CFI were more than 0.9, the result could be described as good fit. Besides, a model is considered acceptable when those indices mentioned above were above 0.8 RMSEA being lower than 0.05 indicated a good model fit, while lower than 0.08 is considered acceptable. A result of good model fit could also be identified when BIC value was lower than both saturated and independence model.

Finally, to determine the factors affecting the target outcomes and control the influence of confounding factors, stepwise linear regression analysis was conducted. When the VIF value was *< 10.0*, the factor was considered to present a low level of interference. The standardized coefficient beta indicated the degree of impact the factors had on the dependent variables: The higher the value was, the greater the degree of influence.

### Ethical considerations

Guided by the 2000 Declaration of Helsinki for ethical standards, the protocol has been approved by the Ethics Committee of the First Affiliated Hospital of Army Medical University, PLA (Ref: KY2021062).

## Results

### Descriptive statistics of nurse characteristics

**FIGURE 1:**
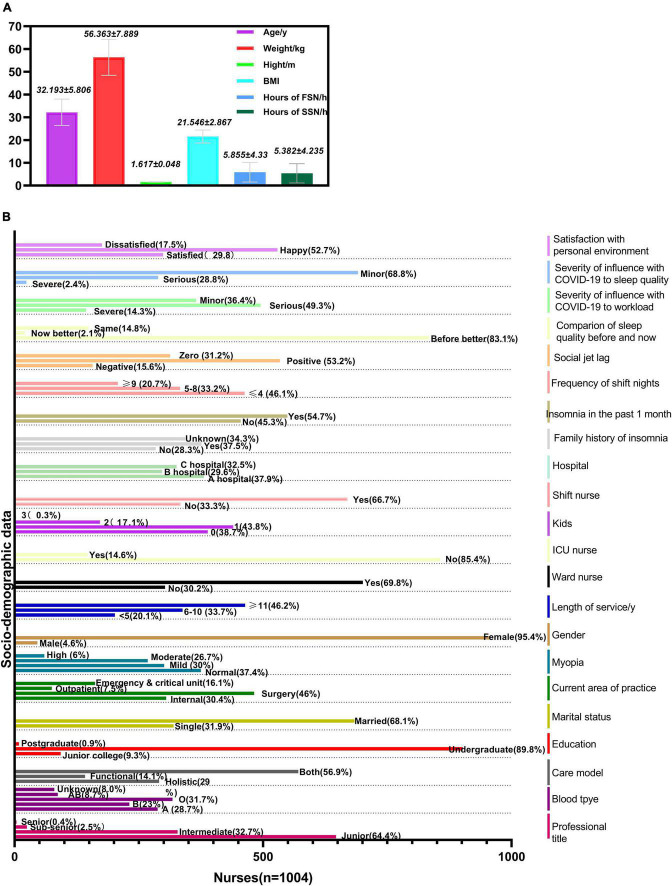
Descriptive analysis of sociodemographic data (*n* = 1004). FSN, first shift night; SSN, second shift night. **(A)** The mean and standard deviation of demographic data of nurses. **(B)** The percentage of nurses on socio-demographic data.

In total, 1,005 clinical first-line nurses of three tertiary hospitals responded to the survey without any missing data and their demographic characteristics were detailed in Figure 1. Figure 1A shows the mean and standard deviation of data that were the numerical variables. Figure 1B shows the percentage of nurses with sociodemographic data, which were categorical variables.

### Distribution of total scores of three scales and their component scores

The Cronbach’s alpha coefficient was 0.829 for NASA-TLX, 0.556 for PSQI, and 0.771 for MFI-20. Cronbach’s alpha coefficients indicated that the NASA-TLX, PSQI, and MFI-20 had a satisfactory internal consistency.

The average total PSQI score was 10.545 ± 3.399, in which the average sleep quality score was 1.617 ± 0.814, sleep latency score was 1.761 ± 0.953, habitual efficiency score was 2.224 ± 0.913, sleep disturbance score was 1.412 ± 0.677, and daytime dysfunction score was 1.994 ± 0.821. The mean (max, min) sleep duration score and the use of sleeping medication score, which are components of the PSQI, were 1.36 (0, 3) and 0.171 (0, 3), respectively. The insomnia incidence of the nurses was 80.8% (*n* = 811). In the past month, 80.4% of the nurses had a PSQI score higher than 8, and only 19.2% of the nurses had PSQI scores lower than 7 with normal sleep. The percentages of nurses with degrees of insomnia were as follows: Serious insomnia (41.2%, *n* = 414), minor insomnia (31.7%, *n* = 318) and severe insomnia (7.9%, *n* = 79).

The average fatigue score was 55.81 ± 10.405, of which the average comprehensive fatigue score was 13.218 ± 3.149, physical fatigue score was 11.843 ± 3.039, activity reduction score was 9.754 ± 2.551, physical decline score was 10.607 ± 2.679 and mental fatigue score was 10.388 ± 2.941. The fatigue rate of the nurses in this survey was 100%, mainly including serious fatigue (53.4%, *n* = 536) and severe fatigue (37.6%, *n* = 378).

The weighted score of mental workloads was 56.772 ± 17.26, in which the mental demand score was 7.908 ± 7.157, physical demand score was 12.603 ± 9.716, temporal demand score was 10.511 ± 7.688, effort score was 9.466 ± 6.815 and frustration level score [mean, (max, min)] was 6.582 (0, 33.33).

**FIGURE 2:**
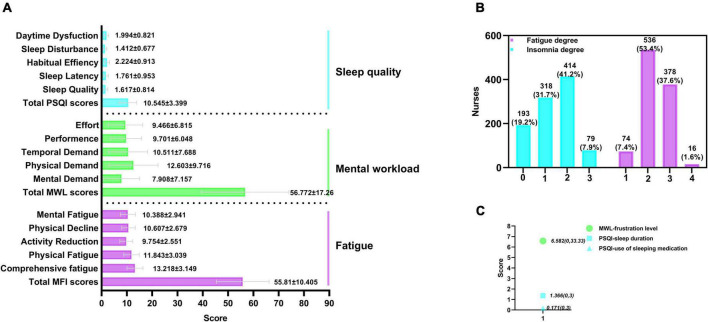
Distribution of total scores of three scales and their component scores (*n* = 1004). MFI, multiple fatigue impact scale; PSQI, the Pittsburg Sleep Quality Index; MWL, the NASA task load index; 0, normal; 1, minor; 2, serious; 3, severe; 4, extremely severe. **(A)** Distribution of total scores of MFI, MWL, and PSQI, and their component scores. **(B)** Percentage of the degree of insomnia and fatigue. **(C)** Analysis of the component score of MWL and PSQI [(mean, (max, min)], which did not conform to the normal distribution.

The abovementioned results are detailed in Figures 2A-C.

### Interrelationships among sleep, fatigue and mental workload with path analysis

**FIGURE 3:**
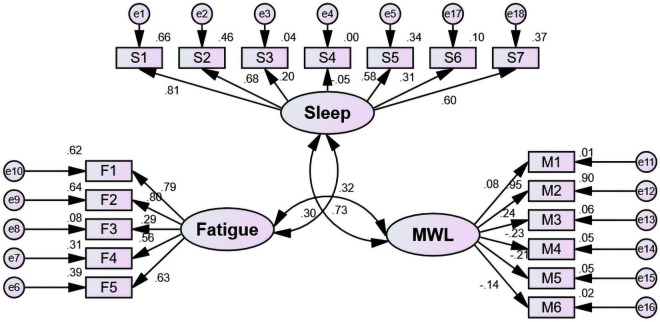
Model of the correlation among sleep, fatigue and mental workload in frontline nurses (*n* = 1004). MWL, mental workload; S1, sleep quality; S2, sleep latency; S3, sleep duration; S4, sleep efficiency; S5, sleep disturbance; S6, hypnotic drug; S7, daytime dysfunction; M1, mental demand; M2, physical demand; M3, temporal demand; M4, performance; M5, effort; M6, frustration level; F1, comprehensive fatigue; F2, physical fatigue; F3, activity reduction; F4, motivation decline; F5, mental fatigue.

To test the hypotheses that there will be interaction among sleep, fatigue and mental workload, Path Analysis was conducted through IBM Amos 21.0. Sleep, fatigue and mental workload were the three latent variables. The dimensions of the PSQI, including sleep quality, sleep latency, sleep duration, sleep efficiency, sleep disturbance, hypnotic drugs and daytime dysfunction, were regarded as the observed variables for *sleep*. The dimensions of the MFI-20, including comprehensive fatigue, physical fatigue, activity reduction, physical decline and mental fatigue, were regarded as the observed variables for *fatigue*. The dimensions of the NASA-TLX, including mental demand, physical demand, temporal demand, performance, effort and frustration level, were regarded as the observed variables for *mental workload*. The final structural model was presented in Figure 3.

The values pertaining to the model were that X^2^ was 855.409, df was 132, GFI was 0.908 (> 0.9), AGFI was 0.880 (> 0.8), CFI was 0.805 (> 0.8), RMSEA was 0.074 (< 0.08) and BIC value was 1,124.967 (saturated model: BIC = 1181.909; independence model: BIC = 3996.345). All indices indicated acceptable model fit.

Based on the analysis detailed in Table 1, the covariances of sleep, fatigue and mental workload were significantly different (*P* < 0.05). There was a positive correlation among them. The correlation coefficient between sleep and mental workload was 0.303, that between sleep and fatigue was 0.727, and that between mental workload and fatigue was 0.321.

**TABLE 1:** Correlation coefficient, covariance and covariance significance among sleep, fatigue and mental workload (*n* = 1004).

	Correlations estimate	Covariances			
		Estimate	*S.E.*	*C.R.*	*P*
Sleep ↔ Mental Workload	0.303	0.112	0.053	2.125	0.034
Sleep ↔ Fatigue	0.727	0.883	0.068	12.954	***
Mental Workload ↔Fatigue	0.321	0.333	0.156	2.129	0.033

***P < 0.001; SE, Standard error; CR, Critical ratio.

The significance of all unstandardized regression coefficients except sleep efficiency was found (Table 2). The observed variables that contributed significantly to *sleep* included sleep quality (β = 0.809), sleep latency (β = 0.679), sleep disturbance (β = 0.582), and daytime dysfunction (β = 0.605). The observed variable that contributed significantly to *mental workload* was physical demand (β = 0.948). The observed variables that contributed significantly to *fatigue* included comprehensive fatigue (β = 0.785), physical fatigue (β = 0.802), physical decline (β = 0.561), and mental fatigue (β = 0.627).

**TABLE 2:** Path coefficients, unstandardized regression coefficients and their significance (*n* = 1004).

	Standardized estimate	Unstandardized			
		Estimate	*S.E.*	*C.R.*	*P*
S1 ← Sleep	0.809	1			
S2 ← Sleep	0.679	0.981	0.049	20.109	***
S3 ← Sleep	0.199	0.447	0.078	5.75	***
S4 ← Sleep	−0.046	−0.064	0.048	−1.323	0.186
S5 ← Sleep	0.582	0.597	0.035	17.218	***
S6 ← Sleep	0.309	0.26	0.029	8.989	***
S7 ← Sleep	0.605	0.753	0.042	17.927	***
M1 ← Mental Workload	0.078	1			
M2 ← Mental Workload	0.948	16.397	7.449	2.201	0.028
M3 ← Mental Workload	0.243	3.329	1.474	2.258	0.024
M4 ← Mental Workload	−0.229	−2.471	1.101	−2.245	0.025
M5 ← Mental Workload	−0.215	−2.604	1.169	−2.228	0.026
M6 ← Mental Workload	−0.144	−1.687	0.811	−2.081	0.037
F5 ← Fatigue	0.627	1			
F4 ← Fatigue	0.561	0.815	0.055	14.793	***
F3 ← Fatigue	0.290	0.401	0.049	8.21	***
F2 ← Fatigue	0.802	1.322	0.069	19.097	***
F1 ← Fatigue	0.785	1.341	0.071	18.896	***

***P < 0.001; SE, Standard error; CR, Critical ratio; S1, Sleep quality; S2, Sleep latency; S3, Sleep duration; S4, Sleep efficiency; S5, Sleep disturbance; S6, Hypnotic drugs; S7, Daytime dysfunction; M1, Mental demand; M2, Physical demand; M3, Temporal demand; M4, Performance; M5, Effort; M6, Frustration level; F1, Comprehensive fatigue; F2, Physical fatigue; F3, Activity reduction; F4, Motivation decline; F5, Mental fatigue.

### Stepwise linear regression of the factors associated with the target outcomes

**FIGURE 4:**
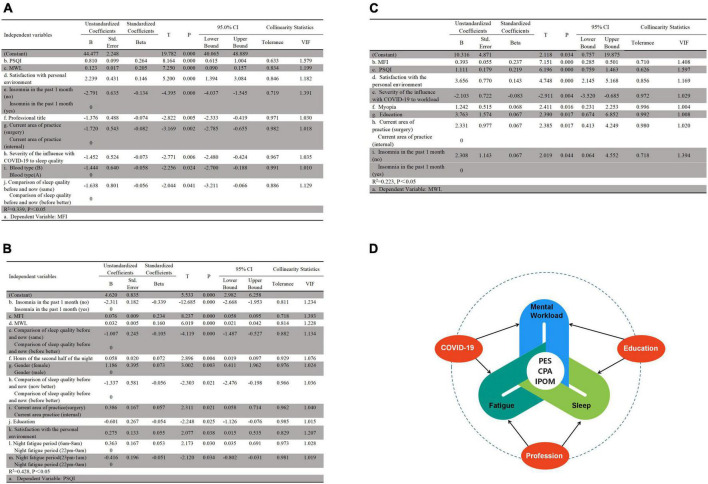
Stepwise linear regression of the factors associated with the target outcomes (*n* = 1004). MFI, multiple fatigue impact scale; PSQI, the Pittsburg Sleep Quality Index; MWL, the NASA task load index; PES, personal environment satisfaction; CAP, current area of practice; IPOM, insomnia in the past one month. **(A)** Factors associated with MFI. **(B)** Factors associated with PSQI. **(C)** Factors associated with MWL. **(D)** Common influencing factors of MFI, PSQI and MWL.

Figure 4A shows the influencing factors of the fatigue. Among all the independent variables, nine variables were successfully included in the model (*R*^2^ = 0.339, 95% *CI*: 40.065 to 48.889). Based on the analysis, the regression equation, which is between the dependent variable (the MFI score) and the independent variables (influencing factors), was as follows:


$$\begin{array}{l} a=44.477+0.81*b+0.123*c+2.239*d-2.791*e\\ \;-1.376*f-1.72*g-1.452*h-1.444*i-1.638*j \end{array}$$

Figure 4B shows the influencing factors of the sleep. Among all the independent variables, twelve variables were successfully included in the model (*R*^2^ = 0.428, 95% *CI:* 2.982 to 6.528, *P* < 0.001). Based on the analysis, the regression equation, which is between the PSQI score and influencing factors, was as follows:


$$\begin{array}{l} a=4.62-2.311*b+0.076*c+0.032*d-1.007*e\\ \;+0.588*f+1.186*g-1.337*h+0.386*i\\ \;-0.601*j+0.275*k+0.363*l-0.416*m \end{array}$$

Figure 4C shows the influencing factors of the mental workload score. Among all the independent variables, there were 8 variables finally included in the model (*R*^2^ = 0.223, 95% *CI:* 0.757 to 19.875, *P* < 0.001). Based on the analysis, the regression equation, which is between the mental workload score and influencing factors, was as follows:


$$\begin{array}{l} a=10.316+0.393*b+1.111*c+3.656*d-2.103*e\\ \;+1.242*f+3.763*g+2.331*h+2.308*i \end{array}$$

Figure 4D shows the common influencing factors of the fatigue, sleep and mental workload. The study found that three factors, including personal environment satisfaction, insomnia in the past month and current area of practice, were common influencing factors of the three. The joint influencing factor of the mental workload and sleep was education, that for the mental workload and fatigue was COVID-19, and that for the fatigue and sleep was the comparison of nurses’ sleep quality before the pandemic and at the time of this study. There was a mutual relationship among the fatigue, sleep and mental workload.

## Discussion

This multicenter cross-sectional study aimed to update the information about sleep, fatigue and mental workload in hospital nurses in China during the normalized pandemic era of COVID-19, to quantify the interrelation among the three and to explore the related factors of them. Thus, we came across these findings as below.

### Fatigue and associated factors

We found that front-line clinical nurses suffered from varying degrees of fatigue, and the positive detection rate of fatigue was 100%. This was much higher than both before and during the pandemic of COVID-19 (6, 21). Different from other occupations, the fatigue of nurses is mainly comprehensive fatigue and physical fatigue. This may contribute to the high physical workload (22).

High fatigue prevalence and the reasons behind it are thought provoking. We found these factors may cause different level of fatigue: poor sleep, lower satisfaction of personnel environment, professional title, current department of practice, blood type, and COVID-19.

Poor sleep may lead to fatigue, which manifested daytime dysfunction (23). Likewise, the more tired the body is, the less sleep it may be. It is because excessive fatigue can easily cause autonomic dysfunction that the nerves are in a state of excitement, so as to insomnia (24).

Personnel environment is one component of job satisfaction (25). Lower satisfaction of personnel environment might decrease motivation to work and increase physical and mental fatigue. Nursing managers should pay attention to the personnel environment, try to create a happy personnel culture for their nurse staff.

Professional title and current department of practice were also factors of fatigue. The lower the job title was, the more fatigue would be. Because nurses’ job title had a positive correlation with their length of experience (*r* = 0.630, *P* < 0.001). The nurses with a low job title would be in younger experience of nursing, they might lack clinical experience so that spend more time and energy dealing with clinical problem. Likewise, nurses in surgery department were more fatigue than those in internal department. This might be due to differences in work content and work intensity. The hospitals we investigated were all first-class tertiary hospitals in China, which often undertake the diagnosis and treatment of many patients who need major surgeries. Surgery nurses in these hospitals usually require more physical effort to care for preoperative and postoperative patients and more mental effort to prevent and manage postoperative complications.

### Sleep and associated factors

Using PSQI to assess sleep quality of nurses, we found a higher incidence (80.2%) of insomnia both before COVID-19 and during the epidemic (26–28). In addition, 54.7% of nurses self-reported sleep complaints in past one month. The sleep problems of clinical front-line nurses mainly manifest as low sleep efficiency, daytime dysfunction and difficulty falling asleep.

Sleep quality is due to multisystem factors (8, 29), including the neural system, lifestyle, job environment, and physical and psychological factors. We found that these factors may cause sleep disorders: nursing occupation, gender, current department of practice, education, lower satisfaction of personal environment and shift nights.

Occupation of nursing itself may cause sleep disorders. We found in this study 83.1% of nurses reported their sleep quality before they became a nurse were better than after that. Because nursing is a such profession that they have to take more frequent shift work. Li found the prevalence of shift work sleep disorder in the sampled shift nurses was 48.5% (30). We also found that nurses suffered most fatigue at 23 pm to 0 mn and 6 am to 8 am when they were on night shift duty. This indicated shift nights may exacerbate sleep disorders (31).

Women’s sleep quality is significantly worse than men’s. Rani (32) reported that higher odds of sleep problems among women in China. Same to resent studies (33, 34), we found women’s worse sleep mainly manifested as worse subjective sleep quality, long time sleep latency and short sleep duration. The reason for this difference may be related to the development of the brain during childhood (35).

Education level affects sleep quality, and lower level may contribute more to sleep problem. Resent study (36) pointed that educational attainment has positive effects on cognitive function. The person with higher education level may have the ability to learn more skills to improve their sleep quality. They may also have a better cognition to develop good sleep hygiene habits. Thereby, nursing managers should encourage their nurses to improve their education attainment.

Nurses practicing in surgery department are pond to worse sleep than those in internal department (37). As the saying goes: three points of treatment, seven points of care, especially for clinical surgery nurses. Comparison with the internal nurses, they would provide service to postoperative patients who may occur complications at any time. Clinical surgery nurses are always more carefully to care their patients so that they always burden a severe mental pressure although they are out of duty.

Lower satisfaction of personal environment may contribute more to insomnia. Number of studies pointed a positive correlation between job satisfaction and sleep (25, 38–40). A health personnel environment can reduce job stress, improve sleep quality. We believe that nursing leaders should focus on the strategies for developing a health personnel culture.

Also, poor sleep may cause some negative outcomes, including more fatigue (41), higher mental workload (42). And we found that insomnia may increase subjective sleep complaints. It is important for leaders to listen to and encourage nurses to self-report sleep problems to reduce nursing safety risks associated with sleep disorders.

### Mental workload and its associated factors

We found weighted mental workload score (56.772 ± 17.26) was lower than that previously reported during the outbreak of COVID-19(65.9 ± 12.71) (7) and before the pandemic (80.48 ± 11.76) (43). This change may be ascribed to the decline in hospitalized COVID-19 patients. Most nurses regarded physical demand as the most important component of their mental workload, followed by temporal demand or performance, which was different from a recent study that found the time demand to be the most important component, followed by mental demand and physical demand (44).

The reasons, this study found, which may cause high level of mental workload, including lower satisfaction of personal environment, COVID-19, myopia, current department of practice and poor sleep. Contrary to Boultinghouse’ report of physician (45), we found there was a positive correlation between nurses’ mental workload and personnel environment. This discrepancy may indicate a more complex personnel environment for nurses. Working in such a complex personnel environment, nurses may need to spend so much energy dealing with personal relationships, that they will struggle to complete clinical nursing tasks. In addition, clinical surgery nurses reported a higher mental workload score than medical nurses. This may be due to the different work content and work intensity before mentioned. Similar to a previous study, we also found that COVID-19 itself contributed to mental workload (46).

### The correlation among sleep, fatigue, and mental workload

Most previous studies have only demonstrated the relationship between sleep and fatigue (47, 48), or fatigue and mental workload (42, 49), or sleep and mental workload (31, 50). A recent study (51) investigated the fatigue, sleep and mental workload of shift night workers. But it still just addressed the relationship between fatigue and mental workload instead of all the three. It was possible that the authors assessed sleep with the participants’ self-report instead of scales or wearable devices. However, we quantified the inter-relationship of sleep, fatigue and mental workload. The positive correlation of the three indicated they could improve each other. This finding might provide a new perspective to managers and supervisors. That is, when assessing nurses’ mental health, sleep quality, fatigue and mental workload should be taken into account together rather than solely.

In addition, with path analysis, we found that the dimensions of the PSQI, including sleep quality (β = 0.81), sleep latency (β = 0.68), sleep disturbance (β = 0.58) and daytime dysfunction (β = 0.60), contributed significantly to the sleep index; the dimensions of the MFI-20, including comprehensive fatigue (β = 0.79), physical fatigue (β = 0.80), physical decline (β = 0.56) and mental fatigue (β = 0.63), contributed greatly to fatigue; and the physical demand (β = 0.95) dimension of the NASA-TLX contributed most to mental workload. This meant that sleep quality, sleep latency, sleep disturbance and daytime dysfunction in sleep, comprehensive fatigue, physical fatigue, physical decline and mental fatigue in fatigue and physical demand in mental workload had a positive and direct correlation. Therefore, given the interplay of different manifestations of sleep, fatigue, and mental workload, targeted interventions are recommended for more effective management of the associated risks.

### Predictors among sleep, fatigue, and mental workload

Figure 4D, a general diagram, presents the factors associated with the main outcomes in this study and the relationships among them.

The common risk factor for mental workload and sleep was education, and for mental workload and fatigue, it was COVID-19. In this study, education was positively correlated with mental workload but negatively correlated with sleep. This meant that the higher the education level was, the greater the mental workload and the worse sleep was.

In addition, the comparison of sleep quality before and after the COVID-19 pandemic—better before, better now, or the same—was influenced by the joint factor of sleep and fatigue. Regardless of whether the nurses had the same or better sleep quality, compared to better sleep before, it was negatively correlated with both fatigue and poor sleep. This indicates that the nursing profession itself has an impact on sleep and fatigue.

Furthermore, we also found that personnel environmental satisfaction, insomnia in past one month and current area of practice were correlated with sleep, fatigue and mental workload. Personnel environmental satisfaction, which was detailed as satisfied, happy, and dissatisfied, in that order, had a positive correlation with poor sleep, fatigue and mental workload. This meant that the more dissatisfied the nurses were with their personal environment, the worse their sleep and the greater their fatigue and mental workload. This was similar to a previous study, except that the study reported job satisfaction rather than personnel environmental satisfaction (10). Thereby, when intervening on sleep, fatigue, and mental workload, the focus should be on the comprehensive assessment and effective coping of personnel environment, self-reported insomnia and practicing department.

### The impact of COVID-19 on clinical frontline nurses

COVID-19 was a predictor of both mental workload and fatigue. The COVID-19 pandemic has certainly had multifaceted and vigorous psychological and mental impacts on nurses, such as anxiety, depression, and stress (52). Our study also found that the influence of COVID-19 on sleep or workload had a profound impact on the fatigue and mental workload of the nurses; that is, the greater the impact of COVID-19 was, the higher the level of fatigue and mental workload. A prior study reported that COVID-19 and workload are variables that significantly predict psychological discomfort (44), but did not mention the relationship between COVID-19 and fatigue.

As of November 20, 2021, according to a report from the National Health Commission of the People’s Republic of China, there had been 1,051 confirmed cases, including 395 imported cases; a total of 92,818 cases were cured and discharged, and 98,505 cases were confirmed (53). Although the Chinese government has managed the pandemic well, due to the complexity of the virus itself and the severity of the international pandemic, COVID-19 still has a profound impact on the fatigue and mental workload of nurses, especially clinical frontline nursing staff. This may serve as a warning that we still cannot take the impact of COVID-19 lightly, even though the anti-epidemic war has now entered a normalization stage of prevention and control.

### Limitations of the study

This survey has several limitations. First, the data collection was only in one city. Some external factors, such as work culture, social capital, health service, and climate in different regions of China, may also affect the studied health outcomes (54–57). Future studies may include these factors and recruit samples from more regions to generalize the findings. Second, the measurement of sleep, fatigue, and mental workload relied almost entirely on self-reported scales, which are prone to be subjective and may cause a nonobjective result. Hence, to ensure the objectivity and authenticity of the results, some objective measurements (e.g., fatigue testing based on electroencephalogram signs, actigraphy, and Polysomnography) will be considered in future research. Third, because of the cross-sectional study design, this study could not determine causal relationships between influential factors, including personnel environmental satisfaction, fatigue, sleep quality, mental workload, current area of practice and insomnia in past one month. Thus, a more rigorous study design (e.g., cohort study) (58) should be conducted to check the factors related to the sleep, fatigue and mental workload of nurses. In addition, this study only collected data on sleep, fatigue and mental workload for the previous month, which represented a snapshot of the short-term status of the nurses. Therefore, future research should focus on longer periods of time and dynamic data collection to obtain a more comprehensive assessment.

## Conclusion

In summary, clinical first-line nurses demonstrated worse sleep, higher levels of fatigue and lower levels of mental workload during post-epidemic of anti-COVID-19 era. Poor sleep, fatigue and mental workload had a positive and direct relationship with each other. The predictors influencing sleep, fatigue and mental workload were also identified. Moreover, COVID-19 has a greater impact on sleep, fatigue and mental workload among clinical front-line nurses than before and during the pandemic. Nursing managers, policy makers and employers should take sleep, fatigue and mental workload into consideration together rather than solely when assessing nurses’ mental health. Also, some target interventions, such as solving the long sleep latency, improving sleep efficiency, reducing fatigue degree and physical demand, are recommended for more effective management of the sleep, fatigue or mental workload associated risks.

## Data availability statement

The original contributions presented in this study are included in the article/Supplementary material, further inquiries can be directed to the corresponding author/s.

## Ethics statement

The studies involving human participants were reviewed and approved by the Ethics Committee of the First Affiliated Hospital of Army Medical University, PLA (Ref: KY2021062). The patients/participants provided their written informed consent to participate in this study.

## Author contributions

HF, LW, and TC conceived, designed and supervised the study. YiL, KM, XY, NM, and KX collected the data. YaL, JSX, FW, and TL finalized the analysis, designed the study and interpreted the findings. YaL and JSX drafted the manuscript. YaL, FL, YQ, SW, RW, and CY helped revising drafts of the manuscript. FL undertook the main work of revised the manuscript, provided some ideas for revised responses, and helped to write the revised manuscript. YZ and BT helped to organize, reconcile, and interpret raw data when revising the draft. All authors read and approved the final manuscript.

## Footnotes

1
https://www.wjx.cn/
