# Supplementary material for: Factoring and correlation in sleep, fatigue and mental workload of clinical first-line nurses in the post-pandemic era of COVID-19: A multi-center cross-sectional study
Source: Front Psychiatry. 2022 Aug 25;13:963419. doi: 10.3389/fpsyt.2022.963419 (PMC9452657; doi:10.3389/fpsyt.2022.963419)
Supplement: Supplementary file 1 [file Data_Sheet_1.PDF]

ID	mental demand	physical demand	temporal demand	effort	performance	frustration level	mental demand VS physical demand	mental demand VS temporal demand	mental demand VS performance	mental demand VS effort	mental demand VS frustration level
1	69	52	62	44	53	40	mental demand	temporal demand	performance	effort	mental demand
2	52	56	65	51	56	25	physical demand	mental demand	performance	effort	frustration level
3	60	60	70	50	50	30	mental demand	temporal demand	performance	mental demand	frustration level
4	22	63	33	57	59	33	mental demand	temporal demand	performance	mental demand	frustration level
5	64	70	43	44	48	16	physical demand	temporal demand	performance	mental demand	frustration level
6	21	36	57	60	58	62	mental demand	temporal demand	performance	effort	mental demand
7	56	73	85	24	87	14	physical demand	temporal demand	performance	effort	frustration level
8	80	70	47	60	60	59	mental demand	temporal demand	performance	effort	mental demand
9	65	100	84	37	89	37	physical demand	temporal demand	performance	mental demand	frustration level
10	61	72	39	6	52	13	physical demand	temporal demand	performance	effort	frustration level
11	43	89	83	70	66	31	physical demand	temporal demand	performance	mental demand	frustration level
12	50	52	61	48	100	43	physical demand	temporal demand	performance	mental demand	frustration level
13	50	67	56	30	55	48	mental demand	temporal demand	performance	effort	frustration level
14	29	43	34	42	46	40	physical demand	mental demand	mental demand	effort	mental demand
15	60	67	51	6	40	13	physical demand	temporal demand	mental demand	effort	frustration level
16	50	70	28	55	50	34	physical demand	temporal demand	mental demand	mental demand	mental demand
17	25	45	43	40	38	20	mental demand	temporal demand	performance	effort	frustration level
18	99	94	68	8	53	36	physical demand	mental demand	mental demand	mental demand	mental demand
19	64	56	81	65	68	70	physical demand	mental demand	mental demand	mental demand	mental demand
20	60	100	96	21	85	80	physical demand	temporal demand	performance	mental demand	frustration level
21	46	80	74	56	39	59	physical demand	mental demand	performance	mental demand	frustration level
22	47	46	22	48	79	62	mental demand	temporal demand	performance	mental demand	mental demand
23	36	76	81	43	72	23	physical demand	temporal demand	performance	effort	mental demand
24	80	32	40	42	79	11	mental demand	mental demand	performance	mental demand	mental demand
25	80	81	70	61	60	60	physical demand	mental demand	performance	mental demand	mental demand
26	70	80	60	40	60	2	physical demand	temporal demand	performance	effort	frustration level
27	58	70	60	39	50	48	mental demand	temporal demand	mental demand	mental demand	mental demand
28	80	89	79	42	78	37	mental demand	temporal demand	performance	mental demand	mental demand
29	1	32	5	28	5	6	physical demand	temporal demand	performance	mental demand	frustration level
30	50	40	43	47	50	40	physical demand	temporal demand	performance	effort	frustration level
31	60	75	90	55	65	10	physical demand	temporal demand	performance	effort	mental demand
32	81	90	75	29	60	37	physical demand	temporal demand	mental demand	mental demand	mental demand
33	41	78	50	46	50	45	physical demand	temporal demand	performance	mental demand	frustration level
34	72	78	78	46	66	32	physical demand	mental demand	performance	effort	frustration level
35	50	60	60	0	60	45	physical demand	mental demand	performance	effort	frustration level
36	53	39	80	45	70	28	mental demand	temporal demand	performance	mental demand	mental demand

ID	mental demand	physical demand	temporal demand	effort	performance	frustration level	mental demand VS physical demand	mental demand VS temporal demand	mental demand VS performance	mental demand VS effort	mental demand VS frustration level
37	90	87	37	25	31	13	physical demand	temporal demand	performance	effort	mental demand
38	42	55	55	85	50	46	mental demand	temporal demand	performance	mental demand	frustration level
39	51	74	31	28	52	14	physical demand	temporal demand	performance	effort	frustration level
40	63	100	49	10	30	42	mental demand	mental demand	mental demand	mental demand	mental demand
41	55	77	25	30	47	9	physical demand	temporal demand	performance	effort	frustration level
42	63	83	61	60	60	63	mental demand	mental demand	mental demand	effort	mental demand
43	56	57	60	40	50	56	physical demand	temporal demand	mental demand	mental demand	mental demand
44	95	73	69	55	91	73	mental demand	mental demand	mental demand	mental demand	frustration level
45	53	70	45	46	68	47	physical demand	mental demand	mental demand	mental demand	mental demand
46	51	39	40	13	51	11	mental demand	temporal demand	performance	effort	mental demand
47	61	63	100	43	23	21	mental demand	mental demand	performance	effort	frustration level
48	40	50	40	39	40	47	mental demand	mental demand	performance	effort	frustration level
49	24	49	31	24	34	7	physical demand	temporal demand	performance	effort	frustration level
50	87	100	78	24	78	77	physical demand	temporal demand	mental demand	mental demand	mental demand
51	65	72	55	27	52	38	mental demand	mental demand	mental demand	mental demand	mental demand
52	61	60	60	48	46	17	physical demand	mental demand	mental demand	mental demand	mental demand
53	43	13	35	47	34	27	mental demand	temporal demand	performance	effort	frustration level
54	35	61	14	7	31	4	physical demand	temporal demand	performance	effort	mental demand
55	50	48	62	58	79	40	mental demand	temporal demand	performance	effort	frustration level
56	60	60	80	60	89	20	mental demand	temporal demand	performance	mental demand	frustration level
57	60	59	59	60	68	36	mental demand	mental demand	performance	mental demand	frustration level
58	100	100	92	85	84	100	mental demand	temporal demand	performance	mental demand	frustration level
59	41	61	57	42	50	41	physical demand	temporal demand	performance	effort	frustration level
60	65	53	53	55	63	73	mental demand	temporal demand	performance	mental demand	frustration level
61	61	81	60	64	60	63	physical demand	temporal demand	performance	effort	mental demand
62	49	56	50	48	48	14	physical demand	temporal demand	performance	mental demand	frustration level
63	60	79	29	43	23	45	physical demand	mental demand	performance	effort	frustration level
64	80	39	84	76	38	11	mental demand	temporal demand	performance	mental demand	mental demand
65	7	6	18	41	62	23	physical demand	temporal demand	performance	effort	frustration level
66	21	24	8	21	13	20	physical demand	temporal demand	performance	effort	mental demand
67	41	80	86	60	56	55	physical demand	temporal demand	performance	effort	frustration level
68	40	45	50	50	65	60	physical demand	temporal demand	performance	mental demand	frustration level
69	64	17	79	23	60	81	mental demand	mental demand	performance	mental demand	mental demand
70	60	80	35	15	60	20	mental demand	mental demand	performance	effort	frustration level
71	60	85	55	45	59	51	mental demand	temporal demand	performance	mental demand	mental demand
72	53	63	87	39	48	53	physical demand	temporal demand	performance	mental demand	frustration level

ID	mental demand	physical demand	temporal demand	effort	performance	frustration level	mental demand VS physical demand	mental demand VS temporal demand	mental demand VS performance	mental demand VS effort	mental demand VS frustration level
73	54	55	32	29	60	17	mental demand	mental demand	performance	mental demand	mental demand
74	81	100	80	75	87	54	physical demand	temporal demand	mental demand	mental demand	mental demand
75	42	63	62	72	60	17	mental demand	mental demand	performance	effort	mental demand
76	53	57	58	55	60	37	physical demand	mental demand	mental demand	mental demand	mental demand
77	68	41	70	44	50	78	mental demand	temporal demand	performance	mental demand	frustration level
78	54	19	44	44	61	97	mental demand	temporal demand	performance	mental demand	mental demand
79	53	60	60	55	50	70	physical demand	temporal demand	performance	effort	frustration level
80	50	52	50	10	51	30	physical demand	mental demand	performance	effort	frustration level
81	90	90	71	57	72	65	mental demand	mental demand	performance	mental demand	frustration level
82	39	100	37	26	37	93	physical demand	temporal demand	performance	effort	frustration level
83	51	55	32	47	46	12	physical demand	mental demand	mental demand	mental demand	mental demand
84	70	100	61	42	80	30	physical demand	temporal demand	performance	effort	mental demand
85	63	81	69	55	63	31	physical demand	temporal demand	performance	effort	mental demand
86	23	29	21	39	54	40	mental demand	mental demand	mental demand	mental demand	mental demand
87	50	52	51	51	51	53	mental demand	temporal demand	performance	effort	frustration level
88	64	66	100	67	68	46	physical demand	temporal demand	performance	effort	frustration level
89	65	67	60	17	73	33	physical demand	mental demand	mental demand	effort	mental demand
90	54	57	43	44	50	16	mental demand	mental demand	mental demand	mental demand	mental demand
91	76	86	59	20	90	55	physical demand	mental demand	performance	effort	frustration level
92	56	29	38	60	33	30	physical demand	mental demand	mental demand	mental demand	frustration level
93	52	73	53	53	34	34	physical demand	temporal demand	performance	effort	frustration level
94	82	88	85	72	72	81	mental demand	mental demand	mental demand	mental demand	mental demand
95	68	86	41	39	77	36	physical demand	temporal demand	performance	effort	frustration level
96	62	81	38	0	36	11	physical demand	temporal demand	performance	mental demand	mental demand
97	60	50	43	20	60	46	mental demand	temporal demand	performance	mental demand	frustration level
98	50	71	50	10	53	49	physical demand	mental demand	performance	effort	frustration level
99	37	85	66	49	55	38	physical demand	mental demand	performance	effort	mental demand
100	39	53	27	81	42	21	physical demand	mental demand	performance	effort	mental demand
101	56	41	40	43	40	59	mental demand	temporal demand	performance	effort	frustration level
102	60	60	40	58	45	60	physical demand	temporal demand	performance	mental demand	mental demand
103	22	38	29	16	32	14	physical demand	temporal demand	performance	effort	frustration level
104	81	71	51	52	80	52	mental demand	temporal demand	mental demand	effort	mental demand
105	50	63	74	49	54	30	mental demand	mental demand	performance	mental demand	mental demand
106	52	48	24	26	54	50	physical demand	temporal demand	performance	mental demand	mental demand
107	78	100	74	41	48	24	physical demand	mental demand	mental demand	mental demand	mental demand
108	35	50	47	11	59	22	mental demand	mental demand	performance	effort	frustration level

ID	mental demand	physical demand	temporal demand	effort	performance	frustration level	mental demand VS physical demand	mental demand VS temporal demand	mental demand VS performance	mental demand VS effort	mental demand VS frustration level
109	79	72	83	93	94	37	mental demand	temporal demand	performance	effort	frustration level
110	22	39	21	41	50	33	mental demand	temporal demand	performance	mental demand	frustration level
111	51	60	58	24	80	15	physical demand	temporal demand	performance	mental demand	frustration level
112	43	50	22	38	47	31	mental demand	mental demand	performance	effort	frustration level
113	78	33	67	68	70	57	mental demand	temporal demand	performance	effort	frustration level
114	41	66	46	49	54	49	physical demand	temporal demand	performance	mental demand	mental demand
115	60	86	47	31	29	24	mental demand	mental demand	performance	mental demand	frustration level
116	26	33	31	56	60	55	mental demand	mental demand	mental demand	mental demand	mental demand
117	77	60	70	48	65	0	physical demand	temporal demand	mental demand	mental demand	mental demand
118	60	90	61	40	61	50	physical demand	mental demand	performance	effort	frustration level
119	63	61	60	85	67	52	mental demand	mental demand	mental demand	mental demand	mental demand
120	39	46	50	48	49	35	physical demand	temporal demand	performance	mental demand	mental demand
121	72	78	73	27	79	34	mental demand	mental demand	performance	effort	frustration level
122	70	90	90	41	61	60	physical demand	mental demand	mental demand	mental demand	frustration level
123	28	50	100	16	41	10	physical demand	temporal demand	performance	effort	frustration level
124	20	59	28	11	17	28	physical demand	temporal demand	mental demand	mental demand	frustration level
125	50	60	40	45	59	60	physical demand	temporal demand	mental demand	mental demand	frustration level
126	61	69	52	41	46	42	physical demand	mental demand	mental demand	mental demand	mental demand
127	36	59	52	36	40	15	physical demand	temporal demand	mental demand	mental demand	mental demand
128	30	80	30	50	40	20	physical demand	temporal demand	performance	mental demand	frustration level
129	51	67	31	35	63	29	physical demand	mental demand	performance	effort	frustration level
130	50	50	50	50	60	13	mental demand	mental demand	mental demand	mental demand	mental demand
131	68	70	72	51	67	56	mental demand	temporal demand	mental demand	mental demand	mental demand
132	79	73	56	28	24	34	physical demand	temporal demand	performance	effort	frustration level
133	40	58	38	58	59	61	physical demand	mental demand	performance	effort	frustration level
134	32	60	59	60	56	52	physical demand	temporal demand	performance	effort	frustration level
135	70	80	78	30	67	62	mental demand	mental demand	mental demand	effort	mental demand
136	43	41	53	48	52	43	mental demand	mental demand	mental demand	mental demand	mental demand
137	34	40	40	39	40	20	mental demand	mental demand	mental demand	mental demand	mental demand
138	76	80	86	60	82	55	mental demand	mental demand	performance	effort	mental demand
139	55	91	67	56	50	40	physical demand	mental demand	performance	effort	mental demand
140	60	64	43	42	55	55	mental demand	temporal demand	performance	mental demand	frustration level
141	46	49	45	31	56	10	physical demand	mental demand	performance	mental demand	mental demand
142	51	51	45	51	49	24	physical demand	temporal demand	performance	effort	frustration level
143	81	91	91	87	91	89	physical demand	temporal demand	performance	effort	frustration level
144	100	100	100	40	85	100	physical demand	mental demand	mental demand	mental demand	frustration level

ID	mental demand	physical demand	temporal demand	effort	performance	frustration level	mental demand VS physical demand	mental demand VS temporal demand	mental demand VS performance	mental demand VS effort	mental demand VS frustration level
145	61	67	50	18	9	11	mental demand	mental demand	mental demand	mental demand	frustration level
146	45	44	40	58	51	37	physical demand	temporal demand	performance	effort	frustration level
147	50	61	52	52	56	51	physical demand	temporal demand	performance	effort	frustration level
148	56	49	42	54	56	28	mental demand	temporal demand	performance	mental demand	mental demand
149	47	45	27	30	22	25	mental demand	mental demand	mental demand	mental demand	mental demand
150	60	37	60	60	49	38	mental demand	temporal demand	performance	mental demand	frustration level
151	64	26	99	36	86	18	mental demand	mental demand	mental demand	mental demand	mental demand
152	82	46	100	26	87	82	mental demand	temporal demand	performance	mental demand	frustration level
153	86	62	63	54	69	54	physical demand	mental demand	performance	effort	mental demand
154	61	38	34	14	33	20	mental demand	mental demand	mental demand	mental demand	mental demand
155	71	85	88	48	91	73	mental demand	temporal demand	mental demand	mental demand	mental demand
156	95	90	96	52	80	80	mental demand	temporal demand	performance	mental demand	mental demand
157	50	50	51	50	49	51	mental demand	mental demand	mental demand	mental demand	mental demand
158	47	48	38	54	52	7	mental demand	mental demand	mental demand	mental demand	mental demand
159	54	97	33	56	38	32	physical demand	temporal demand	performance	mental demand	mental demand
160	39	78	51	51	68	49	mental demand	mental demand	performance	effort	frustration level
161	63	85	71	67	83	50	mental demand	temporal demand	performance	effort	frustration level
162	10	13	10	22	20	18	physical demand	temporal demand	performance	effort	frustration level
163	50	60	60	30	82	43	physical demand	mental demand	performance	mental demand	mental demand
164	46	47	30	47	55	25	mental demand	mental demand	performance	mental demand	frustration level
165	29	95	93	20	67	55	mental demand	mental demand	mental demand	mental demand	mental demand
166	39	62	35	37	52	53	physical demand	mental demand	performance	mental demand	frustration level
167	53	33	31	56	53	48	mental demand	temporal demand	performance	mental demand	mental demand
168	39	78	50	50	82	62	physical demand	temporal demand	performance	effort	frustration level
169	61	50	60	50	49	31	mental demand	temporal demand	performance	effort	frustration level
170	60	70	45	33	48	33	physical demand	temporal demand	mental demand	effort	mental demand
171	36	43	41	46	44	39	mental demand	temporal demand	performance	effort	frustration level
172	69	90	97	61	34	62	physical demand	temporal demand	mental demand	effort	frustration level
173	41	24	100	22	62	60	mental demand	mental demand	performance	mental demand	mental demand
174	51	84	97	46	93	72	physical demand	mental demand	mental demand	effort	mental demand
175	72	52	56	90	85	24	mental demand	mental demand	mental demand	mental demand	frustration level
176	39	59	39	42	56	11	mental demand	mental demand	mental demand	mental demand	mental demand
177	15	52	34	20	52	7	mental demand	temporal demand	performance	mental demand	frustration level
178	36	41	67	41	57	27	physical demand	temporal demand	performance	effort	mental demand
179	11	20	23	2	8	0	physical demand	temporal demand	performance	effort	mental demand
180	41	17	25	47	30	34	physical demand	mental demand	mental demand	mental demand	mental demand

ID	mental demand	physical demand	temporal demand	effort	performance	frustration level	mental demand VS physical demand	mental demand VS temporal demand	mental demand VS performance	mental demand VS effort	mental demand VS frustration level
181	100	94	100	13	95	20	mental demand	mental demand	performance	mental demand	mental demand
182	56	70	81	52	76	24	physical demand	mental demand	performance	effort	mental demand
183	56	52	23	46	36	21	physical demand	temporal demand	performance	mental demand	mental demand
184	37	43	62	37	49	23	physical demand	temporal demand	mental demand	mental demand	mental demand
185	17	50	35	20	35	10	physical demand	temporal demand	performance	effort	frustration level
186	74	76	69	34	18	18	physical demand	mental demand	mental demand	effort	mental demand
187	46	63	75	36	47	66	physical demand	temporal demand	mental demand	effort	frustration level
188	83	91	67	58	60	86	physical demand	mental demand	mental demand	mental demand	frustration level
189	45	75	85	8	61	23	physical demand	temporal demand	mental demand	effort	mental demand
190	88	77	83	53	100	37	mental demand	temporal demand	mental demand	mental demand	mental demand
191	20	70	60	0	0	0	physical demand	temporal demand	mental demand	mental demand	frustration level
192	27	22	29	29	27	26	physical demand	temporal demand	performance	effort	frustration level
193	59	87	78	25	61	43	mental demand	temporal demand	performance	mental demand	mental demand
194	43	49	60	46	56	42	physical demand	temporal demand	mental demand	effort	mental demand
195	49	62	39	45	42	44	mental demand	mental demand	performance	mental demand	mental demand
196	43	90	97	17	72	52	physical demand	temporal demand	mental demand	mental demand	mental demand
197	41	41	45	41	41	61	mental demand	mental demand	mental demand	effort	frustration level
198	97	96	95	36	77	15	physical demand	mental demand	performance	mental demand	mental demand
199	98	15	80	22	59	18	mental demand	temporal demand	performance	effort	mental demand
200	43	69	51	44	44	42	physical demand	temporal demand	performance	effort	frustration level
201	27	44	32	27	36	18	physical demand	temporal demand	performance	effort	mental demand
202	80	50	60	50	50	40	mental demand	mental demand	mental demand	mental demand	mental demand
203	72	64	78	69	71	77	physical demand	mental demand	performance	mental demand	frustration level
204	53	64	55	64	60	69	physical demand	temporal demand	performance	effort	frustration level
205	98	100	94	48	97	49	physical demand	mental demand	mental demand	mental demand	mental demand
206	54	57	55	56	56	38	mental demand	temporal demand	performance	mental demand	mental demand
207	100	86	100	60	80	60	mental demand	mental demand	performance	mental demand	mental demand
208	60	60	81	41	52	27	physical demand	mental demand	performance	mental demand	mental demand
209	78	79	71	91	76	100	mental demand	mental demand	performance	mental demand	mental demand
210	54	51	36	43	62	40	mental demand	mental demand	performance	effort	frustration level
211	40	40	40	40	40	50	mental demand	temporal demand	performance	effort	frustration level
212	84	77	78	31	76	34	mental demand	mental demand	mental demand	mental demand	mental demand
213	81	88	88	12	53	5	physical demand	temporal demand	performance	effort	frustration level
214	84	100	60	70	50	67	physical demand	temporal demand	mental demand	mental demand	mental demand
215	50	62	50	50	50	50	mental demand	temporal demand	performance	effort	frustration level
216	43	56	31	26	31	3	physical demand	temporal demand	performance	effort	mental demand

ID	mental demand	physical demand	temporal demand	effort	performance	frustration level	mental demand VS physical demand	mental demand VS temporal demand	mental demand VS performance	mental demand VS effort	mental demand VS frustration level
217	84	96	81	31	100	21	physical demand	temporal demand	performance	mental demand	mental demand
218	80	50	50	90	80	40	mental demand	mental demand	performance	mental demand	frustration level
219	40	84	67	27	57	16	mental demand	mental demand	mental demand	mental demand	mental demand
220	79	80	63	55	64	100	mental demand	temporal demand	performance	effort	frustration level
221	61	89	16	23	53	72	physical demand	mental demand	performance	mental demand	frustration level
222	50	60	65	42	47	22	physical demand	temporal demand	performance	effort	mental demand
223	69	68	58	50	48	100	physical demand	mental demand	performance	mental demand	frustration level
224	47	25	18	27	23	10	physical demand	temporal demand	performance	effort	frustration level
225	100	100	100	50	90	60	mental demand	mental demand	performance	mental demand	mental demand
226	50	68	100	92	100	71	mental demand	temporal demand	performance	effort	frustration level
227	81	80	61	60	79	60	physical demand	mental demand	performance	effort	frustration level
228	57	100	48	9	99	79	physical demand	mental demand	performance	effort	frustration level
229	73	73	56	55	52	32	physical demand	temporal demand	mental demand	mental demand	frustration level
230	51	46	61	60	61	41	physical demand	mental demand	performance	mental demand	mental demand
231	52	71	85	52	60	62	physical demand	temporal demand	performance	mental demand	frustration level
232	46	48	52	47	49	29	physical demand	temporal demand	performance	effort	mental demand
233	71	42	32	84	65	23	physical demand	temporal demand	performance	mental demand	frustration level
234	84	62	81	79	95	80	mental demand	temporal demand	performance	effort	frustration level
235	41	62	72	50	48	53	physical demand	temporal demand	performance	mental demand	mental demand
236	29	15	26	28	74	34	physical demand	temporal demand	mental demand	effort	frustration level
237	60	60	30	26	50	30	physical demand	temporal demand	mental demand	effort	mental demand
238	67	90	61	57	71	100	physical demand	temporal demand	mental demand	mental demand	mental demand
239	60	38	80	40	60	40	mental demand	mental demand	mental demand	mental demand	mental demand
240	90	85	75	51	82	27	mental demand	mental demand	mental demand	mental demand	mental demand
241	69	99	96	82	97	82	physical demand	mental demand	mental demand	effort	mental demand
242	48	35	51	51	52	66	physical demand	temporal demand	performance	effort	frustration level
243	61	67	67	45	74	60	mental demand	temporal demand	performance	mental demand	mental demand
244	74	100	39	22	64	95	physical demand	mental demand	performance	mental demand	mental demand
245	41	60	40	60	60	60	physical demand	temporal demand	performance	effort	frustration level
246	61	61	42	59	59	50	physical demand	mental demand	performance	effort	frustration level
247	51	100	78	50	69	65	physical demand	mental demand	performance	effort	mental demand
248	85	90	55	60	60	52	mental demand	temporal demand	performance	mental demand	mental demand
249	42	61	43	39	60	20	mental demand	temporal demand	performance	effort	frustration level
250	59	80	67	54	80	54	physical demand	mental demand	mental demand	mental demand	mental demand
251	51	48	54	56	51	26	mental demand	mental demand	mental demand	mental demand	mental demand
252	66	69	74	51	74	57	physical demand	temporal demand	performance	effort	frustration level

ID	mental demand	physical demand	temporal demand	effort	performance	frustration level	mental demand VS physical demand	mental demand VS temporal demand	mental demand VS performance	mental demand VS effort	mental demand VS frustration level
253	27	60	26	20	50	50	physical demand	mental demand	performance	mental demand	mental demand
254	74	67	97	73	94	84	mental demand	mental demand	mental demand	mental demand	frustration level
255	51	44	56	50	54	57	physical demand	temporal demand	performance	effort	frustration level
256	83	57	70	54	78	53	mental demand	temporal demand	performance	effort	frustration level
257	51	76	63	40	59	14	physical demand	temporal demand	performance	effort	frustration level
258	53	52	58	27	66	26	mental demand	mental demand	mental demand	mental demand	mental demand
259	26	53	30	100	50	0	physical demand	temporal demand	performance	effort	frustration level
260	63	80	56	59	60	44	mental demand	mental demand	performance	effort	frustration level
261	87	85	88	52	86	69	mental demand	temporal demand	performance	mental demand	frustration level
262	83	100	100	42	100	61	mental demand	mental demand	performance	mental demand	frustration level
263	67	68	45	24	64	5	physical demand	temporal demand	performance	effort	frustration level
264	50	80	40	50	50	20	mental demand	mental demand	mental demand	mental demand	mental demand
265	90	92	100	80	90	90	physical demand	temporal demand	performance	effort	frustration level
266	50	63	31	71	66	24	physical demand	mental demand	mental demand	effort	frustration level
267	56	81	82	18	82	36	mental demand	temporal demand	performance	effort	frustration level
268	41	42	40	45	61	41	mental demand	mental demand	mental demand	mental demand	mental demand
269	71	100	91	20	100	31	physical demand	temporal demand	mental demand	mental demand	mental demand
270	52	79	83	52	66	81	physical demand	temporal demand	performance	effort	mental demand
271	73	79	100	61	100	93	physical demand	temporal demand	performance	effort	frustration level
272	85	45	48	57	90	60	mental demand	temporal demand	performance	mental demand	mental demand
273	40	60	80	26	42	17	physical demand	temporal demand	performance	effort	frustration level
274	80	80	62	38	57	57	physical demand	temporal demand	performance	effort	frustration level
275	100	100	100	60	88	100	mental demand	mental demand	mental demand	mental demand	frustration level
276	49	52	68	52	89	50	physical demand	temporal demand	performance	effort	frustration level
277	83	100	81	93	94	46	physical demand	mental demand	performance	effort	mental demand
278	90	100	100	72	98	50	physical demand	mental demand	mental demand	effort	frustration level
279	100	100	100	60	80	100	mental demand	temporal demand	performance	effort	frustration level
280	100	100	88	26	100	31	mental demand	mental demand	performance	mental demand	mental demand
281	97	60	97	92	100	94	mental demand	temporal demand	performance	mental demand	frustration level
282	60	52	60	58	71	58	mental demand	temporal demand	performance	mental demand	mental demand
283	78	96	100	100	100	81	physical demand	mental demand	mental demand	mental demand	mental demand
284	31	52	11	34	31	28	physical demand	mental demand	performance	mental demand	frustration level
285	65	70	72	79	71	74	physical demand	mental demand	performance	mental demand	mental demand
286	52	83	53	53	52	62	physical demand	mental demand	mental demand	mental demand	mental demand
287	63	68	81	59	69	58	physical demand	mental demand	mental demand	mental demand	mental demand
288	70	77	72	23	53	28	mental demand	temporal demand	mental demand	effort	mental demand

ID	mental demand	physical demand	temporal demand	effort	performance	frustration level	mental demand VS physical demand	mental demand VS temporal demand	mental demand VS performance	mental demand VS effort	mental demand VS frustration level
289	74	100	65	43	64	98	mental demand	mental demand	performance	effort	mental demand
290	57	84	70	44	57	65	physical demand	temporal demand	performance	effort	frustration level
291	87	100	100	84	100	99	physical demand	temporal demand	performance	mental demand	frustration level
292	72	85	82	77	87	14	physical demand	temporal demand	performance	mental demand	frustration level
293	90	90	81	63	79	81	mental demand	mental demand	performance	mental demand	frustration level
294	58	62	42	38	60	12	physical demand	temporal demand	performance	effort	mental demand
295	68	56	22	46	88	30	mental demand	mental demand	performance	effort	frustration level
296	80	100	82	31	78	60	physical demand	mental demand	performance	effort	frustration level
297	77	87	80	80	64	46	physical demand	temporal demand	mental demand	mental demand	mental demand
298	19	40	20	20	20	20	physical demand	temporal demand	performance	effort	frustration level
299	61	78	49	59	47	24	physical demand	temporal demand	mental demand	mental demand	mental demand
300	90	63	40	50	50	25	mental demand	temporal demand	performance	mental demand	frustration level
301	35	71	68	97	76	76	mental demand	temporal demand	performance	mental demand	frustration level
302	50	68	48	52	51	47	physical demand	temporal demand	mental demand	mental demand	frustration level
303	81	71	61	27	61	64	physical demand	temporal demand	performance	effort	frustration level
304	20	24	33	39	20	38	mental demand	temporal demand	performance	effort	frustration level
305	71	77	65	50	59	64	physical demand	mental demand	mental demand	mental demand	mental demand
306	74	58	58	37	46	21	physical demand	mental demand	performance	effort	frustration level
307	82	99	99	61	99	100	mental demand	mental demand	mental demand	mental demand	mental demand
308	61	65	63	65	60	18	mental demand	temporal demand	performance	effort	mental demand
309	65	71	74	48	72	24	mental demand	temporal demand	performance	effort	frustration level
310	42	50	72	52	58	21	mental demand	temporal demand	mental demand	effort	mental demand
311	91	97	60	65	74	58	physical demand	temporal demand	performance	effort	frustration level
312	62	88	40	58	93	3	physical demand	temporal demand	performance	effort	frustration level
313	71	80	80	95	85	60	physical demand	temporal demand	performance	mental demand	frustration level
314	72	83	78	53	76	63	mental demand	mental demand	performance	mental demand	frustration level
315	53	77	62	60	78	67	mental demand	mental demand	performance	mental demand	frustration level
316	55	50	57	30	52	30	physical demand	temporal demand	performance	effort	frustration level
317	65	60	41	43	62	58	mental demand	temporal demand	performance	effort	frustration level
318	60	81	69	49	52	52	physical demand	temporal demand	performance	effort	frustration level
319	60	84	81	51	61	74	physical demand	temporal demand	performance	effort	frustration level
320	81	80	81	61	80	23	mental demand	temporal demand	mental demand	effort	mental demand
321	59	78	100	31	100	25	physical demand	temporal demand	performance	mental demand	frustration level
322	82	92	96	30	68	29	mental demand	temporal demand	performance	effort	frustration level
323	40	65	50	65	83	21	physical demand	temporal demand	performance	effort	frustration level
324	100	27	22	4	8	9	physical demand	temporal demand	performance	effort	mental demand

ID	mental demand	physical demand	temporal demand	effort	performance	frustration level	mental demand VS physical demand	mental demand VS temporal demand	mental demand VS performance	mental demand VS effort	mental demand VS frustration level
325	51	78	43	20	46	36	physical demand	temporal demand	performance	effort	frustration level
326	36	54	39	42	11	12	mental demand	mental demand	performance	mental demand	mental demand
327	50	90	81	81	79	83	physical demand	temporal demand	performance	mental demand	mental demand
328	58	51	39	34	45	9	physical demand	temporal demand	performance	mental demand	mental demand
329	80	90	80	30	80	50	mental demand	mental demand	mental demand	mental demand	mental demand
330	42	100	73	27	62	44	physical demand	temporal demand	performance	effort	frustration level
331	48	94	83	27	72	9	physical demand	mental demand	performance	effort	mental demand
332	45	58	62	89	80	66	mental demand	mental demand	mental demand	mental demand	mental demand
333	27	45	38	40	37	24	mental demand	mental demand	performance	effort	mental demand
334	90	80	60	90	90	20	mental demand	mental demand	performance	effort	frustration level
335	97	100	100	97	100	90	mental demand	mental demand	mental demand	mental demand	mental demand
336	72	67	72	35	50	29	mental demand	temporal demand	performance	effort	frustration level
337	58	100	83	39	51	77	physical demand	temporal demand	performance	mental demand	frustration level
338	47	27	43	25	56	16	mental demand	temporal demand	performance	effort	frustration level
339	20	40	43	42	37	46	mental demand	temporal demand	mental demand	mental demand	frustration level
340	60	59	6	5	39	1	mental demand	temporal demand	performance	effort	mental demand
341	58	79	98	57	83	79	physical demand	temporal demand	performance	effort	mental demand
342	55	57	53	53	54	51	physical demand	temporal demand	performance	effort	frustration level
343	61	88	41	21	60	0	physical demand	temporal demand	performance	effort	mental demand
344	63	90	40	100	61	0	physical demand	temporal demand	performance	effort	mental demand
345	45	83	40	22	67	11	physical demand	temporal demand	performance	effort	frustration level
346	82	100	100	45	100	51	mental demand	temporal demand	performance	mental demand	frustration level
347	80	80	75	21	80	50	mental demand	temporal demand	performance	effort	frustration level
348	59	77	66	22	58	51	mental demand	mental demand	performance	effort	frustration level
349	82	74	56	65	79	46	mental demand	mental demand	performance	mental demand	mental demand
350	100	85	89	77	82	86	mental demand	temporal demand	mental demand	mental demand	mental demand
351	46	85	80	34	80	79	physical demand	temporal demand	mental demand	mental demand	mental demand
352	59	59	70	72	67	69	mental demand	temporal demand	performance	mental demand	mental demand
353	79	73	73	38	69	26	physical demand	temporal demand	mental demand	effort	frustration level
354	41	60	40	23	60	0	physical demand	temporal demand	performance	effort	mental demand
355	75	100	63	15	72	34	physical demand	mental demand	mental demand	mental demand	mental demand
356	80	62	60	60	80	83	physical demand	mental demand	mental demand	mental demand	mental demand
357	100	100	43	43	100	20	physical demand	temporal demand	performance	mental demand	frustration level
358	41	90	34	46	50	33	mental demand	temporal demand	mental demand	effort	frustration level
359	45	46	27	25	25	31	physical demand	temporal demand	performance	effort	frustration level
360	41	43	21	18	23	0	mental demand	temporal demand	performance	effort	frustration level

ID	mental demand	physical demand	temporal demand	effort	performance	frustration level	mental demand VS physical demand	mental demand VS temporal demand	mental demand VS performance	mental demand VS effort	mental demand VS frustration level
361	80	100	100	40	100	100	mental demand	temporal demand	performance	mental demand	frustration level
362	83	78	40	18	69	34	physical demand	temporal demand	performance	effort	frustration level
363	53	57	53	31	46	46	physical demand	temporal demand	performance	mental demand	frustration level
364	10	14	11	10	29	17	mental demand	mental demand	mental demand	mental demand	mental demand
365	73	71	52	44	81	50	mental demand	mental demand	performance	effort	mental demand
366	60	61	60	6	58	29	mental demand	mental demand	mental demand	effort	frustration level
367	53	77	84	43	65	60	physical demand	temporal demand	performance	mental demand	frustration level
368	46	49	46	45	47	46	physical demand	temporal demand	mental demand	mental demand	mental demand
369	80	70	80	50	80	65	mental demand	mental demand	performance	mental demand	frustration level
370	78	89	68	47	45	25	physical demand	temporal demand	mental demand	mental demand	mental demand
371	50	43	47	50	52	28	mental demand	temporal demand	performance	effort	frustration level
372	60	57	54	65	60	59	physical demand	temporal demand	performance	mental demand	mental demand
373	72	64	81	6	69	0	mental demand	mental demand	performance	effort	mental demand
374	62	61	49	61	48	51	mental demand	mental demand	mental demand	mental demand	frustration level
375	99	100	58	49	60	50	physical demand	temporal demand	performance	mental demand	frustration level
376	40	59	44	52	69	79	mental demand	mental demand	performance	effort	frustration level
377	80	80	100	51	81	75	physical demand	mental demand	mental demand	mental demand	mental demand
378	100	100	100	12	74	52	physical demand	temporal demand	performance	effort	frustration level
379	100	100	100	59	100	82	physical demand	mental demand	mental demand	mental demand	mental demand
380	59	78	40	39	63	26	mental demand	mental demand	performance	effort	mental demand
381	80	100	100	100	100	100	physical demand	mental demand	performance	mental demand	frustration level
382	60	80	40	30	39	20	physical demand	temporal demand	performance	mental demand	frustration level
383	55	57	54	52	36	22	physical demand	temporal demand	performance	effort	frustration level
384	40	77	0	0	21	0	physical demand	temporal demand	performance	effort	mental demand
385	25	22	22	21	10	25	mental demand	mental demand	performance	effort	frustration level
386	81	45	100	46	100	80	physical demand	temporal demand	performance	mental demand	mental demand
387	61	62	64	45	79	59	mental demand	mental demand	performance	mental demand	frustration level
388	12	56	23	20	24	17	physical demand	mental demand	performance	effort	frustration level
389	59	60	95	2	90	20	mental demand	temporal demand	mental demand	mental demand	mental demand
390	80	95	74	95	91	49	physical demand	mental demand	performance	mental demand	mental demand
391	45	92	57	47	37	14	physical demand	temporal demand	performance	effort	frustration level
392	73	76	69	42	71	70	mental demand	mental demand	performance	mental demand	mental demand
393	39	49	14	32	51	22	physical demand	mental demand	mental demand	mental demand	mental demand
394	61	48	30	46	73	45	mental demand	temporal demand	mental demand	effort	frustration level
395	80	48	68	25	53	31	physical demand	mental demand	performance	effort	frustration level
396	40	20	21	22	41	20	physical demand	temporal demand	performance	mental demand	mental demand

ID	mental demand	physical demand	temporal demand	effort	performance	frustration level	mental demand VS physical demand	mental demand VS temporal demand	mental demand VS performance	mental demand VS effort	mental demand VS frustration level
397	39	40	51	51	51	29	physical demand	temporal demand	performance	effort	frustration level
398	100	100	100	60	84	87	mental demand	mental demand	mental demand	mental demand	mental demand
399	0	29	37	25	22	11	physical demand	temporal demand	performance	effort	frustration level
400	78	70	68	30	70	23	physical demand	temporal demand	mental demand	effort	mental demand
401	80	80	60	20	80	60	physical demand	temporal demand	performance	mental demand	frustration level
402	54	81	60	72	76	31	physical demand	temporal demand	performance	effort	frustration level
403	50	73	52	50	55	54	physical demand	temporal demand	performance	effort	frustration level
404	21	55	55	0	23	5	mental demand	mental demand	mental demand	effort	frustration level
405	31	25	28	46	26	27	physical demand	temporal demand	performance	mental demand	frustration level
406	64	45	45	47	48	38	mental demand	temporal demand	performance	effort	frustration level
407	48	65	0	49	32	52	physical demand	temporal demand	performance	effort	frustration level
408	66	63	63	41	65	61	physical demand	temporal demand	performance	effort	frustration level
409	59	79	95	28	82	84	mental demand	temporal demand	performance	effort	frustration level
410	60	61	40	26	34	10	physical demand	temporal demand	performance	effort	frustration level
411	85	70	60	60	85	75	physical demand	mental demand	performance	mental demand	mental demand
412	66	63	87	13	77	21	physical demand	temporal demand	performance	effort	frustration level
413	36	52	35	15	19	0	physical demand	temporal demand	mental demand	effort	mental demand
414	60	81	24	42	39	84	physical demand	temporal demand	performance	effort	frustration level
415	81	99	85	49	95	82	physical demand	temporal demand	performance	mental demand	mental demand
416	82	60	79	34	76	26	physical demand	temporal demand	performance	mental demand	frustration level
417	85	60	60	57	91	62	mental demand	mental demand	performance	mental demand	frustration level
418	61	60	59	64	59	45	physical demand	mental demand	performance	effort	frustration level
419	60	77	51	50	52	31	physical demand	temporal demand	performance	mental demand	frustration level
420	77	81	82	52	85	78	physical demand	mental demand	performance	mental demand	frustration level
421	53	46	16	20	48	49	physical demand	temporal demand	performance	mental demand	frustration level
422	78	52	50	51	51	43	physical demand	temporal demand	performance	mental demand	mental demand
423	17	26	26	10	13	13	physical demand	temporal demand	performance	effort	mental demand
424	18	21	66	48	67	14	physical demand	temporal demand	performance	effort	mental demand
425	36	75	46	44	65	40	physical demand	temporal demand	performance	effort	mental demand
426	100	100	100	30	100	15	physical demand	mental demand	mental demand	mental demand	mental demand
427	73	70	33	29	33	19	physical demand	mental demand	performance	effort	mental demand
428	68	64	72	59	65	14	physical demand	temporal demand	performance	effort	mental demand
429	90	81	60	60	100	20	mental demand	mental demand	performance	mental demand	frustration level
430	58	94	64	86	59	21	physical demand	temporal demand	performance	effort	frustration level
431	82	58	60	90	100	38	mental demand	temporal demand	performance	effort	frustration level
432	85	62	20	53	68	62	mental demand	mental demand	performance	effort	frustration level

ID	mental demand	physical demand	temporal demand	effort	performance	frustration level	mental demand VS physical demand	mental demand VS temporal demand	mental demand VS performance	mental demand VS effort	mental demand VS frustration level
433	64	78	94	42	64	28	physical demand	mental demand	performance	effort	mental demand
434	67	100	27	17	100	70	physical demand	temporal demand	performance	effort	frustration level
435	57	76	56	36	60	79	physical demand	temporal demand	performance	effort	frustration level
436	63	41	40	49	49	61	mental demand	mental demand	performance	mental demand	frustration level
437	59	55	55	19	58	50	physical demand	temporal demand	mental demand	mental demand	mental demand
438	62	38	62	36	65	62	mental demand	mental demand	performance	effort	mental demand
439	69	96	64	50	47	32	mental demand	mental demand	mental demand	mental demand	frustration level
440	56	52	57	38	37	11	physical demand	temporal demand	performance	effort	frustration level
441	65	55	54	12	24	10	physical demand	temporal demand	performance	effort	frustration level
442	77	85	89	29	85	38	physical demand	mental demand	mental demand	mental demand	mental demand
443	43	57	41	53	57	53	physical demand	temporal demand	performance	effort	mental demand
444	88	100	100	55	100	16	physical demand	temporal demand	performance	mental demand	mental demand
445	60	51	42	29	28	26	mental demand	temporal demand	performance	effort	frustration level
446	0	79	28	10	28	10	mental demand	temporal demand	performance	effort	frustration level
447	78	87	86	43	85	93	physical demand	temporal demand	performance	effort	mental demand
448	51	100	58	50	61	33	physical demand	temporal demand	performance	effort	mental demand
449	60	80	63	51	60	50	physical demand	temporal demand	performance	effort	frustration level
450	62	70	73	38	63	45	mental demand	mental demand	mental demand	effort	frustration level
451	79	68	47	38	64	16	physical demand	mental demand	performance	effort	frustration level
452	68	100	76	45	85	59	physical demand	temporal demand	performance	effort	mental demand
453	80	80	51	24	80	7	mental demand	temporal demand	performance	effort	frustration level
454	57	81	57	53	80	57	mental demand	temporal demand	performance	effort	mental demand
455	79	58	61	18	62	58	physical demand	temporal demand	mental demand	mental demand	mental demand
456	30	46	30	23	34	10	physical demand	temporal demand	performance	effort	frustration level
457	81	75	61	10	41	20	physical demand	temporal demand	performance	effort	frustration level
458	41	100	100	60	59	80	physical demand	temporal demand	performance	effort	frustration level
459	100	100	93	45	94	34	mental demand	temporal demand	performance	mental demand	frustration level
460	30	58	67	51	52	88	physical demand	temporal demand	performance	effort	frustration level
461	100	100	100	57	77	53	physical demand	temporal demand	performance	effort	mental demand
462	57	51	58	47	57	49	physical demand	mental demand	mental demand	effort	mental demand
463	39	31	48	51	53	12	physical demand	temporal demand	mental demand	mental demand	frustration level
464	84	72	96	53	77	59	mental demand	mental demand	performance	mental demand	mental demand
465	86	79	89	56	81	55	mental demand	temporal demand	performance	effort	frustration level
466	80	86	77	57	75	78	mental demand	mental demand	performance	mental demand	frustration level
467	50	75	40	50	59	18	physical demand	temporal demand	mental demand	effort	frustration level
468	39	58	36	21	59	57	physical demand	temporal demand	performance	mental demand	mental demand

ID	mental demand	physical demand	temporal demand	effort	performance	frustration level	mental demand VS physical demand	mental demand VS temporal demand	mental demand VS performance	mental demand VS effort	mental demand VS frustration level
469	92	92	71	40	50	32	physical demand	temporal demand	mental demand	mental demand	mental demand
470	59	52	40	30	51	22	physical demand	temporal demand	performance	effort	mental demand
471	48	50	37	37	51	24	physical demand	temporal demand	mental demand	mental demand	mental demand
472	100	100	100	61	100	100	physical demand	temporal demand	performance	effort	mental demand
473	56	71	59	30	59	28	physical demand	temporal demand	mental demand	mental demand	mental demand
474	50	50	50	45	92	40	physical demand	temporal demand	performance	effort	frustration level
475	57	76	85	54	72	28	physical demand	temporal demand	performance	effort	frustration level
476	49	85	78	85	80	80	physical demand	temporal demand	performance	effort	frustration level
477	53	61	68	57	61	62	physical demand	temporal demand	performance	effort	frustration level
478	81	82	65	57	76	57	mental demand	mental demand	performance	effort	frustration level
479	59	40	80	25	62	60	mental demand	mental demand	performance	mental demand	frustration level
480	60	100	30	30	74	60	physical demand	temporal demand	performance	effort	frustration level
481	23	44	34	52	13	15	physical demand	temporal demand	performance	effort	mental demand
482	60	43	50	52	60	46	physical demand	temporal demand	performance	effort	frustration level
483	82	77	38	29	67	36	physical demand	mental demand	performance	effort	frustration level
484	55	61	56	56	55	68	physical demand	temporal demand	performance	effort	frustration level
485	60	80	100	60	100	100	physical demand	temporal demand	mental demand	effort	frustration level
486	21	50	40	27	39	14	physical demand	temporal demand	performance	effort	mental demand
487	46	90	65	20	61	0	physical demand	temporal demand	mental demand	effort	mental demand
488	41	79	59	40	21	21	physical demand	temporal demand	performance	effort	frustration level
489	62	100	73	75	79	64	physical demand	temporal demand	performance	effort	frustration level
490	74	94	77	49	89	70	physical demand	temporal demand	performance	effort	frustration level
491	53	28	20	50	60	40	mental demand	mental demand	performance	effort	mental demand
492	22	32	37	26	19	15	physical demand	temporal demand	performance	effort	frustration level
493	45	76	50	33	51	21	physical demand	temporal demand	performance	effort	frustration level
494	29	51	33	52	51	52	physical demand	temporal demand	performance	effort	mental demand
495	54	25	21	48	29	15	mental demand	temporal demand	performance	mental demand	frustration level
496	88	78	51	22	96	73	mental demand	temporal demand	performance	effort	mental demand
497	100	100	100	100	100	84	mental demand	temporal demand	performance	mental demand	mental demand
498	56	80	54	60	71	64	mental demand	temporal demand	mental demand	mental demand	mental demand
499	82	86	68	62	87	47	mental demand	mental demand	performance	mental demand	frustration level
500	67	87	79	60	68	89	physical demand	mental demand	performance	effort	frustration level
501	100	100	100	22	81	100	mental demand	mental demand	mental demand	mental demand	mental demand
502	24	20	16	29	31	0	physical demand	temporal demand	performance	effort	frustration level
503	51	79	75	15	74	13	physical demand	temporal demand	performance	mental demand	mental demand
504	20	20	100	38	26	79	mental demand	mental demand	mental demand	mental demand	mental demand

ID	mental demand	physical demand	temporal demand	effort	performance	frustration level	mental demand VS physical demand	mental demand VS temporal demand	mental demand VS performance	mental demand VS effort	mental demand VS frustration level
505	80	79	83	56	74	5	physical demand	mental demand	performance	effort	mental demand
506	34	52	35	48	52	54	physical demand	mental demand	performance	effort	mental demand
507	66	70	60	35	73	18	mental demand	temporal demand	performance	effort	frustration level
508	48	65	42	3	50	26	physical demand	temporal demand	performance	effort	frustration level
509	84	81	78	56	81	58	mental demand	mental demand	mental demand	mental demand	mental demand
510	93	100	98	18	100	60	physical demand	temporal demand	performance	effort	mental demand
511	44	100	84	100	63	69	physical demand	temporal demand	performance	effort	mental demand
512	38	63	66	59	62	60	mental demand	mental demand	performance	effort	frustration level
513	78	92	50	21	60	41	physical demand	mental demand	performance	effort	mental demand
514	56	98	39	7	92	10	physical demand	temporal demand	performance	effort	frustration level
515	54	51	53	39	69	47	mental demand	mental demand	performance	effort	frustration level
516	42	94	46	50	47	47	physical demand	temporal demand	performance	mental demand	mental demand
517	75	80	61	81	66	59	physical demand	temporal demand	mental demand	effort	frustration level
518	74	87	65	30	75	52	mental demand	temporal demand	performance	mental demand	frustration level
519	62	66	43	41	40	38	physical demand	mental demand	performance	mental demand	mental demand
520	59	58	44	53	53	50	mental demand	mental demand	mental demand	mental demand	mental demand
521	54	53	48	41	47	26	physical demand	mental demand	performance	effort	frustration level
522	77	77	75	32	65	40	physical demand	temporal demand	performance	mental demand	mental demand
523	60	79	77	44	47	41	mental demand	mental demand	mental demand	mental demand	mental demand
524	83	77	86	61	57	62	physical demand	temporal demand	performance	effort	mental demand
525	13	12	28	7	25	8	mental demand	temporal demand	performance	effort	mental demand
526	54	56	54	25	54	21	physical demand	temporal demand	performance	effort	frustration level
527	59	87	100	56	73	47	physical demand	temporal demand	performance	effort	frustration level
528	26	39	36	41	28	38	physical demand	temporal demand	performance	effort	frustration level
529	87	100	78	40	100	61	physical demand	mental demand	performance	effort	frustration level
530	54	11	51	19	33	24	physical demand	mental demand	mental demand	mental demand	mental demand
531	79	100	38	40	63	5	mental demand	mental demand	performance	mental demand	mental demand
532	70	74	78	61	93	41	mental demand	mental demand	performance	effort	mental demand
533	50	19	47	48	55	55	physical demand	mental demand	performance	effort	frustration level
534	45	82	72	39	68	55	mental demand	mental demand	performance	effort	frustration level
535	39	49	52	38	42	11	physical demand	temporal demand	performance	effort	frustration level
536	61	93	70	58	45	63	physical demand	mental demand	performance	effort	mental demand
537	0	0	0	0	13	12	mental demand	mental demand	performance	mental demand	mental demand
538	56	50	40	40	50	55	physical demand	temporal demand	performance	effort	frustration level
539	80	100	70	40	80	61	physical demand	mental demand	performance	mental demand	frustration level
540	64	62	61	65	74	19	mental demand	temporal demand	mental demand	effort	mental demand

ID	mental demand	physical demand	temporal demand	effort	performance	frustration level	mental demand VS physical demand	mental demand VS temporal demand	mental demand VS performance	mental demand VS effort	mental demand VS frustration level
541	82	60	61	58	95	31	mental demand	mental demand	mental demand	mental demand	mental demand
542	56	36	61	34	55	39	physical demand	mental demand	performance	effort	frustration level
543	86	100	100	36	80	75	physical demand	temporal demand	mental demand	mental demand	frustration level
544	44	55	81	60	42	60	physical demand	temporal demand	mental demand	mental demand	mental demand
545	63	26	67	54	48	44	mental demand	mental demand	performance	effort	mental demand
546	25	66	61	56	42	55	physical demand	temporal demand	performance	effort	frustration level
547	51	78	18	22	66	7	physical demand	mental demand	performance	mental demand	mental demand
548	83	44	77	85	96	6	mental demand	mental demand	mental demand	effort	mental demand
549	43	41	27	3	100	51	mental demand	mental demand	performance	mental demand	mental demand
550	56	52	53	29	55	27	mental demand	mental demand	performance	effort	frustration level
551	71	86	79	59	68	55	physical demand	mental demand	performance	effort	mental demand
552	72	74	50	50	38	23	physical demand	temporal demand	mental demand	mental demand	frustration level
553	55	49	60	50	56	31	mental demand	temporal demand	performance	mental demand	mental demand
554	62	72	86	80	81	42	mental demand	temporal demand	performance	effort	mental demand
555	60	83	55	36	53	96	physical demand	mental demand	performance	effort	frustration level
556	66	59	66	56	59	20	mental demand	mental demand	mental demand	mental demand	mental demand
557	68	95	67	27	90	94	physical demand	temporal demand	performance	effort	frustration level
558	56	15	73	46	44	20	mental demand	temporal demand	performance	mental demand	mental demand
559	60	70	60	51	56	40	physical demand	mental demand	mental demand	effort	mental demand
560	55	66	43	50	48	12	physical demand	temporal demand	performance	effort	frustration level
561	100	100	100	53	97	94	physical demand	temporal demand	mental demand	mental demand	frustration level
562	61	79	41	60	60	77	physical demand	temporal demand	performance	effort	frustration level
563	50	55	27	48	33	19	physical demand	mental demand	performance	effort	frustration level
564	80	100	100	58	80	100	physical demand	temporal demand	performance	mental demand	frustration level
565	60	44	45	7	49	10	mental demand	temporal demand	performance	effort	frustration level
566	41	45	29	24	15	18	physical demand	temporal demand	performance	effort	mental demand
567	82	70	57	56	55	74	mental demand	mental demand	performance	mental demand	frustration level
568	40	100	87	50	60	67	physical demand	temporal demand	performance	effort	frustration level
569	62	20	81	75	100	56	mental demand	temporal demand	performance	effort	mental demand
570	78	100	67	62	67	65	physical demand	temporal demand	performance	effort	frustration level
571	60	80	85	60	100	83	physical demand	temporal demand	performance	effort	frustration level
572	62	81	50	22	50	1	physical demand	temporal demand	performance	mental demand	frustration level
573	24	24	23	25	46	14	physical demand	mental demand	performance	effort	mental demand
574	43	48	30	40	50	20	mental demand	mental demand	performance	effort	frustration level
575	53	73	70	50	52	50	physical demand	mental demand	performance	mental demand	frustration level
576	70	60	65	41	70	35	mental demand	temporal demand	performance	effort	frustration level

ID	mental demand	physical demand	temporal demand	effort	performance	frustration level	mental demand VS physical demand	mental demand VS temporal demand	mental demand VS performance	mental demand VS effort	mental demand VS frustration level
577	19	44	30	52	26	23	physical demand	temporal demand	performance	effort	mental demand
578	40	58	44	59	14	45	physical demand	temporal demand	performance	effort	frustration level
579	82	100	100	49	80	48	physical demand	temporal demand	performance	mental demand	frustration level
580	80	88	68	56	60	51	physical demand	mental demand	mental demand	mental demand	frustration level
581	51	72	75	47	77	52	mental demand	mental demand	performance	effort	frustration level
582	49	54	28	31	34	12	mental demand	mental demand	performance	mental demand	frustration level
583	50	49	53	41	48	39	physical demand	mental demand	performance	effort	frustration level
584	47	80	77	55	80	62	physical demand	temporal demand	mental demand	effort	frustration level
585	76	90	97	9	98	90	physical demand	mental demand	performance	effort	frustration level
586	81	86	77	61	70	59	mental demand	temporal demand	mental demand	effort	frustration level
587	20	22	100	21	40	24	mental demand	temporal demand	performance	effort	mental demand
588	100	100	46	11	100	27	physical demand	mental demand	performance	effort	frustration level
589	62	100	89	35	79	66	physical demand	temporal demand	performance	mental demand	frustration level
590	41	78	78	45	71	48	physical demand	mental demand	performance	effort	mental demand
591	48	86	44	22	28	6	physical demand	temporal demand	mental demand	mental demand	mental demand
592	83	76	45	58	85	76	mental demand	mental demand	mental demand	mental demand	mental demand
593	40	80	74	20	56	24	mental demand	temporal demand	performance	mental demand	frustration level
594	25	13	23	40	50	30	physical demand	temporal demand	performance	effort	frustration level
595	61	82	70	50	41	13	physical demand	temporal demand	mental demand	mental demand	mental demand
596	33	70	63	45	68	56	physical demand	mental demand	performance	mental demand	mental demand
597	50	50	50	60	50	50	physical demand	temporal demand	performance	effort	mental demand
598	95	77	83	0	100	36	physical demand	mental demand	mental demand	mental demand	mental demand
599	63	49	54	55	61	66	physical demand	temporal demand	performance	effort	frustration level
600	46	57	49	47	51	50	physical demand	temporal demand	performance	effort	frustration level
601	56	59	48	54	75	51	physical demand	temporal demand	performance	effort	mental demand
602	59	59	79	38	63	51	physical demand	mental demand	performance	mental demand	frustration level
603	100	100	100	100	94	100	physical demand	temporal demand	performance	effort	frustration level
604	79	80	47	79	65	37	mental demand	mental demand	performance	mental demand	frustration level
605	24	83	47	47	100	72	physical demand	temporal demand	mental demand	mental demand	mental demand
606	88	100	100	90	100	40	physical demand	temporal demand	performance	effort	frustration level
607	100	100	80	14	50	28	physical demand	mental demand	performance	mental demand	frustration level
608	50	53	35	10	74	4	physical demand	mental demand	performance	mental demand	mental demand
609	66	90	72	29	68	68	physical demand	temporal demand	mental demand	effort	mental demand
610	59	69	64	60	74	92	mental demand	mental demand	performance	mental demand	frustration level
611	60	60	60	21	37	34	physical demand	temporal demand	performance	effort	frustration level
612	68	64	72	26	52	27	physical demand	temporal demand	performance	mental demand	frustration level

ID	mental demand	physical demand	temporal demand	effort	performance	frustration level	mental demand VS physical demand	mental demand VS temporal demand	mental demand VS performance	mental demand VS effort	mental demand VS frustration level
613	57	51	64	27	66	13	mental demand	temporal demand	performance	effort	frustration level
614	65	64	43	17	16	20	mental demand	mental demand	performance	effort	mental demand
615	81	78	80	61	93	81	physical demand	mental demand	performance	effort	mental demand
616	45	28	9	34	48	61	mental demand	mental demand	performance	effort	frustration level
617	33	42	37	71	60	26	physical demand	temporal demand	performance	effort	frustration level
618	100	100	100	52	80	78	physical demand	temporal demand	performance	effort	frustration level
619	4	5	3	34	26	10	mental demand	mental demand	mental demand	effort	mental demand
620	74	78	79	59	69	29	physical demand	temporal demand	performance	mental demand	frustration level
621	90	80	75	5	43	8	mental demand	temporal demand	performance	effort	frustration level
622	50	77	100	56	62	27	physical demand	temporal demand	mental demand	effort	frustration level
623	100	61	61	22	62	57	physical demand	temporal demand	performance	effort	frustration level
624	60	20	42	40	60	81	mental demand	mental demand	performance	mental demand	frustration level
625	50	56	55	42	59	46	mental demand	mental demand	performance	effort	mental demand
626	44	74	53	26	53	33	physical demand	temporal demand	mental demand	effort	frustration level
627	27	37	28	48	25	41	physical demand	mental demand	mental demand	effort	mental demand
628	63	98	60	40	51	66	physical demand	temporal demand	performance	effort	frustration level
629	48	89	51	49	53	51	physical demand	temporal demand	performance	effort	frustration level
630	75	100	80	48	81	52	physical demand	temporal demand	performance	effort	frustration level
631	14	28	27	31	25	10	physical demand	temporal demand	performance	effort	frustration level
632	53	59	56	53	69	52	mental demand	mental demand	performance	effort	frustration level
633	69	83	60	57	62	47	physical demand	temporal demand	performance	effort	frustration level
634	77	91	77	65	75	29	mental demand	temporal demand	mental demand	mental demand	mental demand
635	45	50	40	40	27	18	mental demand	mental demand	performance	effort	mental demand
636	54	33	79	24	63	49	physical demand	temporal demand	performance	effort	frustration level
637	54	55	65	36	32	44	physical demand	temporal demand	mental demand	effort	mental demand
638	51	52	100	19	25	5	mental demand	mental demand	mental demand	mental demand	mental demand
639	46	63	45	50	61	45	physical demand	temporal demand	mental demand	mental demand	frustration level
640	57	64	56	24	65	37	mental demand	mental demand	performance	effort	mental demand
641	61	62	50	35	50	25	physical demand	temporal demand	mental demand	effort	mental demand
642	51	61	59	54	56	56	mental demand	mental demand	mental demand	mental demand	frustration level
643	63	46	61	80	100	46	mental demand	mental demand	performance	effort	mental demand
644	40	36	19	49	30	14	physical demand	mental demand	performance	effort	mental demand
645	28	69	37	50	50	13	physical demand	mental demand	performance	effort	frustration level
646	53	100	77	60	100	49	physical demand	temporal demand	performance	mental demand	mental demand
647	73	15	88	41	74	40	mental demand	temporal demand	performance	effort	frustration level
648	97	100	100	14	89	10	physical demand	temporal demand	performance	effort	frustration level

ID	mental demand	physical demand	temporal demand	effort	performance	frustration level	mental demand VS physical demand	mental demand VS temporal demand	mental demand VS performance	mental demand VS effort	mental demand VS frustration level
649	49	45	60	50	41	21	mental demand	temporal demand	performance	effort	frustration level
650	72	71	55	51	74	71	mental demand	mental demand	mental demand	mental demand	frustration level
651	60	70	50	40	90	10	physical demand	temporal demand	performance	effort	mental demand
652	70	69	52	40	58	74	mental demand	mental demand	performance	effort	frustration level
653	95	100	38	38	68	28	mental demand	temporal demand	performance	mental demand	mental demand
654	58	86	67	51	58	52	physical demand	mental demand	performance	effort	mental demand
655	56	56	59	59	61	67	mental demand	mental demand	performance	effort	mental demand
656	53	51	70	47	33	12	physical demand	mental demand	mental demand	mental demand	frustration level
657	51	58	60	57	59	16	physical demand	mental demand	performance	mental demand	frustration level
658	60	60	10	30	66	30	physical demand	mental demand	performance	effort	frustration level
659	45	68	71	51	50	23	physical demand	temporal demand	performance	effort	mental demand
660	23	34	12	11	19	28	physical demand	temporal demand	performance	effort	frustration level
661	63	63	42	41	42	19	physical demand	temporal demand	performance	effort	frustration level
662	51	51	42	49	61	43	physical demand	temporal demand	performance	effort	frustration level
663	84	69	60	16	53	12	mental demand	temporal demand	performance	mental demand	mental demand
664	28	27	31	40	43	19	physical demand	mental demand	performance	effort	mental demand
665	44	33	66	52	54	13	physical demand	temporal demand	performance	effort	mental demand
666	38	16	20	10	50	0	mental demand	mental demand	performance	mental demand	frustration level
667	65	70	64	50	59	32	physical demand	temporal demand	performance	mental demand	mental demand
668	34	100	100	12	47	54	physical demand	temporal demand	performance	effort	frustration level
669	71	79	78	97	83	23	physical demand	temporal demand	performance	mental demand	mental demand
670	79	24	37	20	55	21	physical demand	mental demand	performance	mental demand	frustration level
671	60	60	40	40	50	30	mental demand	temporal demand	performance	effort	mental demand
672	60	100	75	84	81	80	physical demand	temporal demand	performance	effort	frustration level
673	50	55	33	51	29	10	physical demand	temporal demand	performance	effort	frustration level
674	50	80	75	10	80	60	physical demand	temporal demand	mental demand	effort	mental demand
675	61	100	100	15	92	60	physical demand	mental demand	performance	mental demand	frustration level
676	60	82	62	49	73	28	physical demand	mental demand	performance	mental demand	mental demand
677	29	20	20	29	27	19	mental demand	mental demand	performance	mental demand	frustration level
678	67	80	61	23	32	47	physical demand	mental demand	performance	effort	frustration level
679	73	89	88	46	71	53	physical demand	temporal demand	mental demand	mental demand	mental demand
680	81	63	54	56	57	57	physical demand	temporal demand	performance	effort	frustration level
681	69	62	40	19	42	59	physical demand	mental demand	performance	effort	frustration level
682	90	98	100	57	100	58	physical demand	temporal demand	performance	mental demand	mental demand
683	48	29	43	51	48	51	mental demand	mental demand	performance	mental demand	mental demand
684	48	57	71	62	77	71	mental demand	mental demand	performance	effort	mental demand

ID	mental demand	physica l demand	tempora l demand	effort	performance	frustration level	mental demand VS physical demand	mental demand VS temporal demand	mental demand VS performance	mental demand VS effort	mental demand VS frustration level
685	63	47	25	10	70	11	mental demand	mental demand	mental demand	mental demand	mental demand
686	34	83	58	34	44	19	physical demand	temporal demand	performance	effort	frustration level
687	50	83	62	44	30	55	physical demand	temporal demand	performance	effort	frustration level
688	31	38	15	49	55	49	physical demand	mental demand	performance	effort	mental demand
689	100	90	90	91	100	100	mental demand	mental demand	mental demand	mental demand	mental demand
690	54	19	50	95	58	55	mental demand	temporal demand	performance	effort	frustration level
691	72	91	63	55	59	30	physical demand	temporal demand	mental demand	mental demand	frustration level
692	62	94	76	66	74	57	physical demand	temporal demand	performance	effort	frustration level
693	52	71	53	89	91	22	mental demand	temporal demand	mental demand	effort	frustration level
694	62	80	70	55	80	50	physical demand	temporal demand	performance	effort	mental demand
695	41	81	62	43	68	26	physical demand	temporal demand	performance	effort	frustration level
696	54	71	53	50	45	70	physical demand	mental demand	performance	effort	frustration level
697	55	42	22	22	46	39	physical demand	temporal demand	performance	effort	frustration level
698	75	100	54	79	73	32	physical demand	temporal demand	performance	effort	frustration level
699	36	61	62	26	54	13	physical demand	temporal demand	mental demand	effort	mental demand
700	35	35	40	60	40	40	physical demand	temporal demand	performance	mental demand	mental demand
701	50	50	32	32	31	33	mental demand	mental demand	performance	effort	frustration level
702	90	93	100	55	96	41	physical demand	temporal demand	mental demand	mental demand	frustration level
703	57	82	74	48	66	50	physical demand	temporal demand	performance	effort	frustration level
704	10	65	20	0	10	20	mental demand	temporal demand	performance	effort	frustration level
705	37	52	38	33	52	37	physical demand	temporal demand	performance	effort	frustration level
706	51	26	30	24	71	48	physical demand	temporal demand	performance	effort	frustration level
707	57	56	38	20	40	28	physical demand	temporal demand	performance	effort	mental demand
708	20	79	29	10	30	27	physical demand	temporal demand	performance	mental demand	frustration level
709	41	79	82	32	61	32	physical demand	mental demand	performance	mental demand	mental demand
710	50	61	45	51	59	63	physical demand	temporal demand	performance	effort	frustration level
711	49	58	44	47	27	13	mental demand	temporal demand	performance	effort	frustration level
712	92	91	93	53	100	78	mental demand	mental demand	mental demand	mental demand	frustration level
713	78	100	84	21	100	52	physical demand	temporal demand	performance	effort	mental demand
714	82	76	81	72	79	81	physical demand	temporal demand	mental demand	effort	frustration level
715	79	82	81	95	91	84	mental demand	mental demand	performance	effort	frustration level
716	92	100	95	90	100	100	mental demand	mental demand	performance	mental demand	frustration level
717	50	49	40	21	51	21	mental demand	mental demand	performance	effort	mental demand
718	100	100	100	41	100	65	physical demand	mental demand	performance	effort	frustration level
719	50	77	38	37	33	50	mental demand	temporal demand	mental demand	mental demand	mental demand
720	54	79	78	20	59	50	physical demand	mental demand	performance	effort	mental demand

ID	mental demand	physical demand	temporal demand	effort	performance	frustration level	mental demand VS physical demand	mental demand VS temporal demand	mental demand VS performance	mental demand VS effort	mental demand VS frustration level
721	80	80	60	20	60	20	physical demand	temporal demand	mental demand	effort	frustration level
722	94	95	100	46	79	61	mental demand	temporal demand	performance	effort	frustration level
723	60	60	56	59	59	37	physical demand	temporal demand	performance	effort	frustration level
724	67	88	62	40	56	15	physical demand	mental demand	mental demand	mental demand	mental demand
725	35	48	65	45	50	27	physical demand	temporal demand	performance	effort	frustration level
726	50	100	59	22	69	29	physical demand	temporal demand	performance	effort	frustration level
727	59	67	90	32	57	50	physical demand	mental demand	mental demand	mental demand	mental demand
728	51	67	89	32	62	47	physical demand	temporal demand	performance	effort	frustration level
729	70	90	50	30	66	14	physical demand	mental demand	mental demand	mental demand	mental demand
730	75	92	20	16	47	12	physical demand	temporal demand	performance	effort	mental demand
731	52	88	48	62	93	52	physical demand	temporal demand	performance	effort	frustration level
732	100	100	100	47	71	66	physical demand	temporal demand	performance	effort	frustration level
733	81	83	44	81	81	57	physical demand	temporal demand	performance	effort	frustration level
734	32	50	15	51	31	50	physical demand	temporal demand	performance	mental demand	frustration level
735	21	64	48	89	53	3	physical demand	temporal demand	performance	effort	frustration level
736	28	51	33	29	53	6	physical demand	mental demand	performance	effort	frustration level
737	46	100	90	70	84	61	physical demand	temporal demand	performance	effort	frustration level
738	40	30	73	48	22	17	mental demand	temporal demand	performance	mental demand	mental demand
739	48	51	25	39	31	10	mental demand	mental demand	performance	effort	frustration level
740	16	16	14	31	34	24	mental demand	mental demand	performance	mental demand	mental demand
741	60	60	63	29	60	13	physical demand	temporal demand	mental demand	effort	frustration level
742	36	52	35	50	54	20	mental demand	mental demand	performance	effort	frustration level
743	73	61	41	48	57	54	physical demand	mental demand	performance	mental demand	mental demand
744	93	100	88	62	91	81	physical demand	mental demand	mental demand	mental demand	frustration level
745	45	50	43	52	39	19	physical demand	mental demand	performance	effort	frustration level
746	58	64	30	54	52	38	mental demand	mental demand	performance	mental demand	mental demand
747	60	59	29	62	60	34	physical demand	mental demand	performance	effort	mental demand
748	78	100	70	76	100	100	physical demand	temporal demand	performance	effort	mental demand
749	55	59	58	55	71	15	physical demand	temporal demand	performance	effort	frustration level
750	59	34	79	57	73	44	mental demand	mental demand	performance	mental demand	frustration level
751	43	57	41	44	80	44	mental demand	temporal demand	mental demand	effort	frustration level
752	86	80	46	37	52	34	mental demand	temporal demand	performance	effort	frustration level
753	43	68	29	51	59	70	physical demand	temporal demand	performance	effort	frustration level
754	17	20	0	0	11	20	mental demand	mental demand	performance	mental demand	frustration level
755	50	50	45	55	48	57	physical demand	temporal demand	performance	effort	frustration level
756	88	88	72	88	87	80	mental demand	mental demand	mental demand	mental demand	mental demand

ID	mental demand	physical demand	temporal demand	effort	performance	frustration level	mental demand VS physical demand	mental demand VS temporal demand	mental demand VS performance	mental demand VS effort	mental demand VS frustration level
757	71	89	22	20	51	49	physical demand	mental demand	performance	effort	frustration level
758	60	60	60	40	50	10	mental demand	temporal demand	performance	effort	mental demand
759	11	15	32	28	36	0	mental demand	mental demand	performance	mental demand	mental demand
760	51	80	34	8	51	18	physical demand	mental demand	mental demand	mental demand	mental demand
761	81	83	96	62	64	62	mental demand	temporal demand	performance	mental demand	frustration level
762	100	100	70	51	79	100	mental demand	mental demand	mental demand	mental demand	mental demand
763	76	76	59	59	81	34	physical demand	temporal demand	mental demand	mental demand	mental demand
764	77	34	69	48	70	33	physical demand	temporal demand	performance	effort	frustration level
765	19	53	45	55	69	56	physical demand	temporal demand	performance	effort	frustration level
766	79	80	84	83	81	42	physical demand	temporal demand	performance	mental demand	mental demand
767	82	39	82	61	100	62	mental demand	mental demand	performance	effort	frustration level
768	93	97	100	68	87	97	mental demand	mental demand	performance	mental demand	mental demand
769	40	20	30	20	21	0	physical demand	temporal demand	performance	effort	mental demand
770	91	79	79	60	78	100	mental demand	mental demand	performance	mental demand	frustration level
771	69	73	70	31	72	51	mental demand	temporal demand	mental demand	mental demand	frustration level
772	67	100	92	44	69	33	physical demand	temporal demand	performance	effort	frustration level
773	60	71	40	40	60	63	physical demand	mental demand	performance	effort	frustration level
774	61	81	63	63	61	66	physical demand	temporal demand	performance	effort	frustration level
775	100	100	100	40	100	100	physical demand	mental demand	performance	effort	frustration level
776	78	82	77	84	85	85	physical demand	mental demand	mental demand	mental demand	mental demand
777	42	51	43	0	22	29	physical demand	mental demand	mental demand	effort	mental demand
778	68	79	61	72	65	69	physical demand	mental demand	mental demand	effort	mental demand
779	35	74	96	59	58	75	physical demand	temporal demand	performance	mental demand	mental demand
780	49	53	22	15	47	13	physical demand	temporal demand	performance	effort	frustration level
781	28	28	20	51	23	15	mental demand	temporal demand	performance	effort	frustration level
782	90	91	81	86	86	78	physical demand	temporal demand	performance	effort	frustration level
783	54	68	70	75	70	71	physical demand	temporal demand	performance	effort	frustration level
784	65	61	42	40	58	59	mental demand	mental demand	performance	effort	frustration level
785	68	85	70	32	39	16	physical demand	temporal demand	performance	effort	frustration level
786	79	43	40	40	61	14	mental demand	mental demand	performance	effort	frustration level
787	58	51	33	33	50	10	physical demand	temporal demand	performance	effort	frustration level
788	55	70	65	45	50	30	physical demand	temporal demand	performance	effort	mental demand
789	60	85	78	77	82	80	mental demand	temporal demand	performance	effort	frustration level
790	76	63	66	63	74	43	physical demand	temporal demand	performance	effort	frustration level
791	50	70	61	40	60	80	physical demand	temporal demand	mental demand	mental demand	frustration level
792	53	58	52	55	55	53	physical demand	temporal demand	performance	mental demand	mental demand

ID	mental demand	physical demand	temporal demand	effort	performance	frustration level	mental demand VS physical demand	mental demand VS temporal demand	mental demand VS performance	mental demand VS effort	mental demand VS frustration level
793	45	41	41	24	59	52	mental demand	mental demand	performance	mental demand	frustration level
794	48	76	48	32	52	11	physical demand	temporal demand	mental demand	effort	frustration level
795	80	80	80	60	80	80	mental demand	temporal demand	performance	mental demand	frustration level
796	66	73	81	64	79	89	mental demand	temporal demand	performance	effort	frustration level
797	52	58	60	60	58	32	mental demand	temporal demand	performance	mental demand	mental demand
798	80	69	60	60	60	50	mental demand	temporal demand	performance	effort	frustration level
799	84	75	59	52	83	88	mental demand	mental demand	mental demand	mental demand	mental demand
800	81	60	35	51	94	87	physical demand	mental demand	mental demand	mental demand	frustration level
801	55	70	77	31	45	41	physical demand	temporal demand	performance	effort	mental demand
802	35	61	51	75	60	50	physical demand	temporal demand	performance	effort	frustration level
803	20	30	20	20	25	10	physical demand	temporal demand	performance	effort	mental demand
804	50	74	100	0	51	0	physical demand	temporal demand	performance	effort	frustration level
805	31	52	50	60	49	30	physical demand	mental demand	performance	mental demand	frustration level
806	60	70	50	20	85	8	mental demand	mental demand	mental demand	mental demand	mental demand
807	82	83	60	61	88	46	mental demand	temporal demand	performance	effort	frustration level
808	78	61	58	42	55	31	mental demand	temporal demand	performance	effort	frustration level
809	80	80	80	23	80	61	physical demand	mental demand	mental demand	mental demand	mental demand
810	100	100	100	100	100	100	physical demand	temporal demand	performance	effort	frustration level
811	80	100	100	22	81	100	physical demand	temporal demand	performance	mental demand	frustration level
812	59	100	100	49	28	17	physical demand	temporal demand	performance	effort	mental demand
813	50	50	50	22	60	0	mental demand	mental demand	mental demand	mental demand	mental demand
814	59	100	100	58	100	41	physical demand	temporal demand	performance	effort	frustration level
815	77	79	78	54	94	89	mental demand	temporal demand	performance	mental demand	frustration level
816	45	40	60	40	50	20	physical demand	temporal demand	performance	effort	frustration level
817	63	77	57	54	55	55	physical demand	temporal demand	performance	effort	frustration level
818	51	27	76	20	58	60	mental demand	temporal demand	performance	effort	frustration level
819	94	63	100	96	79	100	mental demand	mental demand	mental demand	mental demand	frustration level
820	86	100	69	27	92	100	physical demand	temporal demand	mental demand	mental demand	mental demand
821	100	85	100	100	58	79	mental demand	temporal demand	performance	mental demand	frustration level
822	52	37	57	51	41	20	mental demand	temporal demand	performance	effort	mental demand
823	51	37	56	78	86	46	physical demand	temporal demand	performance	effort	frustration level
824	66	68	25	43	80	41	physical demand	mental demand	mental demand	mental demand	frustration level
825	38	67	36	33	39	21	physical demand	temporal demand	performance	effort	frustration level
826	58	64	82	64	68	86	physical demand	temporal demand	performance	effort	frustration level
827	93	77	97	6	100	89	mental demand	temporal demand	performance	mental demand	mental demand
828	63	64	62	61	61	62	physical demand	temporal demand	performance	effort	mental demand

ID	mental demand	physical demand	temporal demand	effort	performance	frustration level	mental demand VS physical demand	mental demand VS temporal demand	mental demand VS performance	mental demand VS effort	mental demand VS frustration level
829	41	100	71	59	100	100	physical demand	temporal demand	performance	effort	frustration level
830	61	30	78	40	56	7	mental demand	temporal demand	performance	effort	frustration level
831	50	50	66	50	60	53	physical demand	temporal demand	performance	effort	mental demand
832	50	90	50	70	82	43	physical demand	temporal demand	performance	mental demand	frustration level
833	91	93	92	60	60	93	mental demand	temporal demand	performance	mental demand	frustration level
834	50	30	29	30	43	22	mental demand	temporal demand	mental demand	mental demand	frustration level
835	38	54	53	11	36	12	physical demand	temporal demand	performance	effort	mental demand
836	93	100	96	5	81	91	mental demand	mental demand	performance	effort	frustration level
837	63	38	64	64	69	63	mental demand	temporal demand	performance	effort	mental demand
838	61	61	63	53	55	30	physical demand	mental demand	performance	effort	frustration level
839	69	53	65	57	70	52	physical demand	mental demand	performance	mental demand	frustration level
840	61	73	53	71	100	98	mental demand	mental demand	performance	effort	mental demand
841	59	78	63	65	64	31	physical demand	temporal demand	performance	effort	frustration level
842	50	51	53	53	53	54	physical demand	temporal demand	performance	mental demand	frustration level
843	8	12	3	12	12	15	mental demand	mental demand	performance	effort	frustration level
844	59	23	18	25	67	25	physical demand	temporal demand	performance	effort	frustration level
845	61	61	59	59	58	61	physical demand	mental demand	performance	mental demand	frustration level
846	35	17	81	61	23	15	mental demand	temporal demand	mental demand	effort	frustration level
847	31	79	39	34	33	19	physical demand	temporal demand	performance	mental demand	mental demand
848	38	65	45	48	44	50	physical demand	temporal demand	mental demand	mental demand	frustration level
849	55	99	71	30	85	20	physical demand	temporal demand	mental demand	mental demand	mental demand
850	50	51	40	50	30	10	mental demand	mental demand	performance	effort	mental demand
851	67	72	73	53	73	70	mental demand	temporal demand	performance	effort	frustration level
852	84	47	73	51	100	78	mental demand	temporal demand	performance	effort	mental demand
853	88	92	85	40	80	70	mental demand	mental demand	performance	mental demand	mental demand
854	71	54	47	42	55	57	mental demand	mental demand	mental demand	mental demand	mental demand
855	40	76	57	58	57	23	physical demand	temporal demand	performance	mental demand	mental demand
856	100	100	100	50	100	80	physical demand	temporal demand	mental demand	effort	mental demand
857	78	96	60	86	70	30	physical demand	mental demand	mental demand	effort	mental demand
858	71	76	71	84	94	61	mental demand	mental demand	performance	effort	frustration level
859	71	50	65	52	50	47	mental demand	mental demand	mental demand	mental demand	frustration level
860	50	68	51	40	42	68	mental demand	temporal demand	performance	effort	frustration level
861	89	100	76	61	95	100	mental demand	temporal demand	performance	effort	frustration level
862	63	68	55	49	86	33	mental demand	temporal demand	performance	mental demand	mental demand
863	50	20	35	40	40	41	mental demand	temporal demand	performance	effort	frustration level
864	55	67	72	58	71	56	mental demand	mental demand	performance	mental demand	frustration level

ID	mental demand	physical demand	temporal demand	effort	performance	frustration level	mental demand VS physical demand	mental demand VS temporal demand	mental demand VS performance	mental demand VS effort	mental demand VS frustration level
865	85	100	89	34	91	14	physical demand	temporal demand	mental demand	effort	mental demand
866	41	80	76	14	100	15	physical demand	temporal demand	performance	effort	mental demand
867	40	40	40	40	40	40	mental demand	mental demand	performance	effort	frustration level
868	53	48	72	52	72	52	physical demand	mental demand	performance	mental demand	frustration level
869	50	30	40	36	35	13	physical demand	mental demand	mental demand	mental demand	mental demand
870	32	81	41	52	60	26	mental demand	mental demand	performance	effort	frustration level
871	72	85	52	27	68	28	mental demand	mental demand	mental demand	mental demand	mental demand
872	50	30	36	0	12	0	mental demand	temporal demand	mental demand	mental demand	mental demand
873	67	68	37	31	33	28	mental demand	mental demand	mental demand	mental demand	mental demand
874	70	55	61	65	64	22	mental demand	mental demand	performance	mental demand	mental demand
875	62	61	54	27	58	42	mental demand	mental demand	mental demand	mental demand	mental demand
876	80	89	85	59	79	65	mental demand	mental demand	mental demand	mental demand	mental demand
877	53	53	41	53	50	13	physical demand	temporal demand	mental demand	mental demand	mental demand
878	54	59	54	38	56	31	physical demand	temporal demand	performance	effort	frustration level
879	61	80	50	80	80	100	physical demand	mental demand	performance	effort	frustration level
880	72	55	35	26	67	8	physical demand	mental demand	performance	mental demand	mental demand
881	57	79	75	35	66	30	physical demand	temporal demand	performance	mental demand	mental demand
882	43	51	51	88	78	42	mental demand	mental demand	performance	mental demand	frustration level
883	77	50	91	84	71	39	mental demand	mental demand	performance	effort	frustration level
884	100	83	91	0	75	0	physical demand	temporal demand	performance	effort	mental demand
885	98	100	94	85	100	49	mental demand	temporal demand	performance	effort	frustration level
886	60	57	47	58	49	43	physical demand	temporal demand	performance	effort	frustration level
887	60	95	40	50	41	40	physical demand	mental demand	performance	effort	frustration level
888	69	86	71	85	78	76	physical demand	mental demand	performance	effort	frustration level
889	84	62	79	24	45	13	mental demand	temporal demand	performance	mental demand	mental demand
890	93	58	83	38	74	43	mental demand	mental demand	mental demand	mental demand	frustration level
891	70	84	70	72	89	81	mental demand	mental demand	performance	mental demand	mental demand
892	60	60	80	24	84	46	physical demand	mental demand	performance	effort	frustration level
893	84	84	81	57	74	82	mental demand	mental demand	mental demand	mental demand	mental demand
894	71	61	65	50	42	20	physical demand	temporal demand	performance	effort	frustration level
895	80	44	93	40	67	44	mental demand	temporal demand	performance	effort	mental demand
896	64	67	85	57	66	18	mental demand	temporal demand	mental demand	mental demand	mental demand
897	73	66	78	61	68	84	physical demand	temporal demand	performance	effort	frustration level
898	77	61	100	49	68	100	physical demand	temporal demand	mental demand	mental demand	mental demand
899	53	54	47	77	50	47	physical demand	mental demand	performance	mental demand	frustration level
900	31	82	60	50	57	39	physical demand	temporal demand	performance	mental demand	frustration level

ID	mental demand	physical demand	temporal demand	effort	performance	frustration level	mental demand VS physical demand	mental demand VS temporal demand	mental demand VS performance	mental demand VS effort	mental demand VS frustration level
901	65	77	43	97	89	9	mental demand	mental demand	performance	effort	frustration level
902	100	93	98	11	100	100	mental demand	mental demand	performance	effort	mental demand
903	80	60	39	54	53	49	mental demand	mental demand	mental demand	mental demand	mental demand
904	91	98	79	15	93	32	physical demand	mental demand	performance	mental demand	frustration level
905	46	80	69	51	59	55	physical demand	mental demand	mental demand	mental demand	mental demand
906	58	59	89	43	95	83	mental demand	mental demand	mental demand	effort	frustration level
907	77	71	69	64	69	66	mental demand	mental demand	mental demand	mental demand	mental demand
908	100	81	100	60	60	41	mental demand	temporal demand	performance	effort	frustration level
909	24	61	20	42	28	39	physical demand	temporal demand	performance	effort	mental demand
910	77	26	86	10	100	27	mental demand	mental demand	mental demand	mental demand	mental demand
911	87	82	86	80	81	85	physical demand	temporal demand	performance	effort	frustration level
912	60	40	40	59	60	39	mental demand	temporal demand	performance	effort	frustration level
913	61	62	57	50	52	32	physical demand	temporal demand	mental demand	mental demand	mental demand
914	60	43	43	37	36	17	mental demand	temporal demand	performance	effort	frustration level
915	36	17	16	13	15	12	physical demand	temporal demand	performance	effort	mental demand
916	80	62	78	40	56	18	mental demand	mental demand	performance	effort	frustration level
917	61	90	60	40	61	58	physical demand	mental demand	performance	effort	mental demand
918	39	19	56	25	37	18	physical demand	temporal demand	performance	effort	frustration level
919	70	80	70	50	60	50	mental demand	temporal demand	mental demand	mental demand	mental demand
920	77	85	100	50	76	40	physical demand	temporal demand	performance	mental demand	frustration level
921	66	88	53	50	87	37	mental demand	temporal demand	performance	mental demand	mental demand
922	77	100	81	85	78	80	physical demand	mental demand	performance	mental demand	mental demand
923	75	90	60	35	71	30	physical demand	temporal demand	performance	mental demand	frustration level
924	13	33	14	11	25	7	physical demand	temporal demand	performance	mental demand	mental demand
925	70	100	100	54	61	0	physical demand	mental demand	mental demand	effort	frustration level
926	63	100	75	62	86	22	physical demand	mental demand	performance	mental demand	frustration level
927	61	95	100	83	100	72	mental demand	mental demand	mental demand	mental demand	mental demand
928	34	23	46	50	51	11	mental demand	mental demand	performance	effort	mental demand
929	54	80	61	29	52	28	physical demand	temporal demand	performance	effort	frustration level
930	43	100	38	68	41	14	mental demand	mental demand	performance	mental demand	mental demand
931	60	98	72	51	72	64	physical demand	mental demand	performance	effort	frustration level
932	37	53	15	10	30	10	mental demand	temporal demand	performance	effort	mental demand
933	22	18	22	9	20	5	mental demand	temporal demand	mental demand	mental demand	mental demand
934	55	40	47	50	52	20	physical demand	mental demand	performance	mental demand	frustration level
935	33	28	42	38	32	27	mental demand	mental demand	mental demand	mental demand	mental demand
936	100	84	85	39	92	48	physical demand	temporal demand	performance	effort	mental demand

ID	mental demand	physical demand	temporal demand	effort	performance	frustration level	mental demand VS physical demand	mental demand VS temporal demand	mental demand VS performance	mental demand VS effort	mental demand VS frustration level
937	44	62	29	51	60	16	physical demand	temporal demand	performance	effort	mental demand
938	60	55	55	41	60	29	mental demand	temporal demand	performance	mental demand	frustration level
939	36	20	20	9	8	0	physical demand	mental demand	performance	effort	frustration level
940	77	100	97	94	94	99	physical demand	mental demand	mental demand	mental demand	mental demand
941	57	100	94	16	82	65	physical demand	temporal demand	performance	effort	frustration level
942	40	80	100	23	54	23	physical demand	temporal demand	performance	effort	frustration level
943	95	90	55	95	60	10	mental demand	temporal demand	performance	mental demand	mental demand
944	15	21	12	2	45	3	physical demand	temporal demand	performance	effort	frustration level
945	32	50	25	20	30	10	physical demand	temporal demand	mental demand	effort	mental demand
946	63	53	50	45	67	20	physical demand	temporal demand	performance	effort	frustration level
947	26	89	79	32	100	22	physical demand	mental demand	performance	effort	frustration level
948	100	100	100	50	100	90	physical demand	mental demand	performance	mental demand	mental demand
949	30	11	30	46	27	45	mental demand	mental demand	mental demand	mental demand	mental demand
950	52	81	35	62	51	40	physical demand	mental demand	performance	effort	frustration level
951	2	5	4	2	4	3	mental demand	mental demand	performance	mental demand	frustration level
952	100	100	100	90	100	68	physical demand	mental demand	performance	mental demand	frustration level
953	85	83	91	44	58	48	physical demand	mental demand	performance	effort	frustration level
954	48	18	19	100	54	66	mental demand	temporal demand	performance	mental demand	frustration level
955	59	100	93	36	58	51	physical demand	temporal demand	performance	effort	mental demand
956	47	58	80	15	55	49	physical demand	mental demand	performance	mental demand	frustration level
957	76	65	60	70	70	55	mental demand	mental demand	mental demand	effort	frustration level
958	34	67	23	41	35	30	physical demand	temporal demand	performance	effort	mental demand
959	75	80	80	56	58	53	physical demand	temporal demand	performance	effort	frustration level
960	70	79	43	60	53	60	physical demand	temporal demand	mental demand	effort	frustration level
961	78	65	50	52	48	4	physical demand	temporal demand	mental demand	effort	mental demand
962	59	61	60	7	61	5	physical demand	temporal demand	performance	effort	mental demand
963	86	84	72	36	68	30	physical demand	temporal demand	performance	effort	frustration level
964	85	72	52	60	32	34	physical demand	mental demand	performance	effort	frustration level
965	40	43	52	22	50	12	physical demand	temporal demand	performance	effort	frustration level
966	63	40	70	14	48	21	physical demand	temporal demand	performance	mental demand	mental demand
967	53	50	91	13	55	11	physical demand	temporal demand	performance	mental demand	frustration level
968	79	91	52	0	78	90	mental demand	mental demand	performance	mental demand	frustration level
969	65	49	59	38	43	22	physical demand	temporal demand	performance	effort	mental demand
970	71	56	79	49	59	59	mental demand	temporal demand	mental demand	mental demand	mental demand
971	59	79	69	53	61	48	physical demand	temporal demand	performance	mental demand	mental demand
972	43	81	77	11	40	37	physical demand	temporal demand	performance	effort	mental demand

ID	mental demand	physical demand	temporal demand	effort	performance	frustration level	mental demand VS physical demand	mental demand VS temporal demand	mental demand VS performance	mental demand VS effort	mental demand VS frustration level
973	60	55	56	38	35	58	mental demand	temporal demand	performance	effort	frustration level
974	83	87	90	38	82	14	mental demand	mental demand	performance	effort	mental demand
975	50	44	66	47	77	36	mental demand	mental demand	performance	mental demand	frustration level
976	62	46	22	26	41	11	physical demand	temporal demand	mental demand	effort	frustration level
977	100	100	94	43	80	59	mental demand	mental demand	performance	effort	frustration level
978	30	40	50	40	52	33	mental demand	mental demand	performance	mental demand	mental demand
979	56	55	56	53	58	66	physical demand	temporal demand	mental demand	effort	frustration level
980	55	51	57	69	0	40	mental demand	mental demand	mental demand	effort	mental demand
981	61	27	41	58	61	30	mental demand	temporal demand	performance	effort	frustration level
982	43	47	28	52	49	24	physical demand	temporal demand	performance	mental demand	frustration level
983	32	51	52	38	50	12	physical demand	temporal demand	performance	mental demand	frustration level
984	70	77	69	75	69	83	mental demand	temporal demand	performance	mental demand	mental demand
985	35	29	53	48	33	32	physical demand	temporal demand	performance	effort	frustration level
986	46	75	54	33	84	41	physical demand	temporal demand	performance	effort	frustration level
987	52	62	59	59	49	53	physical demand	temporal demand	mental demand	mental demand	frustration level
988	60	57	31	23	65	25	physical demand	temporal demand	performance	effort	frustration level
989	85	100	89	59	100	72	mental demand	mental demand	mental demand	mental demand	mental demand
990	62	62	48	43	49	31	physical demand	temporal demand	performance	effort	mental demand
991	50	50	50	20	50	20	mental demand	temporal demand	performance	effort	mental demand
992	79	79	84	60	80	81	mental demand	mental demand	mental demand	mental demand	mental demand
993	67	75	33	16	57	18	physical demand	temporal demand	performance	effort	frustration level
994	34	80	68	68	59	51	physical demand	temporal demand	mental demand	mental demand	mental demand
995	72	83	69	83	86	64	physical demand	temporal demand	performance	effort	mental demand
996	48	50	68	45	50	5	physical demand	temporal demand	performance	effort	mental demand
997	83	80	64	20	59	78	mental demand	temporal demand	performance	effort	mental demand
998	47	78	53	41	82	26	physical demand	temporal demand	mental demand	mental demand	frustration level
999	29	32	26	16	55	21	physical demand	temporal demand	mental demand	effort	mental demand
1000	100	100	54	35	67	31	physical demand	mental demand	mental demand	mental demand	mental demand
1001	48	83	46	26	26	14	physical demand	temporal demand	performance	effort	frustration level
1002	17	22	28	72	47	24	physical demand	mental demand	mental demand	mental demand	mental demand
1003	51	45	28	14	25	13	physical demand	temporal demand	performance	effort	frustration level
1004	65	96	64	17	60	30	physical demand	temporal demand	mental demand	effort	frustration level
