# Supplementary material for: Factoring and correlation in sleep, fatigue and mental workload of clinical first-line nurses in the post-pandemic era of COVID-19: A multi-center cross-sectional study
Source: Front Psychiatry. 2022 Aug 25;13:963419. doi: 10.3389/fpsyt.2022.963419 (PMC9452657; doi:10.3389/fpsyt.2022.963419)
Supplement: Supplementary file 2 [file Data_Sheet_2.PDF]

ID	physical demand VS temporal demand	physical demand VS performance	physical demand VS effort	physical demand VS frustration level	temporal demand VS performance	temporal demand VS effort
1	temporal demand	performance	effort	frustration level	temporal demand	temporal demand
2	temporal demand	performance	physical demand	physical demand	performance	temporal demand
3	temporal demand	performance	physical demand	frustration level	performance	temporal demand
4	temporal demand	physical demand	physical demand	physical demand	performance	temporal demand
5	temporal demand	performance	physical demand	frustration level	performance	temporal demand
6	temporal demand	performance	effort	frustration level	temporal demand	temporal demand
7	physical demand	performance	effort	physical demand	performance	effort
8	temporal demand	performance	effort	physical demand	performance	temporal demand
9	physical demand	physical demand	physical demand	physical demand	performance	temporal demand
10	physical demand	performance	effort	frustration level	performance	effort
11	temporal demand	performance	effort	frustration level	performance	effort
12	physical demand	physical demand	physical demand	physical demand	temporal demand	temporal demand
13	physical demand	performance	physical demand	frustration level	temporal demand	effort
14	physical demand	physical demand	physical demand	physical demand	temporal demand	temporal demand
15	temporal demand	physical demand	physical demand	physical demand	temporal demand	temporal demand
16	temporal demand	performance	effort	physical demand	temporal demand	effort
17	physical demand	performance	effort	frustration level	performance	effort
18	physical demand	performance	physical demand	physical demand	temporal demand	temporal demand
19	physical demand	physical demand	physical demand	physical demand	temporal demand	effort
20	temporal demand	performance	physical demand	frustration level	performance	effort
21	physical demand	physical demand	physical demand	physical demand	performance	effort
22	temporal demand	performance	effort	physical demand	performance	temporal demand
23	temporal demand	performance	effort	frustration level	performance	temporal demand
24	physical demand	performance	physical demand	physical demand	performance	temporal demand
25	physical demand	performance	physical demand	physical demand	temporal demand	effort
26	physical demand	physical demand	physical demand	physical demand	temporal demand	temporal demand
27	temporal demand	physical demand	effort	physical demand	performance	effort
28	physical demand	performance	effort	physical demand	performance	effort
29	temporal demand	performance	physical demand	frustration level	performance	temporal demand
30	physical demand	performance	physical demand	frustration level	performance	effort
31	temporal demand	physical demand	physical demand	physical demand	performance	temporal demand
32	physical demand	physical demand	physical demand	physical demand	performance	temporal demand
33	temporal demand	physical demand	physical demand	frustration level	temporal demand	temporal demand
34	physical demand	performance	effort	frustration level	performance	effort
35	physical demand	performance	effort	frustration level	performance	effort
36	physical demand	performance	effort	physical demand	performance	temporal demand

ID	physical demand VS temporal demand	physical demand VS performance	physical demand VS effort	physical demand VS frustration level	temporal demand VS performance	temporal demand VS effort
37	physical demand	physical demand	physical demand	physical demand	temporal demand	effort
38	physical demand	performance	physical demand	physical demand	performance	temporal demand
39	physical demand	performance	effort	frustration level	performance	effort
40	physical demand	physical demand	physical demand	physical demand	temporal demand	temporal demand
41	physical demand	physical demand	physical demand	physical demand	temporal demand	effort
42	physical demand	performance	physical demand	physical demand	temporal demand	temporal demand
43	temporal demand	physical demand	physical demand	physical demand	temporal demand	temporal demand
44	temporal demand	performance	effort	frustration level	performance	effort
45	physical demand	physical demand	physical demand	physical demand	performance	effort
46	physical demand	performance	effort	physical demand	performance	effort
47	physical demand	performance	effort	frustration level	performance	effort
48	physical demand	performance	effort	frustration level	performance	effort
49	physical demand	performance	effort	frustration level	performance	effort
50	physical demand	physical demand	physical demand	physical demand	temporal demand	effort
51	temporal demand	performance	effort	physical demand	performance	effort
52	physical demand	physical demand	physical demand	physical demand	temporal demand	temporal demand
53	temporal demand	performance	effort	physical demand	performance	effort
54	physical demand	physical demand	physical demand	physical demand	temporal demand	effort
55	temporal demand	performance	effort	frustration level	performance	temporal demand
56	temporal demand	performance	effort	frustration level	temporal demand	temporal demand
57	physical demand	performance	effort	frustration level	temporal demand	effort
58	physical demand	physical demand	physical demand	physical demand	performance	temporal demand
59	temporal demand	performance	effort	frustration level	performance	effort
60	temporal demand	performance	physical demand	frustration level	performance	temporal demand
61	physical demand	physical demand	physical demand	physical demand	temporal demand	effort
62	physical demand	physical demand	physical demand	physical demand	temporal demand	temporal demand
63	physical demand	performance	effort	frustration level	performance	effort
64	physical demand	physical demand	physical demand	physical demand	temporal demand	temporal demand
65	temporal demand	performance	effort	frustration level	performance	effort
66	physical demand	physical demand	effort	frustration level	temporal demand	effort
67	temporal demand	performance	physical demand	physical demand	temporal demand	temporal demand
68	temporal demand	performance	physical demand	frustration level	performance	temporal demand
69	temporal demand	performance	effort	frustration level	temporal demand	effort
70	physical demand	performance	effort	frustration level	performance	effort
71	physical demand	physical demand	physical demand	physical demand	temporal demand	temporal demand
72	physical demand	physical demand	physical demand	physical demand	performance	temporal demand

ID	physical demand VS temporal demand	physical demand VS performance	physical demand VS effort	physical demand VS frustration level	temporal demand VS performance	temporal demand VS effort
73	temporal demand	performance	effort	physical demand	temporal demand	temporal demand
74	physical demand	physical demand	physical demand	physical demand	temporal demand	temporal demand
75	physical demand	performance	physical demand	physical demand	temporal demand	temporal demand
76	physical demand	physical demand	physical demand	physical demand	performance	effort
77	temporal demand	performance	physical demand	frustration level	performance	temporal demand
78	temporal demand	performance	effort	physical demand	temporal demand	temporal demand
79	physical demand	performance	effort	frustration level	performance	effort
80	temporal demand	performance	effort	frustration level	performance	effort
81	physical demand	physical demand	physical demand	physical demand	performance	temporal demand
82	temporal demand	performance	physical demand	frustration level	performance	effort
83	physical demand	physical demand	physical demand	physical demand	temporal demand	temporal demand
84	temporal demand	performance	effort	frustration level	performance	effort
85	temporal demand	performance	physical demand	frustration level	performance	effort
86	physical demand	physical demand	physical demand	physical demand	performance	temporal demand
87	temporal demand	performance	effort	frustration level	performance	effort
88	temporal demand	performance	effort	frustration level	temporal demand	effort
89	physical demand	physical demand	effort	frustration level	performance	effort
90	temporal demand	performance	effort	frustration level	performance	effort
91	physical demand	performance	physical demand	frustration level	performance	effort
92	physical demand	physical demand	physical demand	physical demand	performance	effort
93	physical demand	performance	effort	frustration level	temporal demand	effort
94	physical demand	physical demand	physical demand	frustration level	temporal demand	temporal demand
95	temporal demand	performance	effort	physical demand	temporal demand	effort
96	physical demand	physical demand	effort	physical demand	temporal demand	temporal demand
97	physical demand	physical demand	physical demand	physical demand	performance	effort
98	temporal demand	performance	effort	frustration level	performance	temporal demand
99	physical demand	physical demand	effort	physical demand	temporal demand	effort
100	physical demand	performance	effort	physical demand	performance	effort
101	temporal demand	performance	effort	frustration level	performance	effort
102	physical demand	performance	physical demand	physical demand	temporal demand	temporal demand
103	physical demand	performance	effort	physical demand	performance	effort
104	physical demand	physical demand	physical demand	physical demand	temporal demand	temporal demand
105	temporal demand	performance	physical demand	frustration level	temporal demand	temporal demand
106	physical demand	physical demand	physical demand	physical demand	temporal demand	temporal demand
107	physical demand	physical demand	physical demand	physical demand	performance	effort
108	temporal demand	performance	physical demand	physical demand	performance	temporal demand

ID	physical demand VS temporal demand	physical demand VS performance	physical demand VS effort	physical demand VS frustration level	temporal demand VS performance	temporal demand VS effort
109	physical demand	performance	effort	frustration level	temporal demand	temporal demand
110	physical demand	performance	physical demand	frustration level	temporal demand	temporal demand
111	temporal demand	performance	physical demand	frustration level	temporal demand	temporal demand
112	physical demand	performance	effort	physical demand	performance	effort
113	temporal demand	performance	effort	frustration level	temporal demand	temporal demand
114	physical demand	performance	physical demand	physical demand	temporal demand	temporal demand
115	temporal demand	performance	physical demand	frustration level	performance	temporal demand
116	physical demand	physical demand	physical demand	physical demand	temporal demand	temporal demand
117	temporal demand	performance	physical demand	frustration level	temporal demand	temporal demand
118	physical demand	performance	physical demand	frustration level	performance	effort
119	temporal demand	performance	effort	frustration level	performance	effort
120	temporal demand	performance	physical demand	frustration level	performance	temporal demand
121	temporal demand	performance	effort	frustration level	performance	effort
122	physical demand	physical demand	physical demand	physical demand	performance	effort
123	temporal demand	performance	effort	frustration level	temporal demand	temporal demand
124	physical demand	performance	physical demand	frustration level	performance	temporal demand
125	physical demand	physical demand	physical demand	physical demand	temporal demand	temporal demand
126	physical demand	physical demand	physical demand	physical demand	performance	temporal demand
127	physical demand	physical demand	physical demand	physical demand	temporal demand	temporal demand
128	temporal demand	performance	physical demand	frustration level	temporal demand	temporal demand
129	physical demand	performance	effort	frustration level	performance	effort
130	physical demand	performance	physical demand	physical demand	temporal demand	temporal demand
131	physical demand	physical demand	physical demand	physical demand	temporal demand	temporal demand
132	temporal demand	performance	effort	physical demand	performance	effort
133	physical demand	performance	effort	frustration level	performance	effort
134	temporal demand	performance	physical demand	frustration level	performance	effort
135	temporal demand	performance	effort	frustration level	performance	effort
136	physical demand	physical demand	physical demand	physical demand	performance	temporal demand
137	physical demand	performance	effort	frustration level	performance	effort
138	physical demand	performance	effort	physical demand	temporal demand	effort
139	physical demand	physical demand	physical demand	physical demand	performance	temporal demand
140	temporal demand	physical demand	physical demand	frustration level	temporal demand	effort
141	physical demand	physical demand	physical demand	physical demand	temporal demand	temporal demand
142	temporal demand	performance	physical demand	frustration level	performance	temporal demand
143	temporal demand	performance	physical demand	physical demand	temporal demand	temporal demand
144	temporal demand	physical demand	physical demand	physical demand	performance	effort

ID	physical demand VS temporal demand	physical demand VS performance	physical demand VS effort	physical demand VS frustration level	temporal demand VS performance	temporal demand VS effort
145	physical demand	physical demand	physical demand	frustration level	performance	effort
146	physical demand	performance	physical demand	frustration level	performance	temporal demand
147	physical demand	physical demand	physical demand	physical demand	performance	temporal demand
148	physical demand	performance	physical demand	physical demand	temporal demand	temporal demand
149	temporal demand	physical demand	effort	frustration level	performance	effort
150	temporal demand	performance	physical demand	physical demand	temporal demand	temporal demand
151	temporal demand	performance	physical demand	physical demand	performance	temporal demand
152	temporal demand	performance	effort	frustration level	performance	effort
153	physical demand	performance	physical demand	physical demand	temporal demand	temporal demand
154	physical demand	physical demand	physical demand	frustration level	performance	effort
155	physical demand	physical demand	physical demand	physical demand	performance	effort
156	physical demand	physical demand	physical demand	physical demand	temporal demand	temporal demand
157	physical demand	physical demand	physical demand	physical demand	temporal demand	temporal demand
158	temporal demand	physical demand	effort	physical demand	temporal demand	effort
159	physical demand	physical demand	physical demand	frustration level	performance	effort
160	physical demand	performance	effort	frustration level	performance	temporal demand
161	physical demand	performance	effort	frustration level	performance	effort
162	physical demand	performance	physical demand	physical demand	temporal demand	temporal demand
163	physical demand	performance	physical demand	frustration level	performance	effort
164	physical demand	performance	physical demand	frustration level	performance	effort
165	physical demand	performance	effort	physical demand	temporal demand	temporal demand
166	physical demand	performance	physical demand	frustration level	performance	effort
167	temporal demand	performance	effort	frustration level	performance	effort
168	physical demand	physical demand	physical demand	physical demand	performance	temporal demand
169	physical demand	performance	effort	frustration level	performance	effort
170	temporal demand	physical demand	physical demand	physical demand	temporal demand	temporal demand
171	temporal demand	performance	effort	frustration level	performance	temporal demand
172	physical demand	performance	effort	physical demand	temporal demand	temporal demand
173	temporal demand	performance	effort	physical demand	temporal demand	temporal demand
174	physical demand	physical demand	physical demand	frustration level	temporal demand	effort
175	temporal demand	performance	effort	frustration level	performance	effort
176	temporal demand	performance	effort	frustration level	performance	effort
177	temporal demand	physical demand	effort	physical demand	performance	temporal demand
178	physical demand	performance	effort	physical demand	performance	temporal demand
179	temporal demand	performance	effort	physical demand	temporal demand	temporal demand
180	physical demand	physical demand	physical demand	physical demand	performance	temporal demand

ID	physical demand VS temporal demand	physical demand VS performance	physical demand VS effort	physical demand VS frustration level	temporal demand VS performance	temporal demand VS effort
181	temporal demand	performance	effort	physical demand	performance	temporal demand
182	physical demand	performance	physical demand	frustration level	performance	effort
183	physical demand	physical demand	physical demand	physical demand	performance	temporal demand
184	temporal demand	performance	physical demand	physical demand	temporal demand	temporal demand
185	physical demand	performance	effort	physical demand	temporal demand	effort
186	physical demand	performance	physical demand	physical demand	performance	temporal demand
187	temporal demand	performance	physical demand	physical demand	temporal demand	temporal demand
188	physical demand	performance	physical demand	frustration level	performance	temporal demand
189	temporal demand	physical demand	effort	physical demand	temporal demand	temporal demand
190	temporal demand	physical demand	physical demand	physical demand	temporal demand	temporal demand
191	physical demand	physical demand	physical demand	physical demand	temporal demand	temporal demand
192	temporal demand	performance	physical demand	frustration level	performance	temporal demand
193	temporal demand	performance	physical demand	physical demand	performance	temporal demand
194	temporal demand	performance	physical demand	frustration level	temporal demand	effort
195	physical demand	physical demand	physical demand	physical demand	performance	temporal demand
196	temporal demand	physical demand	physical demand	physical demand	temporal demand	temporal demand
197	physical demand	performance	effort	frustration level	performance	effort
198	temporal demand	physical demand	physical demand	physical demand	temporal demand	temporal demand
199	temporal demand	performance	effort	physical demand	performance	effort
200	physical demand	physical demand	physical demand	physical demand	temporal demand	temporal demand
201	physical demand	performance	effort	physical demand	temporal demand	effort
202	temporal demand	performance	effort	frustration level	temporal demand	temporal demand
203	temporal demand	performance	effort	physical demand	performance	effort
204	physical demand	physical demand	physical demand	physical demand	temporal demand	effort
205	physical demand	physical demand	physical demand	physical demand	temporal demand	temporal demand
206	temporal demand	performance	effort	frustration level	temporal demand	temporal demand
207	physical demand	performance	effort	frustration level	performance	temporal demand
208	temporal demand	performance	physical demand	frustration level	temporal demand	effort
209	physical demand	physical demand	physical demand	physical demand	performance	effort
210	temporal demand	performance	effort	frustration level	performance	effort
211	temporal demand	performance	physical demand	frustration level	performance	temporal demand
212	physical demand	performance	effort	frustration level	performance	effort
213	temporal demand	performance	effort	physical demand	performance	effort
214	temporal demand	physical demand	physical demand	physical demand	temporal demand	temporal demand
215	temporal demand	performance	physical demand	physical demand	temporal demand	effort
216	temporal demand	performance	physical demand	physical demand	temporal demand	temporal demand

ID	physical demand VS temporal demand	physical demand VS performance	physical demand VS effort	physical demand VS frustration level	temporal demand VS performance	temporal demand VS effort
217	temporal demand	performance	effort	frustration level	performance	temporal demand
218	temporal demand	performance	effort	frustration level	performance	effort
219	temporal demand	performance	effort	physical demand	temporal demand	temporal demand
220	temporal demand	performance	effort	frustration level	temporal demand	effort
221	physical demand	physical demand	physical demand	physical demand	performance	effort
222	physical demand	performance	physical demand	physical demand	performance	effort
223	physical demand	physical demand	physical demand	physical demand	temporal demand	temporal demand
224	physical demand	performance	effort	frustration level	performance	effort
225	physical demand	physical demand	physical demand	physical demand	performance	temporal demand
226	temporal demand	performance	effort	frustration level	performance	effort
227	temporal demand	physical demand	effort	frustration level	temporal demand	temporal demand
228	physical demand	performance	effort	frustration level	performance	effort
229	physical demand	physical demand	physical demand	physical demand	temporal demand	temporal demand
230	physical demand	performance	effort	physical demand	performance	temporal demand
231	temporal demand	physical demand	physical demand	physical demand	temporal demand	temporal demand
232	temporal demand	performance	effort	frustration level	temporal demand	temporal demand
233	physical demand	physical demand	effort	frustration level	temporal demand	temporal demand
234	temporal demand	performance	effort	frustration level	performance	effort
235	physical demand	physical demand	physical demand	physical demand	temporal demand	temporal demand
236	temporal demand	physical demand	physical demand	frustration level	temporal demand	temporal demand
237	physical demand	performance	physical demand	physical demand	temporal demand	temporal demand
238	physical demand	physical demand	physical demand	physical demand	performance	temporal demand
239	physical demand	physical demand	physical demand	physical demand	performance	temporal demand
240	temporal demand	performance	effort	frustration level	performance	effort
241	physical demand	physical demand	effort	physical demand	performance	effort
242	physical demand	performance	physical demand	frustration level	performance	temporal demand
243	temporal demand	physical demand	effort	physical demand	temporal demand	temporal demand
244	physical demand	performance	physical demand	physical demand	performance	effort
245	physical demand	physical demand	effort	frustration level	performance	temporal demand
246	mental demand	performance	effort	frustration level	performance	effort
247	physical demand	physical demand	physical demand	physical demand	performance	effort
248	physical demand	performance	physical demand	physical demand	performance	effort
249	physical demand	physical demand	physical demand	physical demand	performance	effort
250	physical demand	physical demand	physical demand	physical demand	temporal demand	effort
251	physical demand	performance	effort	frustration level	performance	effort
252	physical demand	physical demand	physical demand	physical demand	temporal demand	effort

ID	physical demand VS temporal demand	physical demand VS performance	physical demand VS effort	physical demand VS frustration level	temporal demand VS performance	temporal demand VS effort
253	physical demand	physical demand	effort	physical demand	temporal demand	temporal demand
254	physical demand	performance	physical demand	frustration level	temporal demand	temporal demand
255	temporal demand	performance	effort	frustration level	performance	effort
256	temporal demand	performance	effort	frustration level	performance	effort
257	physical demand	physical demand	physical demand	physical demand	temporal demand	temporal demand
258	physical demand	physical demand	physical demand	physical demand	temporal demand	temporal demand
259	temporal demand	physical demand	physical demand	physical demand	temporal demand	temporal demand
260	temporal demand	performance	effort	frustration level	performance	effort
261	physical demand	performance	effort	frustration level	performance	effort
262	temporal demand	performance	physical demand	frustration level	performance	temporal demand
263	physical demand	physical demand	effort	physical demand	performance	temporal demand
264	temporal demand	physical demand	physical demand	physical demand	temporal demand	temporal demand
265	physical demand	performance	effort	frustration level	performance	temporal demand
266	physical demand	performance	effort	frustration level	performance	effort
267	physical demand	physical demand	effort	frustration level	temporal demand	effort
268	physical demand	physical demand	physical demand	physical demand	temporal demand	temporal demand
269	physical demand	physical demand	physical demand	physical demand	temporal demand	temporal demand
270	physical demand	physical demand	physical demand	physical demand	performance	effort
271	temporal demand	performance	effort	physical demand	performance	effort
272	temporal demand	physical demand	physical demand	frustration level	temporal demand	temporal demand
273	temporal demand	performance	physical demand	physical demand	temporal demand	temporal demand
274	physical demand	performance	effort	physical demand	temporal demand	effort
275	temporal demand	performance	effort	frustration level	performance	effort
276	temporal demand	performance	effort	frustration level	performance	temporal demand
277	physical demand	physical demand	physical demand	physical demand	performance	effort
278	physical demand	physical demand	physical demand	physical demand	temporal demand	effort
279	physical demand	performance	physical demand	physical demand	performance	temporal demand
280	physical demand	physical demand	physical demand	physical demand	performance	effort
281	temporal demand	performance	effort	frustration level	temporal demand	temporal demand
282	temporal demand	performance	physical demand	physical demand	temporal demand	temporal demand
283	physical demand	physical demand	physical demand	physical demand	temporal demand	temporal demand
284	physical demand	performance	physical demand	frustration level	performance	effort
285	physical demand	performance	physical demand	physical demand	performance	effort
286	physical demand	physical demand	physical demand	physical demand	temporal demand	temporal demand
287	physical demand	physical demand	physical demand	physical demand	temporal demand	temporal demand
288	physical demand	physical demand	effort	physical demand	performance	effort

ID	physical demand VS temporal demand	physical demand VS performance	physical demand VS effort	physical demand VS frustration level	temporal demand VS performance	temporal demand VS effort
289	physical demand	performance	physical demand	frustration level	performance	effort
290	physical demand	performance	physical demand	physical demand	performance	effort
291	temporal demand	physical demand	physical demand	frustration level	temporal demand	temporal demand
292	physical demand	physical demand	physical demand	frustration level	performance	temporal demand
293	physical demand	performance	physical demand	physical demand	performance	effort
294	temporal demand	performance	effort	physical demand	performance	effort
295	physical demand	performance	effort	frustration level	performance	effort
296	physical demand	physical demand	physical demand	physical demand	temporal demand	temporal demand
297	physical demand	physical demand	physical demand	physical demand	temporal demand	temporal demand
298	temporal demand	performance	effort	frustration level	performance	temporal demand
299	physical demand	physical demand	physical demand	physical demand	temporal demand	temporal demand
300	temporal demand	performance	effort	frustration level	performance	effort
301	temporal demand	performance	effort	frustration level	performance	effort
302	physical demand	physical demand	physical demand	physical demand	performance	effort
303	physical demand	physical demand	physical demand	frustration level	temporal demand	temporal demand
304	temporal demand	performance	effort	frustration level	performance	effort
305	physical demand	physical demand	physical demand	physical demand	temporal demand	temporal demand
306	physical demand	performance	effort	frustration level	performance	effort
307	physical demand	physical demand	physical demand	physical demand	performance	temporal demand
308	temporal demand	physical demand	effort	physical demand	temporal demand	effort
309	physical demand	performance	effort	physical demand	temporal demand	temporal demand
310	temporal demand	performance	physical demand	frustration level	temporal demand	temporal demand
311	physical demand	physical demand	effort	frustration level	performance	temporal demand
312	physical demand	physical demand	physical demand	physical demand	performance	effort
313	physical demand	physical demand	physical demand	physical demand	temporal demand	temporal demand
314	physical demand	performance	effort	physical demand	performance	effort
315	temporal demand	performance	physical demand	frustration level	performance	effort
316	physical demand	performance	effort	frustration level	performance	effort
317	temporal demand	performance	effort	physical demand	performance	effort
318	temporal demand	physical demand	physical demand	physical demand	temporal demand	temporal demand
319	temporal demand	performance	effort	frustration level	temporal demand	effort
320	temporal demand	physical demand	effort	physical demand	temporal demand	temporal demand
321	physical demand	performance	physical demand	frustration level	performance	temporal demand
322	temporal demand	performance	effort	frustration level	performance	effort
323	physical demand	physical demand	physical demand	physical demand	temporal demand	temporal demand
324	physical demand	physical demand	physical demand	physical demand	temporal demand	temporal demand

ID	physical demand VS temporal demand	physical demand VS performance	physical demand VS effort	physical demand VS frustration level	temporal demand VS performance	temporal demand VS effort
325	physical demand	physical demand	physical demand	physical demand	temporal demand	effort
326	physical demand	performance	physical demand	physical demand	performance	effort
327	physical demand	performance	physical demand	physical demand	performance	temporal demand
328	temporal demand	performance	effort	physical demand	temporal demand	temporal demand
329	temporal demand	performance	effort	frustration level	performance	effort
330	physical demand	physical demand	physical demand	frustration level	performance	effort
331	physical demand	physical demand	effort	physical demand	performance	effort
332	physical demand	physical demand	physical demand	physical demand	temporal demand	temporal demand
333	temporal demand	performance	effort	physical demand	performance	temporal demand
334	temporal demand	performance	effort	frustration level	performance	effort
335	physical demand	physical demand	physical demand	physical demand	temporal demand	temporal demand
336	temporal demand	performance	effort	frustration level	performance	effort
337	physical demand	physical demand	physical demand	physical demand	performance	temporal demand
338	temporal demand	performance	effort	frustration level	performance	effort
339	temporal demand	performance	effort	frustration level	performance	effort
340	temporal demand	performance	effort	physical demand	performance	effort
341	physical demand	physical demand	physical demand	frustration level	temporal demand	temporal demand
342	temporal demand	performance	effort	frustration level	performance	effort
343	physical demand	physical demand	physical demand	physical demand	temporal demand	temporal demand
344	physical demand	physical demand	physical demand	physical demand	temporal demand	temporal demand
345	physical demand	physical demand	physical demand	physical demand	temporal demand	temporal demand
346	temporal demand	performance	effort	frustration level	performance	temporal demand
347	temporal demand	performance	effort	frustration level	performance	effort
348	temporal demand	performance	effort	frustration level	performance	effort
349	physical demand	performance	physical demand	frustration level	performance	temporal demand
350	temporal demand	physical demand	physical demand	physical demand	temporal demand	temporal demand
351	temporal demand	physical demand	physical demand	frustration level	performance	effort
352	temporal demand	performance	effort	frustration level	temporal demand	temporal demand
353	physical demand	physical demand	effort	physical demand	temporal demand	temporal demand
354	temporal demand	physical demand	physical demand	physical demand	temporal demand	temporal demand
355	physical demand	physical demand	physical demand	physical demand	performance	effort
356	physical demand	physical demand	physical demand	physical demand	temporal demand	temporal demand
357	temporal demand	performance	effort	frustration level	performance	temporal demand
358	physical demand	physical demand	effort	frustration level	temporal demand	effort
359	physical demand	performance	effort	frustration level	performance	effort
360	temporal demand	performance	effort	frustration level	performance	effort

ID	physical demand VS temporal demand	physical demand VS performance	physical demand VS effort	physical demand VS frustration level	temporal demand VS performance	temporal demand VS effort
361	physical demand	performance	effort	frustration level	performance	effort
362	physical demand	physical demand	physical demand	physical demand	temporal demand	effort
363	physical demand	performance	physical demand	physical demand	performance	effort
364	physical demand	physical demand	physical demand	physical demand	performance	effort
365	temporal demand	performance	effort	frustration level	temporal demand	temporal demand
366	physical demand	physical demand	physical demand	frustration level	temporal demand	effort
367	physical demand	physical demand	physical demand	frustration level	temporal demand	effort
368	physical demand	physical demand	physical demand	physical demand	temporal demand	temporal demand
369	physical demand	physical demand	effort	frustration level	performance	temporal demand
370	physical demand	physical demand	physical demand	physical demand	temporal demand	temporal demand
371	temporal demand	performance	effort	frustration level	performance	effort
372	physical demand	physical demand	physical demand	physical demand	temporal demand	temporal demand
373	physical demand	performance	physical demand	physical demand	performance	temporal demand
374	physical demand	physical demand	physical demand	physical demand	performance	temporal demand
375	physical demand	physical demand	physical demand	physical demand	performance	temporal demand
376	temporal demand	performance	effort	frustration level	performance	effort
377	physical demand	physical demand	physical demand	physical demand	temporal demand	temporal demand
378	physical demand	performance	effort	frustration level	performance	temporal demand
379	physical demand	physical demand	physical demand	physical demand	performance	temporal demand
380	temporal demand	physical demand	effort	frustration level	performance	temporal demand
381	physical demand	physical demand	physical demand	physical demand	performance	temporal demand
382	physical demand	physical demand	physical demand	physical demand	performance	temporal demand
383	physical demand	physical demand	physical demand	physical demand	performance	temporal demand
384	physical demand	physical demand	physical demand	physical demand	performance	temporal demand
385	physical demand	performance	effort	frustration level	performance	effort
386	temporal demand	physical demand	physical demand	physical demand	temporal demand	temporal demand
387	temporal demand	performance	effort	frustration level	performance	effort
388	physical demand	physical demand	physical demand	physical demand	performance	effort
389	temporal demand	physical demand	physical demand	frustration level	temporal demand	temporal demand
390	temporal demand	performance	effort	physical demand	performance	effort
391	temporal demand	performance	effort	frustration level	performance	effort
392	physical demand	physical demand	physical demand	physical demand	temporal demand	temporal demand
393	physical demand	physical demand	physical demand	physical demand	performance	effort
394	physical demand	physical demand	physical demand	physical demand	temporal demand	effort
395	temporal demand	performance	effort	physical demand	performance	temporal demand
396	temporal demand	performance	physical demand	physical demand	performance	temporal demand

ID	physical demand VS temporal demand	physical demand VS performance	physical demand VS effort	physical demand VS frustration level	temporal demand VS performance	temporal demand VS effort
397	temporal demand	performance	effort	frustration level	performance	temporal demand
398	temporal demand	performance	effort	frustration level	temporal demand	effort
399	physical demand	performance	physical demand	frustration level	temporal demand	effort
400	temporal demand	physical demand	physical demand	physical demand	temporal demand	effort
401	temporal demand	performance	effort	physical demand	performance	temporal demand
402	physical demand	performance	physical demand	frustration level	performance	effort
403	physical demand	performance	physical demand	physical demand	performance	effort
404	temporal demand	physical demand	effort	physical demand	performance	effort
405	temporal demand	physical demand	physical demand	physical demand	performance	temporal demand
406	temporal demand	performance	effort	frustration level	temporal demand	temporal demand
407	physical demand	performance	physical demand	physical demand	performance	temporal demand
408	temporal demand	performance	effort	frustration level	performance	effort
409	temporal demand	performance	effort	frustration level	temporal demand	temporal demand
410	physical demand	performance	effort	frustration level	temporal demand	effort
411	physical demand	physical demand	physical demand	frustration level	temporal demand	effort
412	temporal demand	performance	effort	frustration level	performance	effort
413	physical demand	physical demand	physical demand	physical demand	temporal demand	temporal demand
414	physical demand	performance	physical demand	frustration level	performance	effort
415	temporal demand	physical demand	physical demand	physical demand	temporal demand	temporal demand
416	temporal demand	performance	physical demand	frustration level	performance	temporal demand
417	temporal demand	performance	effort	frustration level	performance	temporal demand
418	physical demand	performance	effort	physical demand	performance	effort
419	physical demand	performance	effort	frustration level	performance	effort
420	temporal demand	performance	effort	frustration level	performance	effort
421	temporal demand	physical demand	physical demand	physical demand	temporal demand	temporal demand
422	physical demand	physical demand	physical demand	physical demand	performance	temporal demand
423	physical demand	physical demand	physical demand	physical demand	temporal demand	temporal demand
424	physical demand	physical demand	physical demand	physical demand	temporal demand	effort
425	physical demand	physical demand	physical demand	frustration level	temporal demand	effort
426	physical demand	physical demand	physical demand	physical demand	temporal demand	temporal demand
427	physical demand	physical demand	physical demand	physical demand	performance	effort
428	physical demand	physical demand	physical demand	physical demand	temporal demand	temporal demand
429	physical demand	performance	physical demand	physical demand	performance	effort
430	temporal demand	performance	effort	physical demand	temporal demand	effort
431	temporal demand	performance	effort	frustration level	performance	effort
432	physical demand	performance	physical demand	frustration level	temporal demand	effort

ID	physical demand VS temporal demand	physical demand VS performance	physical demand VS effort	physical demand VS frustration level	temporal demand VS performance	temporal demand VS effort
433	temporal demand	physical demand	physical demand	physical demand	temporal demand	temporal demand
434	physical demand	physical demand	physical demand	physical demand	performance	effort
435	physical demand	performance	effort	frustration level	performance	effort
436	temporal demand	performance	effort	frustration level	performance	temporal demand
437	physical demand	physical demand	physical demand	physical demand	temporal demand	temporal demand
438	temporal demand	performance	effort	frustration level	performance	effort
439	physical demand	performance	physical demand	frustration level	performance	effort
440	temporal demand	physical demand	effort	frustration level	temporal demand	temporal demand
441	temporal demand	performance	effort	frustration level	performance	temporal demand
442	temporal demand	physical demand	physical demand	physical demand	temporal demand	temporal demand
443	physical demand	physical demand	physical demand	physical demand	temporal demand	temporal demand
444	physical demand	physical demand	physical demand	physical demand	temporal demand	temporal demand
445	physical demand	performance	effort	frustration level	temporal demand	effort
446	physical demand	physical demand	physical demand	physical demand	temporal demand	temporal demand
447	temporal demand	performance	effort	physical demand	performance	effort
448	physical demand	performance	effort	frustration level	performance	temporal demand
449	physical demand	physical demand	physical demand	physical demand	performance	effort
450	temporal demand	performance	effort	frustration level	performance	effort
451	temporal demand	performance	effort	frustration level	performance	effort
452	physical demand	physical demand	physical demand	physical demand	performance	effort
453	temporal demand	performance	effort	frustration level	performance	effort
454	temporal demand	performance	effort	frustration level	performance	effort
455	physical demand	physical demand	physical demand	physical demand	temporal demand	temporal demand
456	physical demand	performance	physical demand	frustration level	performance	effort
457	temporal demand	performance	effort	frustration level	performance	temporal demand
458	physical demand	physical demand	physical demand	physical demand	temporal demand	temporal demand
459	temporal demand	performance	physical demand	frustration level	temporal demand	temporal demand
460	temporal demand	performance	effort	frustration level	performance	effort
461	physical demand	physical demand	physical demand	physical demand	temporal demand	temporal demand
462	physical demand	performance	effort	frustration level	performance	effort
463	temporal demand	physical demand	effort	physical demand	performance	temporal demand
464	temporal demand	performance	physical demand	frustration level	performance	temporal demand
465	physical demand	performance	effort	frustration level	performance	effort
466	temporal demand	performance	effort	frustration level	performance	effort
467	temporal demand	physical demand	physical demand	physical demand	temporal demand	temporal demand
468	temporal demand	performance	physical demand	physical demand	performance	effort

ID	physical demand VS temporal demand	physical demand VS performance	physical demand VS effort	physical demand VS frustration level	temporal demand VS performance	temporal demand VS effort
469	temporal demand	physical demand	physical demand	physical demand	temporal demand	temporal demand
470	temporal demand	performance	effort	physical demand	performance	effort
471	physical demand	physical demand	physical demand	physical demand	temporal demand	temporal demand
472	physical demand	physical demand	physical demand	physical demand	temporal demand	temporal demand
473	physical demand	physical demand	physical demand	physical demand	temporal demand	temporal demand
474	temporal demand	performance	effort	frustration level	performance	effort
475	temporal demand	performance	effort	frustration level	temporal demand	temporal demand
476	physical demand	physical demand	physical demand	physical demand	performance	temporal demand
477	physical demand	performance	effort	physical demand	temporal demand	effort
478	physical demand	performance	effort	frustration level	performance	effort
479	temporal demand	physical demand	physical demand	physical demand	temporal demand	temporal demand
480	physical demand	physical demand	physical demand	physical demand	temporal demand	temporal demand
481	physical demand	performance	physical demand	physical demand	performance	temporal demand
482	physical demand	physical demand	physical demand	frustration level	performance	effort
483	physical demand	physical demand	physical demand	physical demand	performance	effort
484	temporal demand	performance	effort	frustration level	performance	temporal demand
485	temporal demand	performance	effort	frustration level	temporal demand	temporal demand
486	temporal demand	physical demand	effort	physical demand	temporal demand	effort
487	temporal demand	performance	effort	physical demand	performance	effort
488	temporal demand	performance	effort	frustration level	performance	effort
489	physical demand	physical demand	physical demand	frustration level	performance	effort
490	temporal demand	performance	effort	frustration level	performance	effort
491	physical demand	performance	physical demand	frustration level	performance	effort
492	physical demand	performance	effort	frustration level	temporal demand	effort
493	temporal demand	performance	effort	physical demand	performance	effort
494	temporal demand	physical demand	physical demand	physical demand	temporal demand	temporal demand
495	temporal demand	performance	effort	physical demand	temporal demand	temporal demand
496	physical demand	performance	effort	frustration level	temporal demand	effort
497	temporal demand	performance	physical demand	frustration level	temporal demand	temporal demand
498	physical demand	physical demand	physical demand	frustration level	temporal demand	temporal demand
499	physical demand	performance	effort	frustration level	performance	effort
500	temporal demand	physical demand	effort	physical demand	temporal demand	temporal demand
501	physical demand	physical demand	physical demand	physical demand	performance	effort
502	temporal demand	performance	effort	frustration level	temporal demand	temporal demand
503	physical demand	performance	physical demand	physical demand	performance	temporal demand
504	physical demand	physical demand	physical demand	physical demand	temporal demand	temporal demand

ID	physical demand VS temporal demand	physical demand VS performance	physical demand VS effort	physical demand VS frustration level	temporal demand VS performance	temporal demand VS effort
505	physical demand	physical demand	physical demand	physical demand	temporal demand	temporal demand
506	physical demand	performance	effort	physical demand	performance	effort
507	temporal demand	performance	effort	frustration level	performance	effort
508	temporal demand	performance	effort	frustration level	performance	effort
509	physical demand	performance	effort	frustration level	performance	effort
510	temporal demand	physical demand	effort	physical demand	performance	effort
511	physical demand	physical demand	physical demand	physical demand	temporal demand	temporal demand
512	physical demand	performance	effort	frustration level	temporal demand	effort
513	physical demand	physical demand	physical demand	physical demand	performance	effort
514	temporal demand	physical demand	physical demand	physical demand	temporal demand	temporal demand
515	temporal demand	performance	effort	frustration level	performance	effort
516	temporal demand	physical demand	physical demand	physical demand	temporal demand	temporal demand
517	temporal demand	physical demand	physical demand	frustration level	performance	temporal demand
518	temporal demand	performance	physical demand	frustration level	performance	temporal demand
519	physical demand	physical demand	physical demand	physical demand	performance	temporal demand
520	physical demand	physical demand	physical demand	physical demand	temporal demand	temporal demand
521	physical demand	physical demand	physical demand	physical demand	performance	effort
522	physical demand	physical demand	effort	physical demand	performance	effort
523	physical demand	physical demand	physical demand	frustration level	performance	temporal demand
524	physical demand	performance	effort	physical demand	performance	effort
525	temporal demand	performance	effort	physical demand	temporal demand	effort
526	temporal demand	performance	effort	frustration level	performance	effort
527	temporal demand	performance	effort	frustration level	performance	effort
528	temporal demand	performance	effort	physical demand	temporal demand	temporal demand
529	temporal demand	performance	effort	frustration level	performance	effort
530	physical demand	physical demand	physical demand	physical demand	performance	effort
531	physical demand	physical demand	effort	physical demand	temporal demand	temporal demand
532	physical demand	performance	effort	physical demand	performance	effort
533	physical demand	performance	effort	frustration level	temporal demand	effort
534	physical demand	performance	effort	frustration level	performance	effort
535	temporal demand	performance	physical demand	frustration level	temporal demand	temporal demand
536	physical demand	physical demand	effort	frustration level	performance	effort
537	physical demand	performance	physical demand	physical demand	performance	temporal demand
538	physical demand	physical demand	physical demand	physical demand	temporal demand	temporal demand
539	temporal demand	performance	effort	frustration level	temporal demand	effort
540	temporal demand	physical demand	effort	physical demand	temporal demand	temporal demand

ID	physical demand VS temporal demand	physical demand VS performance	physical demand VS effort	physical demand VS frustration level	temporal demand VS performance	temporal demand VS effort
541	temporal demand	performance	effort	frustration level	performance	effort
542	physical demand	performance	physical demand	frustration level	temporal demand	effort
543	physical demand	physical demand	physical demand	physical demand	temporal demand	temporal demand
544	physical demand	physical demand	physical demand	physical demand	temporal demand	temporal demand
545	temporal demand	performance	effort	frustration level	temporal demand	temporal demand
546	temporal demand	physical demand	physical demand	frustration level	performance	temporal demand
547	physical demand	physical demand	physical demand	physical demand	temporal demand	temporal demand
548	temporal demand	performance	effort	physical demand	performance	effort
549	physical demand	physical demand	physical demand	physical demand	performance	temporal demand
550	physical demand	performance	physical demand	physical demand	temporal demand	temporal demand
551	physical demand	physical demand	physical demand	physical demand	performance	temporal demand
552	physical demand	performance	physical demand	physical demand	performance	temporal demand
553	temporal demand	performance	physical demand	frustration level	performance	temporal demand
554	temporal demand	performance	physical demand	frustration level	temporal demand	temporal demand
555	temporal demand	physical demand	physical demand	frustration level	temporal demand	temporal demand
556	temporal demand	physical demand	physical demand	physical demand	temporal demand	temporal demand
557	physical demand	performance	effort	frustration level	temporal demand	effort
558	temporal demand	performance	physical demand	frustration level	temporal demand	temporal demand
559	physical demand	physical demand	physical demand	frustration level	performance	temporal demand
560	physical demand	physical demand	physical demand	physical demand	performance	temporal demand
561	physical demand	physical demand	physical demand	physical demand	temporal demand	temporal demand
562	physical demand	physical demand	physical demand	frustration level	temporal demand	effort
563	physical demand	performance	physical demand	physical demand	performance	effort
564	physical demand	physical demand	physical demand	physical demand	performance	temporal demand
565	temporal demand	performance	effort	frustration level	performance	effort
566	temporal demand	physical demand	physical demand	physical demand	temporal demand	temporal demand
567	physical demand	performance	effort	frustration level	performance	effort
568	physical demand	physical demand	physical demand	physical demand	performance	effort
569	temporal demand	performance	effort	physical demand	performance	effort
570	temporal demand	performance	physical demand	frustration level	temporal demand	effort
571	physical demand	physical demand	physical demand	frustration level	performance	effort
572	physical demand	performance	physical demand	physical demand	performance	temporal demand
573	physical demand	physical demand	effort	physical demand	performance	effort
574	temporal demand	performance	effort	frustration level	performance	effort
575	physical demand	performance	effort	frustration level	performance	effort
576	temporal demand	performance	effort	frustration level	performance	temporal demand

ID	physical demand VS temporal demand	physical demand VS performance	physical demand VS effort	physical demand VS frustration level	temporal demand VS performance	temporal demand VS effort
577	temporal demand	performance	effort	frustration level	temporal demand	temporal demand
578	temporal demand	performance	effort	physical demand	performance	temporal demand
579	physical demand	performance	physical demand	physical demand	temporal demand	temporal demand
580	physical demand	performance	physical demand	physical demand	performance	temporal demand
581	temporal demand	performance	effort	frustration level	temporal demand	effort
582	physical demand	performance	effort	frustration level	performance	temporal demand
583	physical demand	performance	effort	frustration level	performance	temporal demand
584	temporal demand	performance	physical demand	physical demand	temporal demand	effort
585	physical demand	performance	effort	frustration level	performance	temporal demand
586	physical demand	physical demand	effort	frustration level	temporal demand	temporal demand
587	temporal demand	performance	effort	physical demand	performance	effort
588	physical demand	performance	effort	physical demand	performance	effort
589	physical demand	physical demand	physical demand	physical demand	temporal demand	temporal demand
590	temporal demand	performance	physical demand	physical demand	performance	temporal demand
591	physical demand	physical demand	physical demand	physical demand	temporal demand	temporal demand
592	physical demand	performance	physical demand	frustration level	temporal demand	temporal demand
593	temporal demand	physical demand	physical demand	frustration level	temporal demand	temporal demand
594	temporal demand	performance	effort	frustration level	performance	effort
595	physical demand	physical demand	physical demand	physical demand	temporal demand	temporal demand
596	physical demand	physical demand	physical demand	physical demand	temporal demand	temporal demand
597	physical demand	performance	effort	frustration level	performance	effort
598	temporal demand	physical demand	physical demand	physical demand	temporal demand	temporal demand
599	physical demand	physical demand	effort	frustration level	temporal demand	effort
600	physical demand	physical demand	physical demand	physical demand	performance	effort
601	temporal demand	performance	effort	physical demand	performance	effort
602	physical demand	performance	physical demand	physical demand	performance	temporal demand
603	physical demand	physical demand	physical demand	physical demand	temporal demand	temporal demand
604	temporal demand	performance	physical demand	frustration level	performance	temporal demand
605	physical demand	physical demand	physical demand	physical demand	temporal demand	temporal demand
606	temporal demand	performance	effort	frustration level	temporal demand	temporal demand
607	physical demand	performance	physical demand	physical demand	performance	effort
608	temporal demand	physical demand	physical demand	physical demand	temporal demand	temporal demand
609	physical demand	physical demand	physical demand	physical demand	temporal demand	temporal demand
610	temporal demand	performance	effort	frustration level	performance	effort
611	temporal demand	performance	physical demand	frustration level	temporal demand	effort
612	physical demand	performance	effort	physical demand	performance	effort

ID	physical demand VS temporal demand	physical demand VS performance	physical demand VS effort	physical demand VS frustration level	temporal demand VS performance	temporal demand VS effort
613	temporal demand	performance	effort	frustration level	performance	effort
614	temporal demand	performance	effort	frustration level	performance	effort
615	physical demand	physical demand	physical demand	physical demand	temporal demand	effort
616	physical demand	physical demand	effort	physical demand	performance	effort
617	temporal demand	performance	effort	frustration level	performance	effort
618	temporal demand	performance	effort	frustration level	performance	temporal demand
619	temporal demand	performance	effort	frustration level	performance	effort
620	temporal demand	performance	physical demand	physical demand	performance	effort
621	temporal demand	performance	effort	frustration level	performance	effort
622	physical demand	physical demand	physical demand	physical demand	temporal demand	effort
623	physical demand	physical demand	physical demand	frustration level	performance	effort
624	physical demand	performance	effort	frustration level	performance	effort
625	temporal demand	performance	effort	frustration level	temporal demand	effort
626	physical demand	physical demand	physical demand	physical demand	temporal demand	temporal demand
627	temporal demand	physical demand	physical demand	physical demand	performance	temporal demand
628	physical demand	physical demand	physical demand	physical demand	temporal demand	effort
629	physical demand	physical demand	physical demand	physical demand	performance	effort
630	temporal demand	performance	physical demand	physical demand	temporal demand	temporal demand
631	physical demand	performance	effort	frustration level	performance	effort
632	physical demand	performance	effort	frustration level	performance	effort
633	physical demand	performance	effort	frustration level	performance	effort
634	physical demand	physical demand	physical demand	physical demand	performance	temporal demand
635	physical demand	performance	effort	physical demand	performance	effort
636	temporal demand	performance	effort	frustration level	performance	effort
637	physical demand	physical demand	physical demand	physical demand	temporal demand	temporal demand
638	physical demand	physical demand	effort	physical demand	performance	temporal demand
639	temporal demand	physical demand	physical demand	frustration level	temporal demand	effort
640	physical demand	performance	effort	physical demand	performance	effort
641	physical demand	physical demand	physical demand	physical demand	temporal demand	temporal demand
642	temporal demand	performance	physical demand	frustration level	performance	temporal demand
643	physical demand	performance	effort	physical demand	performance	effort
644	temporal demand	performance	effort	physical demand	performance	effort
645	temporal demand	performance	effort	frustration level	performance	effort
646	temporal demand	physical demand	physical demand	physical demand	temporal demand	temporal demand
647	temporal demand	performance	effort	frustration level	performance	temporal demand
648	physical demand	physical demand	physical demand	physical demand	performance	temporal demand

ID	physical demand VS temporal demand	physical demand VS performance	physical demand VS effort	physical demand VS frustration level	temporal demand VS performance	temporal demand VS effort
649	temporal demand	performance	effort	frustration level	performance	effort
650	temporal demand	physical demand	physical demand	frustration level	temporal demand	temporal demand
651	physical demand	physical demand	physical demand	physical demand	performance	temporal demand
652	physical demand	performance	effort	physical demand	performance	effort
653	physical demand	performance	physical demand	physical demand	performance	temporal demand
654	temporal demand	performance	physical demand	frustration level	performance	temporal demand
655	temporal demand	performance	effort	physical demand	performance	effort
656	physical demand	physical demand	physical demand	physical demand	performance	effort
657	temporal demand	performance	physical demand	physical demand	temporal demand	temporal demand
658	physical demand	physical demand	effort	frustration level	performance	effort
659	temporal demand	performance	physical demand	physical demand	temporal demand	effort
660	temporal demand	performance	effort	frustration level	performance	effort
661	temporal demand	performance	effort	frustration level	performance	effort
662	physical demand	physical demand	physical demand	physical demand	temporal demand	effort
663	temporal demand	performance	physical demand	physical demand	temporal demand	effort
664	physical demand	performance	effort	frustration level	performance	effort
665	temporal demand	performance	physical demand	physical demand	performance	effort
666	physical demand	physical demand	physical demand	physical demand	performance	effort
667	temporal demand	performance	physical demand	physical demand	temporal demand	temporal demand
668	temporal demand	physical demand	physical demand	physical demand	temporal demand	temporal demand
669	physical demand	physical demand	physical demand	physical demand	temporal demand	temporal demand
670	physical demand	performance	effort	frustration level	performance	effort
671	temporal demand	performance	effort	physical demand	temporal demand	temporal demand
672	physical demand	physical demand	physical demand	physical demand	performance	effort
673	temporal demand	performance	effort	frustration level	performance	effort
674	temporal demand	physical demand	physical demand	physical demand	temporal demand	temporal demand
675	physical demand	physical demand	physical demand	physical demand	temporal demand	temporal demand
676	temporal demand	physical demand	physical demand	physical demand	performance	temporal demand
677	temporal demand	performance	effort	frustration level	performance	temporal demand
678	physical demand	performance	effort	frustration level	performance	effort
679	physical demand	physical demand	physical demand	physical demand	temporal demand	temporal demand
680	temporal demand	performance	effort	frustration level	performance	effort
681	temporal demand	performance	effort	frustration level	performance	effort
682	physical demand	physical demand	physical demand	frustration level	temporal demand	effort
683	temporal demand	performance	effort	frustration level	performance	effort
684	physical demand	performance	effort	physical demand	performance	effort

ID	physical demand VS temporal demand	physical demand VS performance	physical demand VS effort	physical demand VS frustration level	temporal demand VS performance	temporal demand VS effort
685	temporal demand	performance	effort	frustration level	performance	effort
686	physical demand	physical demand	physical demand	physical demand	temporal demand	temporal demand
687	physical demand	performance	effort	frustration level	performance	effort
688	physical demand	performance	effort	physical demand	performance	effort
689	physical demand	performance	physical demand	physical demand	performance	temporal demand
690	temporal demand	performance	effort	frustration level	performance	effort
691	temporal demand	physical demand	effort	physical demand	temporal demand	temporal demand
692	physical demand	performance	physical demand	physical demand	performance	temporal demand
693	temporal demand	performance	effort	frustration level	performance	temporal demand
694	temporal demand	performance	physical demand	physical demand	temporal demand	temporal demand
695	physical demand	physical demand	physical demand	physical demand	temporal demand	effort
696	physical demand	physical demand	physical demand	physical demand	performance	temporal demand
697	physical demand	physical demand	physical demand	physical demand	performance	temporal demand
698	physical demand	physical demand	physical demand	physical demand	temporal demand	effort
699	physical demand	physical demand	physical demand	physical demand	temporal demand	temporal demand
700	physical demand	physical demand	physical demand	physical demand	temporal demand	temporal demand
701	temporal demand	performance	effort	frustration level	performance	effort
702	physical demand	physical demand	physical demand	frustration level	temporal demand	temporal demand
703	physical demand	physical demand	physical demand	physical demand	temporal demand	temporal demand
704	temporal demand	performance	effort	frustration level	performance	effort
705	temporal demand	performance	effort	frustration level	performance	effort
706	physical demand	performance	physical demand	physical demand	performance	temporal demand
707	temporal demand	performance	physical demand	physical demand	performance	effort
708	physical demand	physical demand	physical demand	physical demand	performance	effort
709	physical demand	physical demand	physical demand	physical demand	temporal demand	temporal demand
710	physical demand	physical demand	effort	frustration level	performance	effort
711	temporal demand	performance	effort	frustration level	performance	effort
712	physical demand	physical demand	physical demand	frustration level	temporal demand	temporal demand
713	physical demand	physical demand	physical demand	physical demand	temporal demand	temporal demand
714	physical demand	physical demand	physical demand	physical demand	temporal demand	temporal demand
715	temporal demand	performance	effort	frustration level	performance	effort
716	physical demand	performance	physical demand	frustration level	performance	temporal demand
717	temporal demand	performance	effort	frustration level	performance	effort
718	physical demand	physical demand	physical demand	physical demand	performance	effort
719	temporal demand	performance	physical demand	frustration level	performance	temporal demand
720	temporal demand	performance	effort	physical demand	temporal demand	effort

ID	physical demand VS temporal demand	physical demand VS performance	physical demand VS effort	physical demand VS frustration level	temporal demand VS performance	temporal demand VS effort
721	physical demand	performance	physical demand	physical demand	temporal demand	temporal demand
722	physical demand	performance	effort	physical demand	performance	temporal demand
723	temporal demand	performance	effort	frustration level	performance	effort
724	physical demand	physical demand	effort	physical demand	temporal demand	temporal demand
725	physical demand	physical demand	physical demand	physical demand	temporal demand	temporal demand
726	physical demand	physical demand	physical demand	physical demand	temporal demand	temporal demand
727	physical demand	physical demand	physical demand	physical demand	temporal demand	temporal demand
728	temporal demand	physical demand	physical demand	physical demand	temporal demand	temporal demand
729	physical demand	physical demand	physical demand	physical demand	temporal demand	temporal demand
730	temporal demand	performance	physical demand	physical demand	performance	effort
731	physical demand	physical demand	physical demand	physical demand	temporal demand	temporal demand
732	physical demand	physical demand	physical demand	physical demand	temporal demand	temporal demand
733	physical demand	physical demand	physical demand	physical demand	performance	effort
734	physical demand	performance	effort	frustration level	performance	effort
735	physical demand	physical demand	physical demand	physical demand	temporal demand	temporal demand
736	temporal demand	performance	physical demand	physical demand	performance	effort
737	physical demand	performance	physical demand	frustration level	temporal demand	temporal demand
738	physical demand	physical demand	physical demand	frustration level	performance	temporal demand
739	temporal demand	performance	effort	frustration level	performance	effort
740	temporal demand	performance	effort	physical demand	temporal demand	effort
741	physical demand	performance	effort	frustration level	performance	temporal demand
742	temporal demand	performance	effort	frustration level	performance	effort
743	physical demand	physical demand	physical demand	physical demand	performance	effort
744	physical demand	physical demand	physical demand	frustration level	temporal demand	temporal demand
745	temporal demand	performance	physical demand	frustration level	temporal demand	temporal demand
746	physical demand	performance	physical demand	physical demand	performance	effort
747	physical demand	physical demand	physical demand	physical demand	performance	effort
748	physical demand	performance	physical demand	physical demand	temporal demand	temporal demand
749	temporal demand	performance	effort	frustration level	performance	temporal demand
750	physical demand	performance	effort	physical demand	performance	effort
751	physical demand	performance	effort	frustration level	performance	effort
752	physical demand	performance	effort	frustration level	performance	effort
753	temporal demand	performance	effort	frustration level	performance	effort
754	physical demand	performance	effort	frustration level	performance	effort
755	physical demand	physical demand	physical demand	physical demand	temporal demand	effort
756	physical demand	physical demand	physical demand	physical demand	temporal demand	temporal demand

ID	physical demand VS temporal demand	physical demand VS performance	physical demand VS effort	physical demand VS frustration level	temporal demand VS performance	temporal demand VS effort
757	temporal demand	performance	physical demand	physical demand	performance	effort
758	temporal demand	performance	effort	physical demand	performance	effort
759	physical demand	physical demand	effort	physical demand	temporal demand	temporal demand
760	physical demand	physical demand	physical demand	physical demand	temporal demand	temporal demand
761	physical demand	performance	physical demand	physical demand	temporal demand	temporal demand
762	physical demand	physical demand	physical demand	physical demand	temporal demand	temporal demand
763	temporal demand	physical demand	physical demand	physical demand	temporal demand	temporal demand
764	temporal demand	performance	effort	frustration level	performance	temporal demand
765	temporal demand	physical demand	physical demand	physical demand	temporal demand	temporal demand
766	temporal demand	performance	physical demand	physical demand	performance	temporal demand
767	temporal demand	performance	effort	frustration level	performance	effort
768	physical demand	physical demand	physical demand	frustration level	performance	temporal demand
769	physical demand	physical demand	effort	physical demand	temporal demand	temporal demand
770	temporal demand	performance	physical demand	frustration level	performance	temporal demand
771	temporal demand	performance	physical demand	frustration level	temporal demand	temporal demand
772	temporal demand	performance	effort	frustration level	performance	temporal demand
773	physical demand	physical demand	physical demand	physical demand	performance	effort
774	temporal demand	performance	physical demand	physical demand	performance	temporal demand
775	temporal demand	physical demand	physical demand	physical demand	temporal demand	temporal demand
776	temporal demand	physical demand	effort	frustration level	performance	effort
777	physical demand	physical demand	physical demand	physical demand	temporal demand	temporal demand
778	physical demand	physical demand	physical demand	physical demand	temporal demand	effort
779	physical demand	physical demand	physical demand	physical demand	temporal demand	temporal demand
780	temporal demand	performance	effort	frustration level	performance	effort
781	temporal demand	performance	effort	frustration level	performance	effort
782	temporal demand	physical demand	effort	frustration level	performance	effort
783	physical demand	performance	physical demand	frustration level	performance	temporal demand
784	physical demand	performance	effort	frustration level	performance	effort
785	physical demand	physical demand	physical demand	physical demand	temporal demand	effort
786	temporal demand	performance	effort	frustration level	performance	effort
787	physical demand	performance	effort	frustration level	performance	effort
788	temporal demand	performance	physical demand	physical demand	temporal demand	temporal demand
789	temporal demand	performance	physical demand	physical demand	temporal demand	effort
790	physical demand	performance	effort	frustration level	performance	effort
791	temporal demand	physical demand	physical demand	physical demand	temporal demand	temporal demand
792	physical demand	physical demand	physical demand	physical demand	performance	effort

ID	physical demand VS temporal demand	physical demand VS performance	physical demand VS effort	physical demand VS frustration level	temporal demand VS performance	temporal demand VS effort
793	physical demand	performance	physical demand	frustration level	performance	effort
794	physical demand	physical demand	physical demand	frustration level	temporal demand	temporal demand
795	temporal demand	performance	effort	frustration level	performance	effort
796	temporal demand	performance	physical demand	physical demand	temporal demand	temporal demand
797	physical demand	performance	effort	frustration level	performance	temporal demand
798	temporal demand	performance	effort	frustration level	performance	effort
799	physical demand	physical demand	physical demand	frustration level	performance	effort
800	physical demand	performance	physical demand	physical demand	temporal demand	temporal demand
801	temporal demand	physical demand	physical demand	physical demand	temporal demand	temporal demand
802	temporal demand	physical demand	physical demand	frustration level	temporal demand	temporal demand
803	temporal demand	physical demand	physical demand	physical demand	temporal demand	temporal demand
804	physical demand	performance	effort	physical demand	temporal demand	effort
805	physical demand	physical demand	physical demand	physical demand	performance	temporal demand
806	temporal demand	performance	effort	frustration level	performance	effort
807	temporal demand	performance	effort	frustration level	performance	effort
808	temporal demand	performance	effort	frustration level	performance	effort
809	physical demand	physical demand	physical demand	physical demand	performance	temporal demand
810	physical demand	physical demand	physical demand	physical demand	temporal demand	temporal demand
811	physical demand	physical demand	physical demand	frustration level	temporal demand	temporal demand
812	temporal demand	physical demand	physical demand	physical demand	temporal demand	temporal demand
813	temporal demand	performance	effort	frustration level	performance	effort
814	physical demand	physical demand	physical demand	physical demand	performance	effort
815	temporal demand	performance	effort	frustration level	performance	temporal demand
816	temporal demand	performance	effort	frustration level	performance	effort
817	temporal demand	performance	physical demand	frustration level	performance	effort
818	temporal demand	performance	effort	frustration level	performance	effort
819	temporal demand	performance	effort	frustration level	performance	effort
820	temporal demand	physical demand	physical demand	frustration level	performance	effort
821	temporal demand	physical demand	physical demand	physical demand	temporal demand	temporal demand
822	temporal demand	performance	physical demand	physical demand	temporal demand	temporal demand
823	temporal demand	performance	effort	physical demand	temporal demand	effort
824	physical demand	physical demand	physical demand	frustration level	temporal demand	temporal demand
825	physical demand	physical demand	physical demand	physical demand	temporal demand	effort
826	physical demand	performance	physical demand	frustration level	temporal demand	effort
827	temporal demand	physical demand	physical demand	physical demand	temporal demand	temporal demand
828	physical demand	physical demand	physical demand	physical demand	temporal demand	effort

ID	physical demand VS temporal demand	physical demand VS performance	physical demand VS effort	physical demand VS frustration level	temporal demand VS performance	temporal demand VS effort
829	physical demand	physical demand	physical demand	physical demand	performance	effort
830	temporal demand	performance	effort	frustration level	temporal demand	temporal demand
831	physical demand	physical demand	physical demand	physical demand	temporal demand	temporal demand
832	temporal demand	physical demand	physical demand	physical demand	temporal demand	temporal demand
833	physical demand	performance	physical demand	frustration level	temporal demand	effort
834	physical demand	physical demand	physical demand	physical demand	temporal demand	effort
835	temporal demand	performance	physical demand	physical demand	performance	effort
836	physical demand	physical demand	effort	physical demand	performance	temporal demand
837	temporal demand	performance	physical demand	frustration level	performance	effort
838	physical demand	physical demand	physical demand	physical demand	temporal demand	effort
839	physical demand	physical demand	physical demand	physical demand	temporal demand	effort
840	physical demand	physical demand	physical demand	physical demand	performance	effort
841	temporal demand	performance	effort	frustration level	performance	effort
842	physical demand	physical demand	physical demand	physical demand	temporal demand	temporal demand
843	temporal demand	performance	effort	frustration level	performance	effort
844	temporal demand	performance	effort	frustration level	performance	temporal demand
845	temporal demand	performance	physical demand	frustration level	performance	temporal demand
846	temporal demand	performance	effort	frustration level	temporal demand	temporal demand
847	physical demand	physical demand	physical demand	physical demand	temporal demand	temporal demand
848	physical demand	physical demand	physical demand	physical demand	temporal demand	temporal demand
849	physical demand	physical demand	physical demand	physical demand	performance	temporal demand
850	physical demand	performance	effort	frustration level	performance	effort
851	physical demand	performance	effort	physical demand	performance	temporal demand
852	temporal demand	performance	effort	frustration level	performance	effort
853	physical demand	performance	physical demand	frustration level	performance	temporal demand
854	physical demand	physical demand	physical demand	physical demand	performance	temporal demand
855	physical demand	physical demand	physical demand	physical demand	temporal demand	temporal demand
856	physical demand	physical demand	physical demand	physical demand	temporal demand	temporal demand
857	physical demand	physical demand	physical demand	physical demand	performance	temporal demand
858	temporal demand	performance	effort	frustration level	performance	effort
859	temporal demand	physical demand	effort	frustration level	performance	effort
860	physical demand	performance	effort	physical demand	performance	effort
861	temporal demand	performance	effort	frustration level	performance	effort
862	temporal demand	performance	effort	frustration level	performance	effort
863	physical demand	performance	effort	frustration level	performance	effort
864	physical demand	performance	physical demand	frustration level	performance	temporal demand

ID	physical demand VS temporal demand	physical demand VS performance	physical demand VS effort	physical demand VS frustration level	temporal demand VS performance	temporal demand VS effort
865	temporal demand	physical demand	physical demand	physical demand	temporal demand	temporal demand
866	physical demand	physical demand	physical demand	physical demand	temporal demand	effort
867	temporal demand	performance	effort	frustration level	performance	effort
868	physical demand	physical demand	physical demand	frustration level	performance	temporal demand
869	physical demand	physical demand	physical demand	physical demand	temporal demand	temporal demand
870	temporal demand	performance	effort	frustration level	performance	effort
871	physical demand	performance	physical demand	frustration level	performance	temporal demand
872	temporal demand	performance	effort	frustration level	temporal demand	temporal demand
873	physical demand	physical demand	physical demand	physical demand	performance	effort
874	temporal demand	performance	effort	frustration level	performance	temporal demand
875	temporal demand	performance	physical demand	physical demand	temporal demand	temporal demand
876	physical demand	performance	physical demand	physical demand	performance	effort
877	physical demand	physical demand	physical demand	physical demand	performance	effort
878	temporal demand	performance	effort	frustration level	temporal demand	effort
879	physical demand	performance	effort	frustration level	performance	effort
880	physical demand	physical demand	physical demand	physical demand	performance	effort
881	physical demand	physical demand	physical demand	physical demand	temporal demand	temporal demand
882	temporal demand	performance	effort	frustration level	performance	effort
883	physical demand	performance	physical demand	physical demand	temporal demand	temporal demand
884	temporal demand	performance	effort	physical demand	temporal demand	temporal demand
885	temporal demand	performance	physical demand	frustration level	temporal demand	effort
886	physical demand	performance	physical demand	frustration level	temporal demand	temporal demand
887	physical demand	physical demand	physical demand	physical demand	performance	effort
888	physical demand	performance	physical demand	frustration level	performance	temporal demand
889	temporal demand	performance	physical demand	frustration level	temporal demand	temporal demand
890	temporal demand	performance	effort	physical demand	performance	temporal demand
891	temporal demand	performance	physical demand	physical demand	performance	temporal demand
892	physical demand	physical demand	physical demand	physical demand	performance	effort
893	temporal demand	performance	physical demand	frustration level	performance	effort
894	temporal demand	physical demand	effort	frustration level	temporal demand	effort
895	temporal demand	performance	effort	frustration level	performance	temporal demand
896	temporal demand	physical demand	physical demand	physical demand	temporal demand	temporal demand
897	temporal demand	performance	effort	frustration level	performance	effort
898	physical demand	physical demand	physical demand	physical demand	temporal demand	temporal demand
899	temporal demand	performance	effort	frustration level	performance	effort
900	physical demand	performance	physical demand	frustration level	temporal demand	effort

ID	physical demand VS temporal demand	physical demand VS performance	physical demand VS effort	physical demand VS frustration level	temporal demand VS performance	temporal demand VS effort
901	physical demand	performance	effort	frustration level	performance	effort
902	temporal demand	physical demand	physical demand	physical demand	performance	effort
903	temporal demand	performance	physical demand	frustration level	performance	temporal demand
904	temporal demand	performance	physical demand	physical demand	performance	effort
905	temporal demand	performance	effort	frustration level	temporal demand	effort
906	temporal demand	performance	physical demand	frustration level	performance	effort
907	physical demand	physical demand	effort	frustration level	temporal demand	temporal demand
908	temporal demand	performance	effort	frustration level	performance	temporal demand
909	physical demand	performance	physical demand	frustration level	performance	effort
910	temporal demand	performance	effort	frustration level	temporal demand	temporal demand
911	temporal demand	performance	effort	frustration level	performance	effort
912	temporal demand	performance	effort	frustration level	performance	effort
913	temporal demand	physical demand	physical demand	frustration level	temporal demand	temporal demand
914	temporal demand	performance	effort	frustration level	performance	effort
915	temporal demand	performance	effort	physical demand	performance	effort
916	temporal demand	performance	effort	frustration level	performance	effort
917	physical demand	performance	effort	physical demand	temporal demand	effort
918	temporal demand	performance	effort	frustration level	performance	temporal demand
919	temporal demand	physical demand	physical demand	physical demand	temporal demand	temporal demand
920	temporal demand	performance	physical demand	physical demand	temporal demand	temporal demand
921	temporal demand	performance	effort	frustration level	performance	temporal demand
922	physical demand	physical demand	physical demand	physical demand	temporal demand	temporal demand
923	physical demand	physical demand	physical demand	physical demand	temporal demand	temporal demand
924	temporal demand	performance	effort	physical demand	performance	temporal demand
925	physical demand	physical demand	physical demand	physical demand	performance	effort
926	physical demand	performance	physical demand	frustration level	performance	temporal demand
927	temporal demand	performance	physical demand	frustration level	temporal demand	effort
928	temporal demand	performance	effort	physical demand	temporal demand	temporal demand
929	temporal demand	performance	effort	physical demand	temporal demand	temporal demand
930	temporal demand	performance	physical demand	physical demand	performance	temporal demand
931	physical demand	physical demand	physical demand	physical demand	temporal demand	temporal demand
932	temporal demand	physical demand	physical demand	physical demand	temporal demand	temporal demand
933	temporal demand	physical demand	effort	physical demand	temporal demand	temporal demand
934	physical demand	performance	physical demand	frustration level	performance	effort
935	physical demand	physical demand	physical demand	frustration level	temporal demand	effort
936	temporal demand	performance	effort	frustration level	performance	effort

ID	physical demand VS temporal demand	physical demand VS performance	physical demand VS effort	physical demand VS frustration level	temporal demand VS performance	temporal demand VS effort
937	physical demand	physical demand	physical demand	physical demand	temporal demand	temporal demand
938	temporal demand	performance	physical demand	frustration level	temporal demand	temporal demand
939	temporal demand	performance	effort	frustration level	performance	effort
940	physical demand	physical demand	physical demand	physical demand	temporal demand	temporal demand
941	physical demand	performance	effort	frustration level	performance	effort
942	temporal demand	performance	effort	physical demand	temporal demand	temporal demand
943	temporal demand	performance	physical demand	frustration level	performance	temporal demand
944	temporal demand	performance	effort	frustration level	performance	effort
945	temporal demand	performance	physical demand	physical demand	temporal demand	temporal demand
946	physical demand	performance	effort	frustration level	temporal demand	effort
947	physical demand	performance	effort	physical demand	performance	effort
948	temporal demand	performance	physical demand	physical demand	temporal demand	temporal demand
949	physical demand	physical demand	physical demand	frustration level	temporal demand	temporal demand
950	physical demand	performance	effort	physical demand	performance	effort
951	physical demand	performance	physical demand	frustration level	performance	effort
952	physical demand	performance	effort	physical demand	performance	effort
953	temporal demand	performance	effort	frustration level	performance	effort
954	temporal demand	performance	effort	frustration level	performance	temporal demand
955	physical demand	physical demand	physical demand	physical demand	temporal demand	temporal demand
956	physical demand	physical demand	physical demand	frustration level	performance	temporal demand
957	temporal demand	performance	effort	physical demand	temporal demand	temporal demand
958	physical demand	performance	effort	physical demand	performance	effort
959	temporal demand	physical demand	physical demand	physical demand	temporal demand	temporal demand
960	physical demand	physical demand	effort	frustration level	temporal demand	temporal demand
961	physical demand	physical demand	physical demand	physical demand	temporal demand	temporal demand
962	physical demand	performance	physical demand	physical demand	performance	temporal demand
963	temporal demand	physical demand	effort	frustration level	temporal demand	effort
964	physical demand	physical demand	physical demand	physical demand	performance	temporal demand
965	temporal demand	performance	effort	frustration level	temporal demand	temporal demand
966	temporal demand	performance	effort	frustration level	temporal demand	effort
967	temporal demand	performance	physical demand	frustration level	temporal demand	temporal demand
968	physical demand	physical demand	physical demand	frustration level	temporal demand	effort
969	temporal demand	performance	effort	physical demand	performance	effort
970	temporal demand	performance	physical demand	physical demand	temporal demand	temporal demand
971	physical demand	physical demand	physical demand	frustration level	temporal demand	temporal demand
972	physical demand	performance	effort	physical demand	performance	temporal demand

ID	physical demand VS temporal demand	physical demand VS performance	physical demand VS effort	physical demand VS frustration level	temporal demand VS performance	temporal demand VS effort
973	temporal demand	physical demand	effort	frustration level	temporal demand	effort
974	physical demand	performance	physical demand	physical demand	performance	temporal demand
975	temporal demand	performance	effort	frustration level	performance	effort
976	temporal demand	performance	effort	frustration level	temporal demand	effort
977	physical demand	physical demand	physical demand	physical demand	temporal demand	temporal demand
978	temporal demand	performance	effort	frustration level	temporal demand	temporal demand
979	temporal demand	performance	effort	frustration level	temporal demand	effort
980	physical demand	performance	physical demand	frustration level	performance	temporal demand
981	temporal demand	performance	effort	frustration level	performance	effort
982	temporal demand	performance	effort	frustration level	performance	temporal demand
983	physical demand	physical demand	physical demand	frustration level	performance	effort
984	physical demand	physical demand	effort	frustration level	temporal demand	temporal demand
985	physical demand	performance	physical demand	physical demand	performance	temporal demand
986	physical demand	physical demand	physical demand	physical demand	performance	effort
987	temporal demand	physical demand	physical demand	frustration level	temporal demand	temporal demand
988	temporal demand	physical demand	effort	physical demand	performance	temporal demand
989	temporal demand	performance	effort	frustration level	performance	temporal demand
990	temporal demand	physical demand	physical demand	physical demand	temporal demand	temporal demand
991	temporal demand	performance	effort	physical demand	temporal demand	effort
992	temporal demand	performance	effort	frustration level	temporal demand	temporal demand
993	temporal demand	performance	physical demand	frustration level	performance	temporal demand
994	temporal demand	physical demand	physical demand	physical demand	temporal demand	temporal demand
995	temporal demand	physical demand	effort	physical demand	performance	temporal demand
996	temporal demand	physical demand	physical demand	frustration level	temporal demand	temporal demand
997	physical demand	physical demand	effort	frustration level	temporal demand	temporal demand
998	physical demand	physical demand	physical demand	frustration level	performance	temporal demand
999	temporal demand	physical demand	effort	physical demand	temporal demand	effort
1000	physical demand	physical demand	physical demand	physical demand	temporal demand	temporal demand
1001	physical demand	performance	physical demand	frustration level	temporal demand	temporal demand
1002	physical demand	physical demand	physical demand	physical demand	temporal demand	temporal demand
1003	temporal demand	performance	physical demand	frustration level	performance	effort
1004	temporal demand	performance	effort	frustration level	performance	effort
