# Supplementary material for: Factoring and correlation in sleep, fatigue and mental workload of clinical first-line nurses in the post-pandemic era of COVID-19: A multi-center cross-sectional study
Source: Front Psychiatry. 2022 Aug 25;13:963419. doi: 10.3389/fpsyt.2022.963419 (PMC9452657; doi:10.3389/fpsyt.2022.963419)
Supplement: Supplementary file 3 [file Data_Sheet_3.PDF]

ID	temporal demand<br>VS<br>frustration level	effort<br>VS<br>performance	performance<br>VS<br>frustration level	effort<br>VS<br>frustration level
1	temporal demand	performance	performance	effort
2	frustration level	performance	performance	frustration level
3	frustration level	performance	performance	frustration level
4	temporal demand	performance	performance	frustration level
5	frustration level	performance	frustration level	frustration level
6	frustration level	performance	performance	frustration level
7	frustration level	effort	performance	effort
8	temporal demand	performance	performance	effort
9	temporal demand	performance	performance	frustration level
10	frustration level	performance	performance	effort
11	frustration level	performance	performance	frustration level
12	temporal demand	performance	performance	frustration level
13	frustration level	performance	performance	frustration level
14	temporal demand	performance	frustration level	frustration level
15	temporal demand	performance	performance	effort
16	temporal demand	performance	frustration level	frustration level
17	frustration level	effort	performance	effort
18	temporal demand	performance	performance	effort
19	temporal demand	effort	frustration level	effort
20	temporal demand	effort	frustration level	effort
21	frustration level	effort	frustration level	effort
22	temporal demand	effort	performance	effort
23	temporal demand	performance	performance	effort
24	temporal demand	performance	performance	effort
25	temporal demand	performance	performance	effort
26	temporal demand	performance	performance	effort
27	temporal demand	effort	performance	effort
28	temporal demand	performance	performance	frustration level
29	temporal demand	performance	performance	frustration level
30	frustration level	effort	performance	frustration level
31	temporal demand	performance	performance	frustration level
32	temporal demand	performance	frustration level	frustration level
33	temporal demand	performance	performance	frustration level
34	frustration level	performance	frustration level	frustration level
35	frustration level	effort	performance	effort
36	temporal demand	performance	performance	effort

ID	temporal demand VS frustration level	effort VS performance	performance VS frustration level	effort VS frustration level
37	temporal demand	effort	performance	effort
38	temporal demand	effort	frustration level	effort
39	frustration level	performance	performance	frustration level
40	temporal demand	effort	frustration level	frustration level
41	frustration level	performance	performance	effort
42	temporal demand	performance	performance	frustration level
43	temporal demand	performance	performance	frustration level
44	frustration level	performance	performance	effort
45	temporal demand	effort	performance	effort
46	temporal demand	effort	performance	effort
47	temporal demand	performance	performance	effort
48	frustration level	performance	frustration level	frustration level
49	frustration level	effort	performance	effort
50	temporal demand	effort	performance	effort
51	temporal demand	performance	performance	effort
52	temporal demand	effort	performance	frustration level
53	temporal demand	performance	performance	effort
54	temporal demand	effort	frustration level	effort
55	frustration level	performance	frustration level	frustration level
56	frustration level	performance	frustration level	frustration level
57	frustration level	effort	frustration level	frustration level
58	frustration level	performance	performance	frustration level
59	frustration level	effort	performance	effort
60	temporal demand	performance	performance	frustration level
61	frustration level	effort	performance	effort
62	temporal demand	effort	performance	frustration level
63	frustration level	performance	performance	frustration level
64	temporal demand	performance	performance	effort
65	temporal demand	performance	performance	frustration level
66	temporal demand	effort	performance	effort
67	temporal demand	effort	frustration level	frustration level
68	frustration level	performance	performance	frustration level
69	temporal demand	performance	performance	effort
70	frustration level	performance	frustration level	frustration level
71	temporal demand	performance	performance	effort
72	temporal demand	performance	performance	effort

ID	temporal demand VS frustration level	effort VS performance	performance VS frustration level	effort VS frustration level
73	temporal demand	performance	performance	effort
74	temporal demand	performance	performance	frustration level
75	temporal demand	effort	performance	effort
76	frustration level	performance	frustration level	frustration level
77	frustration level	performance	frustration level	frustration level
78	temporal demand	performance	performance	effort
79	frustration level	effort	frustration level	frustration level
80	frustration level	performance	performance	frustration level
81	temporal demand	performance	performance	frustration level
82	temporal demand	effort	frustration level	effort
83	temporal demand	performance	performance	effort
84	temporal demand	effort	frustration level	effort
85	frustration level	effort	performance	effort
86	temporal demand	performance	performance	frustration level
87	frustration level	performance	frustration level	effort
88	temporal demand	performance	performance	effort
89	frustration level	effort	frustration level	frustration level
90	frustration level	effort	performance	frustration level
91	frustration level	performance	performance	frustration level
92	temporal demand	performance	frustration level	frustration level
93	temporal demand	performance	performance	effort
94	temporal demand	performance	performance	effort
95	frustration level	performance	performance	frustration level
96	temporal demand	performance	performance	effort
97	temporal demand	performance	frustration level	effort
98	frustration level	performance	performance	frustration level
99	frustration level	performance	frustration level	frustration level
100	temporal demand	performance	performance	effort
101	frustration level	effort	frustration level	effort
102	temporal demand	performance	performance	frustration level
103	frustration level	performance	performance	effort
104	temporal demand	performance	performance	effort
105	frustration level	performance	performance	effort
106	temporal demand	performance	performance	frustration level
107	frustration level	effort	performance	frustration level
108	temporal demand	performance	performance	effort

ID	temporal demand VS frustration level	effort VS performance	performance VS frustration level	effort VS frustration level
109	frustration level	performance	frustration level	frustration level
110	frustration level	effort	performance	frustration level
111	temporal demand	performance	performance	frustration level
112	frustration level	performance	performance	effort
113	frustration level	performance	performance	effort
114	frustration level	effort	performance	effort
115	frustration level	effort	frustration level	frustration level
116	temporal demand	performance	performance	effort
117	temporal demand	performance	performance	frustration level
118	frustration level	effort	frustration level	effort
119	frustration level	effort	performance	effort
120	frustration level	performance	frustration level	effort
121	frustration level	performance	performance	effort
122	frustration level	performance	frustration level	effort
123	temporal demand	effort	performance	frustration level
124	frustration level	performance	frustration level	frustration level
125	temporal demand	performance	frustration level	frustration level
126	temporal demand	performance	performance	effort
127	temporal demand	effort	performance	effort
128	temporal demand	effort	performance	frustration level
129	temporal demand	effort	performance	effort
130	temporal demand	performance	performance	effort
131	temporal demand	performance	performance	frustration level
132	temporal demand	performance	performance	frustration level
133	frustration level	performance	performance	effort
134	frustration level	performance	frustration level	effort
135	frustration level	performance	performance	effort
136	temporal demand	performance	performance	effort
137	frustration level	performance	frustration level	frustration level
138	temporal demand	effort	performance	effort
139	temporal demand	performance	performance	effort
140	temporal demand	effort	performance	effort
141	temporal demand	performance	performance	frustration level
142	frustration level	performance	performance	frustration level
143	frustration level	effort	performance	effort
144	temporal demand	performance	frustration level	effort

ID	temporal demand VS frustration level	effort VS performance	performance VS frustration level	effort VS frustration level
145	frustration level	effort	frustration level	effort
146	frustration level	performance	performance	frustration level
147	temporal demand	performance	performance	frustration level
148	temporal demand	performance	performance	frustration level
149	frustration level	performance	performance	frustration level
150	temporal demand	performance	performance	frustration level
151	temporal demand	performance	performance	effort
152	frustration level	performance	frustration level	frustration level
153	temporal demand	performance	performance	effort
154	frustration level	performance	performance	frustration level
155	temporal demand	performance	performance	effort
156	temporal demand	performance	performance	frustration level
157	temporal demand	performance	performance	effort
158	temporal demand	effort	performance	effort
159	temporal demand	performance	performance	frustration level
160	frustration level	performance	performance	frustration level
161	temporal demand	performance	performance	effort
162	temporal demand	performance	performance	frustration level
163	frustration level	performance	performance	frustration level
164	frustration level	performance	frustration level	frustration level
165	frustration level	performance	frustration level	effort
166	temporal demand	effort	performance	effort
167	frustration level	effort	performance	effort
168	frustration level	performance	performance	frustration level
169	frustration level	effort	performance	effort
170	temporal demand	effort	performance	effort
171	temporal demand	performance	performance	effort
172	frustration level	performance	performance	frustration level
173	temporal demand	performance	performance	effort
174	temporal demand	effort	frustration level	effort
175	frustration level	performance	performance	frustration level
176	frustration level	performance	performance	effort
177	frustration level	effort	performance	frustration level
178	temporal demand	performance	performance	frustration level
179	temporal demand	effort	performance	effort
180	temporal demand	performance	frustration level	frustration level

ID	temporal demand VS frustration level	effort VS performance	performance VS frustration level	effort VS frustration level
181	temporal demand	performance	performance	frustration level
182	frustration level	performance	performance	effort
183	frustration level	effort	performance	effort
184	temporal demand	performance	performance	frustration level
185	temporal demand	effort	performance	effort
186	temporal demand	performance	performance	effort
187	frustration level	performance	frustration level	frustration level
188	temporal demand	performance	frustration level	frustration level
189	temporal demand	effort	performance	effort
190	temporal demand	performance	performance	effort
191	temporal demand	performance	performance	effort
192	frustration level	performance	frustration level	frustration level
193	frustration level	performance	performance	frustration level
194	temporal demand	performance	performance	frustration level
195	temporal demand	effort	frustration level	effort
196	temporal demand	performance	frustration level	frustration level
197	frustration level	effort	frustration level	frustration level
198	temporal demand	performance	performance	effort
199	frustration level	performance	performance	effort
200	frustration level	effort	performance	frustration level
201	temporal demand	performance	performance	effort
202	temporal demand	performance	performance	frustration level
203	temporal demand	performance	performance	effort
204	frustration level	effort	frustration level	frustration level
205	temporal demand	performance	performance	frustration level
206	temporal demand	effort	performance	effort
207	temporal demand	performance	performance	frustration level
208	temporal demand	performance	performance	effort
209	temporal demand	performance	performance	effort
210	frustration level	effort	performance	frustration level
211	frustration level	effort	performance	frustration level
212	frustration level	performance	frustration level	frustration level
213	temporal demand	performance	performance	effort
214	temporal demand	effort	performance	effort
215	frustration level	effort	frustration level	effort
216	temporal demand	effort	performance	effort

ID	temporal demand VS frustration level	effort VS performance	performance VS frustration level	effort VS frustration level
217	frustration level	performance	performance	effort
218	frustration level	performance	frustration level	frustration level
219	temporal demand	performance	performance	effort
220	frustration level	effort	frustration level	frustration level
221	frustration level	performance	performance	frustration level
222	temporal demand	performance	performance	effort
223	temporal demand	effort	frustration level	frustration level
224	frustration level	effort	performance	effort
225	temporal demand	performance	performance	frustration level
226	temporal demand	effort	frustration level	frustration level
227	frustration level	effort	frustration level	effort
228	frustration level	effort	frustration level	effort
229	temporal demand	performance	performance	frustration level
230	frustration level	performance	performance	effort
231	temporal demand	performance	performance	effort
232	temporal demand	effort	performance	effort
233	temporal demand	performance	frustration level	frustration level
234	frustration level	effort	frustration level	frustration level
235	temporal demand	effort	performance	effort
236	temporal demand	effort	performance	frustration level
237	temporal demand	effort	performance	effort
238	temporal demand	performance	performance	effort
239	frustration level	performance	performance	frustration level
240	frustration level	effort	performance	effort
241	temporal demand	performance	performance	effort
242	temporal demand	performance	performance	frustration level
243	temporal demand	performance	performance	effort
244	temporal demand	performance	performance	effort
245	temporal demand	performance	frustration level	effort
246	frustration level	effort	frustration level	frustration level
247	frustration level	performance	performance	frustration level
248	frustration level	effort	performance	effort
249	temporal demand	performance	performance	effort
250	temporal demand	effort	performance	effort
251	temporal demand	effort	performance	effort
252	temporal demand	performance	frustration level	effort

ID	temporal demand VS frustration level	effort VS performance	performance VS frustration level	effort VS frustration level
253	temporal demand	performance	performance	effort
254	frustration level	performance	performance	frustration level
255	frustration level	performance	performance	frustration level
256	frustration level	performance	performance	effort
257	temporal demand	performance	performance	frustration level
258	temporal demand	performance	performance	effort
259	temporal demand	performance	performance	frustration level
260	frustration level	performance	performance	effort
261	frustration level	effort	frustration level	frustration level
262	frustration level	performance	frustration level	effort
263	frustration level	performance	performance	effort
264	temporal demand	performance	performance	frustration level
265	temporal demand	performance	performance	frustration level
266	frustration level	performance	frustration level	effort
267	frustration level	effort	frustration level	frustration level
268	temporal demand	performance	performance	effort
269	temporal demand	effort	performance	effort
270	temporal demand	performance	performance	effort
271	temporal demand	effort	performance	effort
272	temporal demand	performance	performance	frustration level
273	temporal demand	effort	performance	effort
274	temporal demand	performance	performance	effort
275	frustration level	performance	performance	frustration level
276	temporal demand	performance	performance	frustration level
277	temporal demand	performance	performance	effort
278	temporal demand	performance	frustration level	frustration level
279	frustration level	performance	frustration level	frustration level
280	temporal demand	performance	frustration level	effort
281	temporal demand	performance	performance	frustration level
282	temporal demand	performance	performance	effort
283	temporal demand	performance	performance	frustration level
284	frustration level	performance	frustration level	frustration level
285	frustration level	performance	frustration level	frustration level
286	temporal demand	performance	performance	effort
287	temporal demand	performance	performance	effort
288	temporal demand	effort	performance	effort

ID	temporal demand VS frustration level	effort VS performance	performance VS frustration level	effort VS frustration level
289	frustration level	performance	performance	frustration level
290	frustration level	performance	frustration level	frustration level
291	temporal demand	performance	performance	frustration level
292	temporal demand	effort	performance	effort
293	frustration level	performance	performance	frustration level
294	temporal demand	performance	performance	effort
295	frustration level	effort	frustration level	frustration level
296	temporal demand	performance	performance	effort
297	frustration level	performance	performance	frustration level
298	frustration level	effort	frustration level	frustration level
299	temporal demand	performance	performance	frustration level
300	temporal demand	performance	performance	frustration level
301	frustration level	performance	performance	frustration level
302	temporal demand	performance	performance	frustration level
303	temporal demand	performance	performance	effort
304	frustration level	performance	performance	effort
305	temporal demand	effort	performance	effort
306	frustration level	effort	frustration level	effort
307	frustration level	performance	performance	frustration level
308	temporal demand	effort	performance	effort
309	frustration level	effort	performance	frustration level
310	temporal demand	effort	frustration level	effort
311	frustration level	performance	frustration level	frustration level
312	frustration level	performance	performance	frustration level
313	temporal demand	performance	performance	frustration level
314	frustration level	performance	performance	frustration level
315	frustration level	performance	performance	frustration level
316	frustration level	performance	frustration level	effort
317	temporal demand	performance	performance	effort
318	temporal demand	performance	performance	frustration level
319	temporal demand	effort	performance	frustration level
320	temporal demand	effort	performance	effort
321	frustration level	performance	performance	frustration level
322	temporal demand	effort	frustration level	effort
323	temporal demand	effort	performance	effort
324	temporal demand	performance	performance	effort

ID	temporal demand VS frustration level	effort VS performance	performance VS frustration level	effort VS frustration level
325	temporal demand	performance	performance	effort
326	temporal demand	performance	frustration level	frustration level
327	temporal demand	performance	performance	frustration level
328	temporal demand	performance	frustration level	effort
329	frustration level	performance	performance	effort
330	frustration level	performance	frustration level	frustration level
331	temporal demand	effort	performance	effort
332	frustration level	effort	performance	frustration level
333	frustration level	performance	frustration level	frustration level
334	frustration level	performance	frustration level	effort
335	temporal demand	effort	performance	effort
336	frustration level	performance	performance	frustration level
337	frustration level	effort	performance	effort
338	frustration level	performance	performance	effort
339	frustration level	effort	performance	effort
340	temporal demand	performance	performance	effort
341	temporal demand	performance	performance	effort
342	frustration level	effort	frustration level	frustration level
343	temporal demand	performance	performance	effort
344	temporal demand	effort	performance	effort
345	temporal demand	performance	performance	effort
346	frustration level	performance	performance	frustration level
347	frustration level	effort	frustration level	effort
348	frustration level	performance	frustration level	frustration level
349	temporal demand	performance	frustration level	effort
350	frustration level	performance	frustration level	frustration level
351	frustration level	effort	frustration level	effort
352	temporal demand	performance	performance	effort
353	temporal demand	performance	frustration level	effort
354	temporal demand	performance	performance	effort
355	frustration level	performance	performance	frustration level
356	temporal demand	performance	performance	effort
357	temporal demand	performance	performance	effort
358	frustration level	effort	frustration level	effort
359	frustration level	effort	performance	effort
360	frustration level	effort	frustration level	frustration level

ID	temporal demand VS frustration level	effort VS performance	performance VS frustration level	effort VS frustration level
361	frustration level	performance	performance	frustration level
362	temporal demand	performance	performance	effort
363	temporal demand	effort	frustration level	effort
364	frustration level	performance	performance	frustration level
365	frustration level	performance	performance	frustration level
366	frustration level	effort	frustration level	frustration level
367	frustration level	effort	frustration level	frustration level
368	temporal demand	performance	performance	effort
369	frustration level	performance	performance	effort
370	temporal demand	performance	performance	effort
371	frustration level	performance	performance	frustration level
372	temporal demand	performance	performance	effort
373	temporal demand	performance	performance	effort
374	frustration level	performance	frustration level	effort
375	frustration level	performance	frustration level	effort
376	frustration level	performance	frustration level	frustration level
377	temporal demand	effort	frustration level	frustration level
378	temporal demand	effort	frustration level	frustration level
379	frustration level	effort	frustration level	frustration level
380	frustration level	effort	frustration level	effort
381	temporal demand	performance	performance	frustration level
382	frustration level	performance	performance	frustration level
383	temporal demand	performance	performance	frustration level
384	temporal demand	performance	performance	effort
385	frustration level	performance	performance	frustration level
386	temporal demand	performance	performance	effort
387	frustration level	performance	performance	frustration level
388	frustration level	performance	performance	effort
389	temporal demand	performance	frustration level	frustration level
390	temporal demand	performance	performance	effort
391	frustration level	effort	performance	effort
392	temporal demand	effort	performance	effort
393	temporal demand	effort	performance	effort
394	temporal demand	performance	performance	effort
395	frustration level	effort	performance	effort
396	temporal demand	performance	performance	effort

ID	temporal demand VS frustration level	effort VS performance	performance VS frustration level	effort VS frustration level
397	temporal demand	effort	frustration level	frustration level
398	frustration level	performance	performance	effort
399	frustration level	performance	frustration level	effort
400	temporal demand	effort	performance	effort
401	temporal demand	performance	performance	effort
402	frustration level	performance	performance	effort
403	temporal demand	effort	frustration level	effort
404	temporal demand	effort	frustration level	effort
405	temporal demand	performance	performance	frustration level
406	temporal demand	performance	performance	frustration level
407	frustration level	performance	performance	frustration level
408	frustration level	performance	performance	effort
409	temporal demand	performance	frustration level	effort
410	frustration level	effort	frustration level	effort
411	frustration level	performance	frustration level	frustration level
412	frustration level	effort	performance	frustration level
413	temporal demand	performance	performance	frustration level
414	frustration level	performance	frustration level	effort
415	temporal demand	performance	performance	effort
416	temporal demand	performance	performance	frustration level
417	frustration level	performance	frustration level	frustration level
418	frustration level	performance	performance	effort
419	frustration level	performance	frustration level	frustration level
420	frustration level	performance	frustration level	frustration level
421	temporal demand	performance	performance	effort
422	temporal demand	performance	performance	effort
423	temporal demand	effort	performance	effort
424	temporal demand	effort	performance	effort
425	frustration level	performance	performance	effort
426	temporal demand	performance	frustration level	frustration level
427	temporal demand	performance	performance	effort
428	temporal demand	performance	performance	frustration level
429	frustration level	effort	performance	frustration level
430	temporal demand	performance	performance	effort
431	frustration level	effort	frustration level	effort
432	frustration level	performance	performance	frustration level

ID	temporal demand VS frustration level	effort VS performance	performance VS frustration level	effort VS frustration level
433	temporal demand	performance	performance	effort
434	frustration level	performance	performance	effort
435	frustration level	effort	frustration level	frustration level
436	temporal demand	performance	performance	effort
437	temporal demand	performance	performance	frustration level
438	frustration level	effort	performance	effort
439	frustration level	performance	frustration level	frustration level
440	temporal demand	effort	frustration level	effort
441	temporal demand	effort	performance	effort
442	temporal demand	performance	frustration level	frustration level
443	temporal demand	effort	performance	effort
444	temporal demand	performance	performance	effort
445	temporal demand	effort	performance	effort
446	temporal demand	effort	performance	effort
447	temporal demand	performance	frustration level	effort
448	temporal demand	performance	frustration level	frustration level
449	frustration level	effort	performance	effort
450	frustration level	effort	frustration level	frustration level
451	frustration level	performance	performance	effort
452	temporal demand	effort	performance	effort
453	temporal demand	effort	performance	effort
454	frustration level	effort	frustration level	effort
455	temporal demand	performance	performance	effort
456	frustration level	performance	performance	effort
457	frustration level	performance	performance	frustration level
458	temporal demand	performance	performance	effort
459	frustration level	effort	performance	effort
460	frustration level	performance	performance	effort
461	temporal demand	performance	performance	effort
462	frustration level	performance	performance	effort
463	frustration level	performance	performance	frustration level
464	frustration level	performance	performance	frustration level
465	frustration level	effort	performance	effort
466	frustration level	effort	performance	frustration level
467	temporal demand	performance	performance	effort
468	temporal demand	performance	performance	frustration level

ID	temporal demand VS frustration level	effort VS performance	performance VS frustration level	effort VS frustration level
469	temporal demand	performance	performance	effort
470	temporal demand	performance	performance	effort
471	temporal demand	effort	performance	effort
472	temporal demand	effort	frustration level	effort
473	temporal demand	performance	performance	effort
474	frustration level	performance	performance	frustration level
475	temporal demand	performance	performance	frustration level
476	temporal demand	effort	performance	effort
477	frustration level	performance	performance	effort
478	frustration level	performance	performance	frustration level
479	temporal demand	performance	performance	frustration level
480	temporal demand	performance	performance	frustration level
481	temporal demand	effort	frustration level	effort
482	temporal demand	performance	performance	frustration level
483	frustration level	effort	performance	frustration level
484	temporal demand	effort	performance	effort
485	temporal demand	performance	performance	effort
486	temporal demand	effort	performance	effort
487	temporal demand	performance	performance	effort
488	frustration level	effort	frustration level	frustration level
489	temporal demand	effort	performance	effort
490	frustration level	performance	performance	effort
491	frustration level	performance	performance	effort
492	temporal demand	performance	frustration level	frustration level
493	frustration level	performance	performance	effort
494	temporal demand	performance	performance	effort
495	temporal demand	effort	performance	effort
496	frustration level	effort	performance	effort
497	temporal demand	effort	performance	frustration level
498	frustration level	effort	frustration level	effort
499	frustration level	performance	performance	frustration level
500	temporal demand	performance	performance	effort
501	temporal demand	performance	performance	effort
502	temporal demand	effort	performance	effort
503	temporal demand	performance	performance	effort
504	temporal demand	performance	performance	effort

ID	temporal demand VS frustration level	effort VS performance	performance VS frustration level	effort VS frustration level
505	temporal demand	performance	performance	effort
506	temporal demand	performance	performance	effort
507	frustration level	performance	performance	frustration level
508	frustration level	effort	frustration level	effort
509	frustration level	effort	frustration level	frustration level
510	temporal demand	effort	frustration level	effort
511	temporal demand	effort	frustration level	effort
512	temporal demand	effort	performance	effort
513	frustration level	performance	performance	frustration level
514	temporal demand	performance	performance	effort
515	frustration level	performance	performance	effort
516	temporal demand	performance	performance	effort
517	frustration level	performance	frustration level	effort
518	frustration level	performance	frustration level	frustration level
519	temporal demand	performance	performance	frustration level
520	temporal demand	effort	performance	effort
521	frustration level	effort	performance	effort
522	frustration level	effort	performance	effort
523	frustration level	effort	performance	effort
524	temporal demand	effort	performance	frustration level
525	temporal demand	effort	performance	effort
526	frustration level	performance	performance	effort
527	frustration level	effort	frustration level	effort
528	temporal demand	performance	performance	effort
529	frustration level	performance	performance	effort
530	temporal demand	performance	performance	effort
531	temporal demand	performance	frustration level	effort
532	frustration level	effort	performance	effort
533	temporal demand	effort	performance	effort
534	frustration level	performance	performance	frustration level
535	temporal demand	performance	performance	frustration level
536	frustration level	effort	performance	effort
537	temporal demand	performance	performance	frustration level
538	temporal demand	performance	frustration level	effort
539	temporal demand	effort	frustration level	effort
540	temporal demand	performance	performance	effort

ID	temporal demand VS frustration level	effort VS performance	performance VS frustration level	effort VS frustration level
541	frustration level	performance	performance	frustration level
542	frustration level	effort	performance	effort
543	temporal demand	performance	performance	frustration level
544	temporal demand	effort	frustration level	effort
545	frustration level	performance	performance	effort
546	frustration level	performance	performance	effort
547	temporal demand	performance	performance	frustration level
548	frustration level	effort	performance	effort
549	temporal demand	performance	performance	effort
550	frustration level	effort	frustration level	effort
551	temporal demand	performance	performance	effort
552	temporal demand	performance	frustration level	frustration level
553	temporal demand	performance	performance	frustration level
554	temporal demand	performance	frustration level	effort
555	frustration level	performance	frustration level	frustration level
556	temporal demand	performance	performance	frustration level
557	frustration level	effort	frustration level	frustration level
558	temporal demand	performance	performance	frustration level
559	temporal demand	effort	performance	frustration level
560	temporal demand	performance	performance	frustration level
561	frustration level	effort	frustration level	frustration level
562	frustration level	performance	frustration level	effort
563	frustration level	performance	performance	effort
564	temporal demand	performance	performance	effort
565	frustration level	performance	performance	effort
566	temporal demand	effort	performance	effort
567	frustration level	performance	frustration level	frustration level
568	frustration level	performance	performance	effort
569	temporal demand	effort	performance	effort
570	frustration level	performance	performance	frustration level
571	frustration level	performance	performance	frustration level
572	temporal demand	performance	performance	effort
573	temporal demand	performance	performance	effort
574	frustration level	performance	performance	frustration level
575	frustration level	performance	performance	frustration level
576	temporal demand	performance	performance	frustration level

ID	temporal demand VS frustration level	effort VS performance	performance VS frustration level	effort VS frustration level
577	temporal demand	performance	performance	effort
578	temporal demand	performance	performance	frustration level
579	temporal demand	performance	frustration level	effort
580	temporal demand	effort	performance	effort
581	frustration level	performance	performance	frustration level
582	frustration level	performance	performance	frustration level
583	temporal demand	performance	performance	effort
584	frustration level	performance	frustration level	effort
585	temporal demand	effort	performance	frustration level
586	frustration level	performance	frustration level	effort
587	temporal demand	effort	performance	effort
588	frustration level	performance	performance	effort
589	temporal demand	performance	performance	frustration level
590	temporal demand	performance	frustration level	frustration level
591	temporal demand	effort	performance	effort
592	temporal demand	performance	performance	frustration level
593	temporal demand	performance	frustration level	frustration level
594	frustration level	performance	performance	frustration level
595	temporal demand	effort	frustration level	effort
596	temporal demand	performance	performance	frustration level
597	frustration level	performance	performance	effort
598	temporal demand	effort	performance	effort
599	temporal demand	performance	performance	frustration level
600	frustration level	performance	frustration level	frustration level
601	frustration level	effort	performance	frustration level
602	frustration level	performance	performance	frustration level
603	temporal demand	effort	performance	frustration level
604	frustration level	performance	performance	frustration level
605	temporal demand	performance	frustration level	effort
606	temporal demand	effort	frustration level	frustration level
607	frustration level	performance	performance	frustration level
608	temporal demand	performance	performance	effort
609	temporal demand	performance	performance	effort
610	frustration level	performance	performance	frustration level
611	temporal demand	performance	frustration level	effort
612	temporal demand	performance	performance	effort

ID	temporal demand<br>VS<br>frustration level	effort<br>VS<br>performance	performance<br>VS<br>frustration level	effort<br>VS<br>frustration level
613	frustration level	effort	frustration level	frustration level
614	frustration level	effort	frustration level	frustration level
615	temporal demand	performance	performance	effort
616	frustration level	effort	frustration level	frustration level
617	frustration level	effort	performance	frustration level
618	temporal demand	effort	frustration level	frustration level
619	frustration level	effort	frustration level	effort
620	temporal demand	performance	performance	frustration level
621	frustration level	effort	performance	effort
622	temporal demand	effort	performance	effort
623	frustration level	performance	performance	frustration level
624	frustration level	performance	frustration level	frustration level
625	temporal demand	performance	performance	effort
626	temporal demand	performance	performance	effort
627	frustration level	performance	performance	effort
628	temporal demand	effort	frustration level	effort
629	frustration level	performance	performance	effort
630	temporal demand	performance	frustration level	frustration level
631	frustration level	performance	performance	frustration level
632	frustration level	effort	performance	effort
633	frustration level	effort	performance	effort
634	frustration level	performance	performance	effort
635	temporal demand	performance	performance	effort
636	temporal demand	performance	performance	frustration level
637	temporal demand	performance	performance	effort
638	temporal demand	performance	performance	effort
639	temporal demand	performance	frustration level	frustration level
640	frustration level	performance	performance	effort
641	frustration level	performance	performance	effort
642	frustration level	performance	performance	frustration level
643	temporal demand	effort	performance	effort
644	frustration level	performance	performance	effort
645	frustration level	effort	performance	frustration level
646	temporal demand	performance	performance	effort
647	temporal demand	performance	performance	frustration level
648	temporal demand	performance	performance	effort

ID	temporal demand VS frustration level	effort VS performance	performance VS frustration level	effort VS frustration level
649	frustration level	performance	performance	frustration level
650	temporal demand	performance	performance	frustration level
651	temporal demand	effort	performance	effort
652	temporal demand	performance	performance	effort
653	temporal demand	performance	performance	effort
654	frustration level	performance	performance	frustration level
655	temporal demand	performance	performance	effort
656	frustration level	performance	frustration level	frustration level
657	temporal demand	performance	frustration level	frustration level
658	frustration level	effort	frustration level	frustration level
659	temporal demand	effort	performance	effort
660	frustration level	performance	performance	frustration level
661	frustration level	performance	performance	frustration level
662	frustration level	effort	frustration level	effort
663	temporal demand	performance	performance	effort
664	frustration level	performance	performance	effort
665	temporal demand	performance	performance	effort
666	frustration level	performance	performance	frustration level
667	temporal demand	performance	performance	effort
668	temporal demand	performance	frustration level	frustration level
669	frustration level	effort	performance	effort
670	frustration level	performance	frustration level	frustration level
671	temporal demand	performance	performance	effort
672	temporal demand	performance	frustration level	frustration level
673	frustration level	effort	frustration level	effort
674	temporal demand	performance	performance	effort
675	temporal demand	effort	frustration level	effort
676	temporal demand	performance	performance	effort
677	temporal demand	performance	performance	frustration level
678	temporal demand	performance	performance	effort
679	frustration level	effort	frustration level	frustration level
680	frustration level	effort	frustration level	frustration level
681	frustration level	effort	performance	frustration level
682	frustration level	performance	performance	frustration level
683	frustration level	performance	frustration level	effort
684	frustration level	performance	performance	effort

ID	temporal demand VS frustration level	effort VS performance	performance VS frustration level	effort VS frustration level
685	frustration level	effort	performance	effort
686	temporal demand	effort	frustration level	frustration level
687	frustration level	effort	performance	effort
688	temporal demand	performance	performance	effort
689	temporal demand	effort	performance	effort
690	frustration level	effort	frustration level	frustration level
691	temporal demand	performance	performance	effort
692	temporal demand	performance	performance	frustration level
693	frustration level	performance	performance	effort
694	temporal demand	effort	performance	effort
695	frustration level	effort	frustration level	frustration level
696	temporal demand	performance	frustration level	effort
697	temporal demand	performance	performance	effort
698	temporal demand	effort	frustration level	frustration level
699	temporal demand	effort	performance	effort
700	temporal demand	performance	performance	effort
701	frustration level	performance	performance	effort
702	temporal demand	performance	performance	frustration level
703	temporal demand	performance	performance	frustration level
704	frustration level	effort	performance	effort
705	frustration level	effort	frustration level	frustration level
706	frustration level	effort	performance	frustration level
707	temporal demand	effort	performance	effort
708	frustration level	performance	frustration level	frustration level
709	temporal demand	performance	performance	effort
710	frustration level	performance	frustration level	frustration level
711	frustration level	performance	performance	effort
712	temporal demand	performance	frustration level	frustration level
713	temporal demand	performance	performance	effort
714	temporal demand	effort	frustration level	frustration level
715	frustration level	performance	frustration level	effort
716	frustration level	performance	performance	frustration level
717	frustration level	performance	performance	effort
718	temporal demand	effort	frustration level	frustration level
719	frustration level	performance	performance	frustration level
720	temporal demand	performance	frustration level	effort

ID	temporal demand VS frustration level	effort VS performance	performance VS frustration level	effort VS frustration level
721	temporal demand	performance	performance	frustration level
722	frustration level	effort	frustration level	frustration level
723	frustration level	performance	performance	frustration level
724	frustration level	effort	performance	effort
725	temporal demand	effort	performance	effort
726	temporal demand	performance	frustration level	frustration level
727	temporal demand	effort	performance	effort
728	temporal demand	effort	performance	effort
729	temporal demand	effort	frustration level	effort
730	temporal demand	effort	performance	effort
731	temporal demand	performance	performance	frustration level
732	temporal demand	performance	performance	effort
733	frustration level	performance	performance	effort
734	frustration level	performance	frustration level	frustration level
735	temporal demand	performance	performance	effort
736	temporal demand	performance	performance	effort
737	frustration level	performance	frustration level	frustration level
738	temporal demand	performance	performance	frustration level
739	frustration level	performance	frustration level	frustration level
740	temporal demand	performance	performance	effort
741	frustration level	effort	performance	effort
742	frustration level	effort	performance	frustration level
743	frustration level	performance	frustration level	effort
744	temporal demand	performance	frustration level	frustration level
745	frustration level	performance	frustration level	effort
746	frustration level	performance	performance	frustration level
747	temporal demand	performance	performance	effort
748	temporal demand	performance	performance	effort
749	frustration level	effort	frustration level	frustration level
750	frustration level	performance	performance	effort
751	frustration level	performance	performance	effort
752	frustration level	performance	performance	frustration level
753	temporal demand	performance	performance	frustration level
754	frustration level	performance	performance	effort
755	frustration level	effort	frustration level	frustration level
756	temporal demand	performance	frustration level	frustration level

ID	temporal demand VS frustration level	effort VS performance	performance VS frustration level	effort VS frustration level
757	frustration level	performance	performance	effort
758	temporal demand	performance	performance	effort
759	temporal demand	effort	performance	effort
760	frustration level	performance	performance	frustration level
761	temporal demand	performance	performance	effort
762	frustration level	performance	frustration level	frustration level
763	temporal demand	performance	performance	frustration level
764	temporal demand	effort	frustration level	effort
765	temporal demand	performance	performance	frustration level
766	temporal demand	performance	performance	frustration level
767	temporal demand	effort	performance	effort
768	frustration level	performance	performance	frustration level
769	temporal demand	performance	performance	effort
770	frustration level	performance	performance	frustration level
771	frustration level	performance	frustration level	frustration level
772	temporal demand	performance	performance	frustration level
773	frustration level	performance	frustration level	frustration level
774	temporal demand	performance	performance	frustration level
775	frustration level	performance	performance	frustration level
776	temporal demand	performance	performance	effort
777	temporal demand	performance	frustration level	frustration level
778	temporal demand	effort	frustration level	frustration level
779	temporal demand	performance	performance	frustration level
780	frustration level	effort	frustration level	effort
781	frustration level	performance	frustration level	frustration level
782	temporal demand	effort	frustration level	frustration level
783	frustration level	performance	performance	frustration level
784	frustration level	performance	frustration level	frustration level
785	frustration level	performance	frustration level	effort
786	frustration level	performance	performance	effort
787	frustration level	effort	performance	effort
788	temporal demand	performance	performance	frustration level
789	temporal demand	performance	performance	effort
790	frustration level	performance	frustration level	effort
791	temporal demand	performance	frustration level	frustration level
792	temporal demand	effort	performance	frustration level

ID	temporal demand VS frustration level	effort VS performance	performance VS frustration level	effort VS frustration level
793	frustration level	performance	performance	frustration level
794	temporal demand	effort	performance	effort
795	frustration level	performance	performance	frustration level
796	temporal demand	effort	performance	effort
797	frustration level	performance	performance	frustration level
798	frustration level	effort	frustration level	frustration level
799	frustration level	effort	performance	effort
800	temporal demand	performance	performance	effort
801	temporal demand	performance	performance	effort
802	temporal demand	performance	frustration level	frustration level
803	temporal demand	effort	performance	effort
804	temporal demand	performance	performance	effort
805	temporal demand	performance	performance	frustration level
806	frustration level	effort	frustration level	effort
807	frustration level	effort	frustration level	frustration level
808	frustration level	effort	frustration level	frustration level
809	temporal demand	performance	performance	frustration level
810	temporal demand	effort	performance	effort
811	frustration level	performance	frustration level	frustration level
812	temporal demand	performance	performance	effort
813	frustration level	performance	frustration level	frustration level
814	frustration level	effort	frustration level	effort
815	temporal demand	performance	frustration level	frustration level
816	frustration level	effort	frustration level	frustration level
817	frustration level	performance	performance	frustration level
818	frustration level	effort	frustration level	frustration level
819	frustration level	effort	performance	frustration level
820	temporal demand	effort	performance	effort
821	temporal demand	effort	performance	effort
822	temporal demand	performance	performance	effort
823	temporal demand	performance	frustration level	frustration level
824	frustration level	effort	frustration level	frustration level
825	temporal demand	performance	performance	effort
826	frustration level	performance	performance	effort
827	temporal demand	performance	performance	effort
828	frustration level	performance	performance	effort

ID	temporal demand<br>VS<br>frustration level	effort<br>VS<br>performance	performance<br>VS<br>frustration level	effort<br>VS<br>frustration level
829	frustration level	performance	frustration level	frustration level
830	temporal demand	performance	performance	frustration level
831	temporal demand	effort	performance	frustration level
832	temporal demand	performance	performance	frustration level
833	frustration level	effort	performance	effort
834	temporal demand	effort	frustration level	frustration level
835	frustration level	performance	performance	effort
836	frustration level	effort	performance	frustration level
837	frustration level	performance	performance	effort
838	temporal demand	performance	performance	effort
839	temporal demand	performance	performance	effort
840	frustration level	effort	frustration level	frustration level
841	frustration level	effort	frustration level	effort
842	temporal demand	performance	performance	frustration level
843	frustration level	effort	frustration level	frustration level
844	frustration level	performance	frustration level	frustration level
845	temporal demand	performance	frustration level	frustration level
846	frustration level	effort	performance	effort
847	temporal demand	performance	performance	effort
848	temporal demand	effort	frustration level	frustration level
849	temporal demand	performance	performance	effort
850	frustration level	performance	performance	frustration level
851	frustration level	effort	frustration level	effort
852	frustration level	performance	performance	effort
853	temporal demand	performance	performance	frustration level
854	temporal demand	performance	performance	frustration level
855	temporal demand	effort	frustration level	effort
856	temporal demand	effort	performance	effort
857	temporal demand	performance	performance	effort
858	frustration level	performance	performance	effort
859	frustration level	performance	frustration level	frustration level
860	frustration level	performance	performance	frustration level
861	frustration level	effort	performance	effort
862	frustration level	effort	frustration level	effort
863	frustration level	performance	performance	frustration level
864	frustration level	performance	performance	frustration level

ID	temporal demand VS frustration level	effort VS performance	performance VS frustration level	effort VS frustration level
865	temporal demand	performance	performance	effort
866	frustration level	performance	performance	effort
867	frustration level	performance	performance	effort
868	frustration level	performance	frustration level	frustration level
869	temporal demand	performance	performance	effort
870	frustration level	performance	frustration level	frustration level
871	temporal demand	performance	frustration level	effort
872	temporal demand	performance	performance	frustration level
873	temporal demand	performance	performance	effort
874	frustration level	performance	frustration level	effort
875	temporal demand	effort	performance	effort
876	temporal demand	effort	performance	frustration level
877	temporal demand	performance	performance	effort
878	frustration level	performance	performance	effort
879	temporal demand	performance	performance	frustration level
880	temporal demand	performance	performance	effort
881	temporal demand	performance	performance	frustration level
882	frustration level	effort	frustration level	frustration level
883	temporal demand	performance	performance	effort
884	temporal demand	performance	performance	effort
885	temporal demand	effort	performance	frustration level
886	temporal demand	performance	performance	effort
887	frustration level	effort	frustration level	effort
888	frustration level	effort	frustration level	effort
889	temporal demand	performance	performance	effort
890	temporal demand	effort	performance	effort
891	frustration level	performance	frustration level	frustration level
892	frustration level	effort	frustration level	frustration level
893	frustration level	effort	performance	frustration level
894	frustration level	effort	frustration level	frustration level
895	temporal demand	performance	performance	frustration level
896	temporal demand	performance	performance	effort
897	frustration level	effort	frustration level	frustration level
898	temporal demand	effort	performance	effort
899	frustration level	effort	frustration level	frustration level
900	frustration level	performance	frustration level	effort

ID	temporal demand VS frustration level	effort VS performance	performance VS frustration level	effort VS frustration level
901	frustration level	performance	performance	effort
902	temporal demand	performance	performance	effort
903	frustration level	performance	frustration level	effort
904	temporal demand	effort	performance	effort
905	frustration level	performance	frustration level	frustration level
906	frustration level	effort	frustration level	effort
907	frustration level	performance	performance	effort
908	frustration level	performance	performance	frustration level
909	frustration level	effort	performance	effort
910	temporal demand	effort	performance	effort
911	frustration level	performance	performance	effort
912	temporal demand	effort	performance	effort
913	temporal demand	performance	frustration level	effort
914	frustration level	performance	performance	effort
915	frustration level	performance	performance	effort
916	frustration level	effort	frustration level	effort
917	temporal demand	performance	performance	effort
918	frustration level	effort	frustration level	frustration level
919	temporal demand	performance	performance	frustration level
920	temporal demand	performance	performance	frustration level
921	frustration level	performance	performance	frustration level
922	temporal demand	performance	performance	effort
923	temporal demand	effort	frustration level	frustration level
924	temporal demand	performance	performance	effort
925	frustration level	effort	frustration level	frustration level
926	frustration level	performance	frustration level	frustration level
927	temporal demand	performance	performance	frustration level
928	temporal demand	performance	performance	effort
929	temporal demand	performance	performance	frustration level
930	temporal demand	effort	performance	effort
931	temporal demand	performance	frustration level	frustration level
932	temporal demand	effort	performance	effort
933	temporal demand	effort	performance	effort
934	frustration level	performance	performance	frustration level
935	temporal demand	performance	performance	effort
936	frustration level	performance	performance	effort

ID	temporal demand VS frustration level	effort VS performance	performance VS frustration level	effort VS frustration level
937	temporal demand	performance	performance	effort
938	frustration level	performance	frustration level	frustration level
939	frustration level	performance	performance	effort
940	frustration level	effort	performance	frustration level
941	frustration level	performance	performance	frustration level
942	temporal demand	effort	performance	effort
943	temporal demand	performance	performance	frustration level
944	frustration level	effort	frustration level	frustration level
945	temporal demand	effort	performance	frustration level
946	frustration level	effort	frustration level	effort
947	frustration level	performance	performance	effort
948	temporal demand	performance	frustration level	effort
949	temporal demand	performance	performance	effort
950	frustration level	performance	performance	effort
951	frustration level	performance	frustration level	frustration level
952	frustration level	performance	frustration level	effort
953	frustration level	performance	performance	frustration level
954	frustration level	performance	performance	frustration level
955	temporal demand	performance	frustration level	effort
956	frustration level	effort	frustration level	frustration level
957	frustration level	effort	performance	effort
958	temporal demand	effort	performance	effort
959	frustration level	effort	performance	effort
960	temporal demand	effort	frustration level	frustration level
961	temporal demand	effort	performance	effort
962	temporal demand	performance	performance	effort
963	frustration level	performance	performance	frustration level
964	frustration level	performance	performance	frustration level
965	temporal demand	effort	performance	effort
966	frustration level	effort	frustration level	effort
967	temporal demand	performance	performance	frustration level
968	frustration level	performance	frustration level	frustration level
969	temporal demand	performance	performance	effort
970	temporal demand	effort	performance	effort
971	temporal demand	performance	performance	effort
972	temporal demand	performance	performance	effort

ID	temporal demand VS frustration level	effort VS performance	performance VS frustration level	effort VS frustration level
973	temporal demand	performance	frustration level	effort
974	frustration level	performance	performance	frustration level
975	frustration level	performance	performance	frustration level
976	temporal demand	performance	performance	effort
977	temporal demand	performance	performance	effort
978	temporal demand	performance	frustration level	effort
979	frustration level	effort	frustration level	frustration level
980	frustration level	performance	performance	effort
981	frustration level	performance	performance	effort
982	frustration level	effort	performance	effort
983	temporal demand	performance	performance	effort
984	temporal demand	performance	performance	effort
985	temporal demand	performance	performance	effort
986	temporal demand	performance	performance	frustration level
987	temporal demand	performance	frustration level	frustration level
988	temporal demand	performance	performance	effort
989	temporal demand	performance	performance	effort
990	temporal demand	performance	performance	effort
991	temporal demand	performance	performance	effort
992	temporal demand	performance	frustration level	effort
993	frustration level	performance	frustration level	frustration level
994	temporal demand	performance	performance	effort
995	temporal demand	effort	performance	effort
996	frustration level	effort	frustration level	frustration level
997	temporal demand	performance	performance	frustration level
998	temporal demand	performance	performance	frustration level
999	temporal demand	effort	performance	effort
1000	temporal demand	performance	performance	effort
1001	temporal demand	effort	performance	effort
1002	temporal demand	performance	performance	effort
1003	frustration level	performance	performance	frustration level
1004	frustration level	effort	frustration level	frustration level
