# Supplementary material for: Factoring and correlation in sleep, fatigue and mental workload of clinical first-line nurses in the post-pandemic era of COVID-19: A multi-center cross-sectional study
Source: Front Psychiatry. 2022 Aug 25;13:963419. doi: 10.3389/fpsyt.2022.963419 (PMC9452657; doi:10.3389/fpsyt.2022.963419)
Supplement: Supplementary file 4 [file Table_1.DOCX]

| **Sociodemographic data of the nurses and their statements** | |
| --- | --- |
| **Sociodemographic data** | **Statement** |
| Age/years |  |
| Weight/kg |  |
| Height/m |  |
| BMI | $BMI=\frac{kg}{m^{2}}$ |
| Sex | Male  Female |
| Vision status | Normal  Mild myopia: Eyesight is less than or equal to 300  Moderate myopia: The vision degree is from 300 to 600  High myopia: Visual acuity greater than 600 |
| Current area of practice | The department where the nurse currently works, including internal, surgical, outpatient or emergency & intensive care units. |
| Care model | “Holistic care”, “Functional care”, or “Both holistic and functional care” |
| Professional title | “Junior”, “Intermediate”, “Subsenior”, or “Senior” |
| Education | “Junior college”, “Undergraduate”, or “Postgraduate” |
| Blood type | “A”, “B”, “O”, “AB”, or “Unknown” |
| Marital status | “Single” or “Married” |
| Length of service | “0-5 years”, “6-10 years” or “More than 11 years” |
| ICU nurse | “No” or “Yes” |
| Kids | The number of children the nurse has.  “0”, “1”, “2”, or “3” |
| Night shift nurse | The nurse who has been in rotation for day and night shifts over the past month.  “No” or “Yes” |
| Family history of insomnia | Whether immediate blood relatives, including parents and siblings, have a history of insomnia?  “No”, “Yes” or “Unknown” |
| Insomnia in the past month | “No” or “Yes” |
| Frequency of night shifts in the past month /days | “≤4”, “5-8” or “≥9” |
| Social jet lag | Social jet lag (SJL), the misalignment between biological and social time, is calculated as the average sleeping hours on free days minus the sleeping hours on workdays.  “Negative SJL”: the average sleeping hours on workdays were more than those on free days  “Positive SJL”: the average sleeping hours on workdays were less than those on free days  “Zero SJL”: the average sleeping hours on workdays were the same as those on free days |
| Comparison of sleep quality before and now | The comparison of sleep quality before participating in nursing work and now:  “Better before”, “Better now”, or “Same” |
| Severity of the influence of COVID-19 on workload | How severe is COVID-19 to the workload?  “Severe”, “Serious” or “Minor” |
| Severity of the influence of COVID-19 on sleep quality | How severe is COVID-19 to the sleep quality?  “Severe”, “Serious” or “Minor” |
| Satisfaction with personal environment | How satisfied are you with the current working environment?  “Satisfied”, “Happy” or “Dissatisfied” |
